# Manganese‐Catalyzed β‐Methylation of Alcohols by Methanol

**DOI:** 10.1002/anie.201912055

**Published:** 2019-12-12

**Authors:** Martin Schlagbauer, Fabian Kallmeier, Torsten Irrgang, Rhett Kempe

**Affiliations:** ^1^ Inorganic Chemistry II—Catalyst Design University of Bayreuth 95440 Bayreuth Germany

**Keywords:** alcohols, borrowing hydrogen, manganese, methylation, sustainable chemistry

## Abstract

We report an earth‐abundant‐metal‐catalyzed double and single methylation of alcohols. A manganese catalyst, which operates at low catalyst loadings and short reaction times, mediates these reactions efficiently. A broad scope of primary and secondary alcohols, including purely aliphatic examples, and 1,2‐aminoalcohols can be methylated. Furthermore, alcohol methylation for the synthesis of pharmaceuticals has been demonstrated. The catalyst system tolerates many functional groups among them hydrogenation‐sensitive examples and upscaling is easily achieved. Mechanistic investigations are indicative of a borrowing hydrogen or hydrogen autotransfer mechanism involving a bimetallic K‐Mn catalyst. The catalyst accepts hydrogen as a proton and a hydride from alcohols efficiently and reacts with a chalcone via hydride transfer.

## Graphical abstract

**An Mn‐based catalyst** permits the efficient single methylation of secondary carbon atoms and the double methylation of primary carbon atoms of primary and secondary alcohols, including purely aliphatic examples. The catalyst tolerates many functional groups, operates at low catalyst loadings and short reaction times, and upscaling is easily achieved.

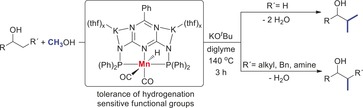

The borrowing hydrogen^1^ or hydrogen autotransfer^2^ methodology (BH/HA) is a prominent and intensively investigated example of an alcohol re‐functionalization concept.^3^, ^4^ The alcohol is catalytically dehydrogenated to a carbonyl compound via hydride and proton transfer to a catalyst, followed by a condensation reaction with a nucleophile and subsequent reduction with the hydrogen (hydride and proton) stored at the catalyst. Alcohols are attractive green and sustainable starting materials for the synthesis of fine and bulk chemicals or agrochemicals and pharmaceuticals^5^ since they can be obtained from abundantly available and indigestible biomass namely lignocellulose combining pyrolysis and hydrogenation.^6^ Furthermore, ethanol can be obtained via fermentation^7^ and methanol via CO_2_ hydrogenation.^8^ The direct hetero‐coupling of two alcohols follows the BH/HA concept and exclusively uses alcohols as starting materials. The replacement of rare metals in key technologies, such as catalysis, is of similar importance as the saving of our fossil carbon resources. Recent progress in manganese‐catalyzed (de)hydrogenation catalysis^9^ indicates the potential of the third most abundant transition metal of the earth crust^10^ to not just replace rare noble metals but to significantly extend their applicability.^11^ Methyl‐group branching is a highly important structural motif in chemistry and biology^12^ ranging from synthetic lubricants^13^ to more than half of all drug molecules.^14^ Thus, BH/HA‐based alkylation reactions employing methanol are especially attractive but challenging due to the increased energy of dehydrogenation to form the transient carbonyl compound. Δ*H* for methanol is +84 kJ mol^−1^ whereas Δ*H* for ethanol is only +68 kJ mol^−1^.^15^

Herein, we report that an earth‐abundant metal catalyst can mediate the double and single methylation of alcohols efficiently. We developed a Mn catalyst that operates at low catalyst loadings (0.1 mol %) and short reaction times (3 h) at temperatures usually used for methanol‐based alkylation reactions. Our catalyst system has a broad scope. The single methylation of secondary carbon atoms and the double methylation of primary carbon atoms of alcohols, including purely aliphatic examples, is observed. Furthermore, methylation of 1,2‐aminoalcohol for the synthesis of pharmaceuticals has been demonstrated. The catalyst system tolerates many functional groups among them hydrogenation‐sensitive examples, like an iodide and a C=C double bond, and permits easy upscaling. Mechanistic investigations are indicative of a borrowing hydrogen or hydrogen autotransfer mechanism involving a bimetallic K‐Mn catalyst. The catalyst accepts a hydride and a proton from alcohols and reacts with chalcone via hydride transfer.

The methylation of alcohols employing methanol has been demonstrated using noble metal catalysts^17^ (Figure 1) and we first described the methanol‐based methylation of 1‐phenylethanol as part of our Mn‐catalyzed multi‐component pyrimidine synthesis.^18^ Parallel to the finalization of our manuscript, Morrill/Williams and co‐workers elegantly demonstrated the Fe‐catalyzed methylation of secondary β‐carbon atoms of alcohols.^16^ Their catalyst system is inefficient in the double methylation of primary β‐carbon atoms of alcohols. Moreover, the alkylation of alcohols by alcohols has been shown for a few earth‐abundant metal catalysts^19^ and the Mn‐catalyzed methylation of substrates other than alcohols has been shown recently.^20^

**Figure 1:**
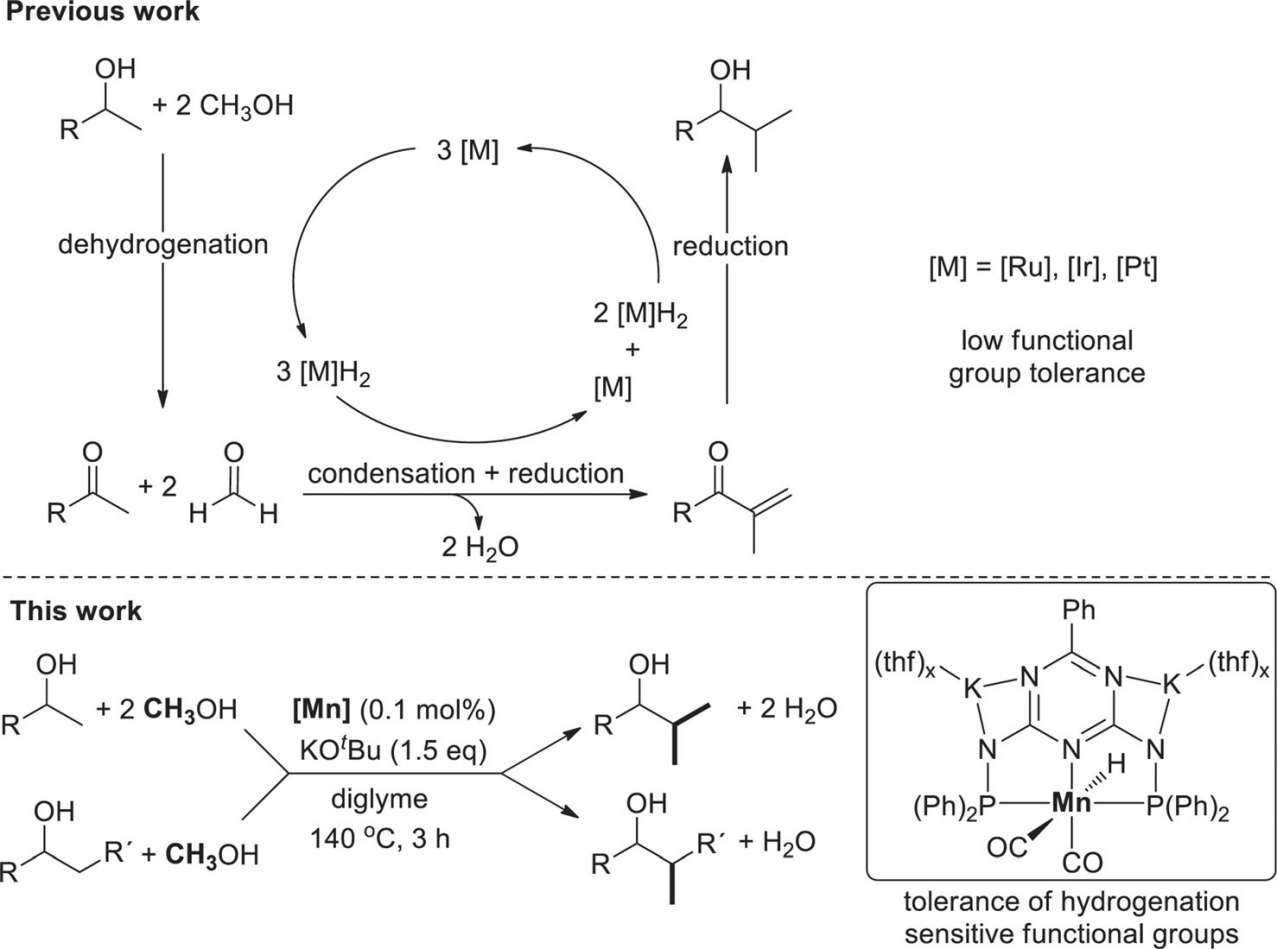
State of the art in double methylation of alcohols employing methanol (top) and the process described in this manuscript (bottom). The groups of Beller17f and Leitner17a described homogenous Ru catalysts. Obora and co‐workers17d and the groups of Xu and Mu17b described heterogeneous Ir catalysts and Shimizu and co‐workers17c described a heterogeneous Pt catalyst. Parallel to the finalization of our manuscript Morrill/Williams and co‐workers described the iron‐complex‐catalyzed methylation of secondary β‐carbon atoms of mostly 2‐arylethanols by methanol.^16^

We investigated the double methylation of 1‐phenyethanol by methanol as a benchmark reaction to optimize the reaction conditions. First, different earth‐abundant metal catalysts were tested at conditions commonly used for methanol‐based alkylation reactions. It is shown that manganese(I) complexes containing a triazine backbone significantly outperform other 3d‐metal‐based precatalysts (Table 1, entries 1–9). Catalytic performance can be further enhanced by changing the isopropyl substituent on the phosphorous atoms to phenyl substituents. Overall, the highest performance was achieved using precatalyst **[Mn‐IIIa]**, which gave the desired product in 63 % yield (Table 1, entry 4). Having established the most active precatalyst, the reaction conditions were then optimized one factor at a time (precatalyst loading, solvent, base, amount of base, temperature, solvent and methanol quantity, and reaction time; see the Supporting Information for details). The best yield (90 %) of **B1** (2‐methyl‐1‐phenylpropan‐1‐ol) was obtained when **[Mn‐IIIa]** was applied with a catalyst loading of 0.1 mol %, a base loading of 1.5 equiv KO^*t*^Bu, 3 equiv methanol, and 2 mL diglyme after 3 h at 140 °C.

**Table 1:** Precatalyst screening.^[a]^ 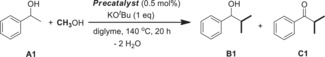

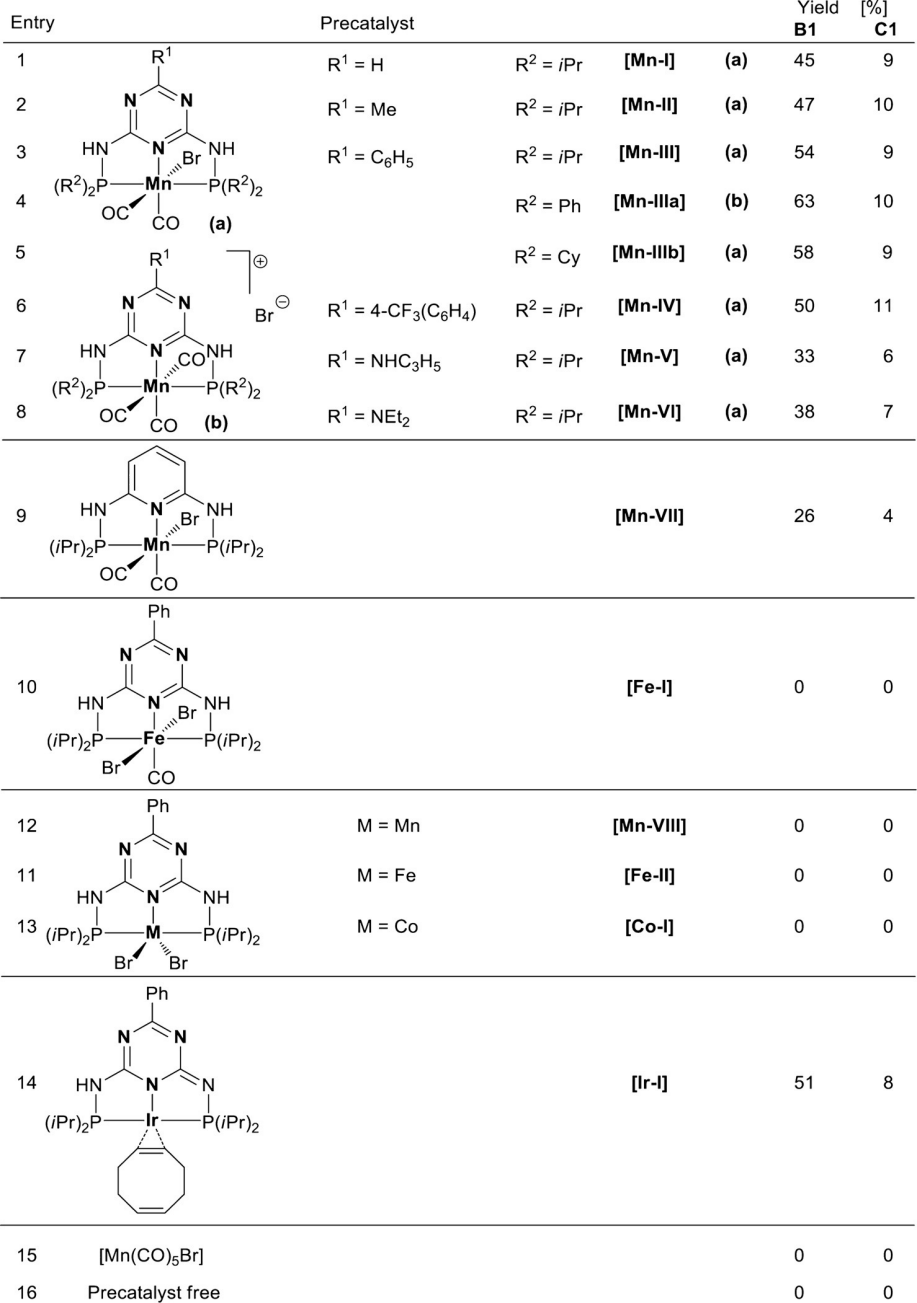

[a] Reaction conditions: 0.5 mol % precatalyst (5 μmol), KO^*t*^Bu (1 mmol, 112 mg), **A1** (1 mmol, 121 μL), MeOH (3 mmol, 122 μL), diglyme (2 mL), 140 °C (oil bath), 20 h. Yields of **B1** and **C1** were determined by GC‐analysis using *n*‐decane as an internal standard.

The most active precatalyst **[Mn‐IIIa]** has not yet been reported in literature and was characterized by standard NMR techniques, elemental analysis, and IR spectroscopy. The latter revealed signals typically associated with three carbonyl ligands. This was confirmed by X‐ray crystal structure analysis (Figure 2), which shows that the central manganese atom is meridionally coordinated by the *P*,*N*,*P* ligand and three further carbonyl ligands, which form a slightly distorted octahedral coordination pattern. Contrary to the isopropyl (**[Mn‐III]**) and cyclohexyl (**[Mn‐IIIb]**) analogues, the resulting positive charge on the manganese complex is compensated by the bromide counter ion, which is not bound to the manganese center.

**Figure 2:**
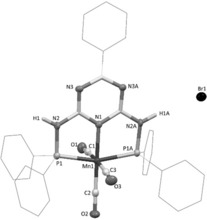
Molecular structure of **[Mn‐IIIa]** with 50 % probability of thermal ellipsoids.^21^ Carbon‐bound hydrogen atoms are omitted for clarity. Selected bond lengths (Å) and angles (°): Mn1–N1 2.028 (4), Mn1–P1 2.2417 (9), Mn1–C1 1.861 (5), Mn1–C2 1.781 (5), Mn1–C3 1.856 (5), P1–N2 1.708 (3), O1–C1 1.136 (5), O2–C2 1.161 (5), O3–C3 1.135 (5); P1‐Mn1‐P1A 162.38 (5), N1‐Mn1‐P1 81.33 (3), N2‐P1‐Mn1 101.58 (10), N1‐Mn1‐C2 177.95 (19), N1‐Mn1‐C1 92.99 (17), N1‐Mn1‐C3 91.01 (17), P1‐Mn1‐C1 88.90 (4), P1‐Mn1‐C2 98.72 (3), P1‐Mn1‐C3 91.71 (4), C1‐Mn1‐C3 176.0 (2), C1‐Mn1‐C2 89.1 (2), C2‐Mn1‐C3 86.9 (2).

With the optimized reaction conditions at hand and the precatalyst characterized, we started to investigate the substrate scope of our catalyst system using a range of differently substituted 1‐arylethanols (Table 2). The yields are similar for *meta*‐ or *para*‐substituted isomers as shown for methyl (*m*: 94 % **B5** and *p*: 89 % **B6**) and methoxy groups (*m*: 92 % **B3** and *p*: 96 % **B4**). A slight decrease in yield, possibly due to steric reasons was observed for the *ortho*‐methoxy‐substituted isomer (*o*: 45 % **B2**). Halide‐substituted 1‐phenylethanols are swiftly converted to the corresponding products in yields up to 80 % for **B8**. After modifying the reaction conditions to 0.3 mol % catalyst loading (same temperature and reaction time), the 3′‐F, 3′‐Br, and 3′‐I analogs could also be obtained in synthetically useful yields (**B7**, **B9**, and **B10**, respectively). The products containing electron‐withdrawing CF_3_ group (74 % **B11**) or electron‐donating groups like *tert*‐butyl (80 % **B12**) and 1‐pyrrolidinyl (56 % **B14**) were easily obtained in high yields. Naphthyl ethanols could be methylated in attractive yields of 79 % for **B15** and 96 % for **B16**. Heterocyclic moieties like 3‐pyridine and ferrocene appear to be tolerated, giving the corresponding products in 72 % **B17** and 76 % **B18** yields, respectively. Gratifyingly, the synthesis is easily scaled up, which was demonstrated by synthesizing **B4** on a 47.6 mmol scale yielding 7.46 g (87 %) product after distillation.

**Table 2:** Substrate scope for the double methylation at the primary C‐atom of secondary alcohols.^[a]^

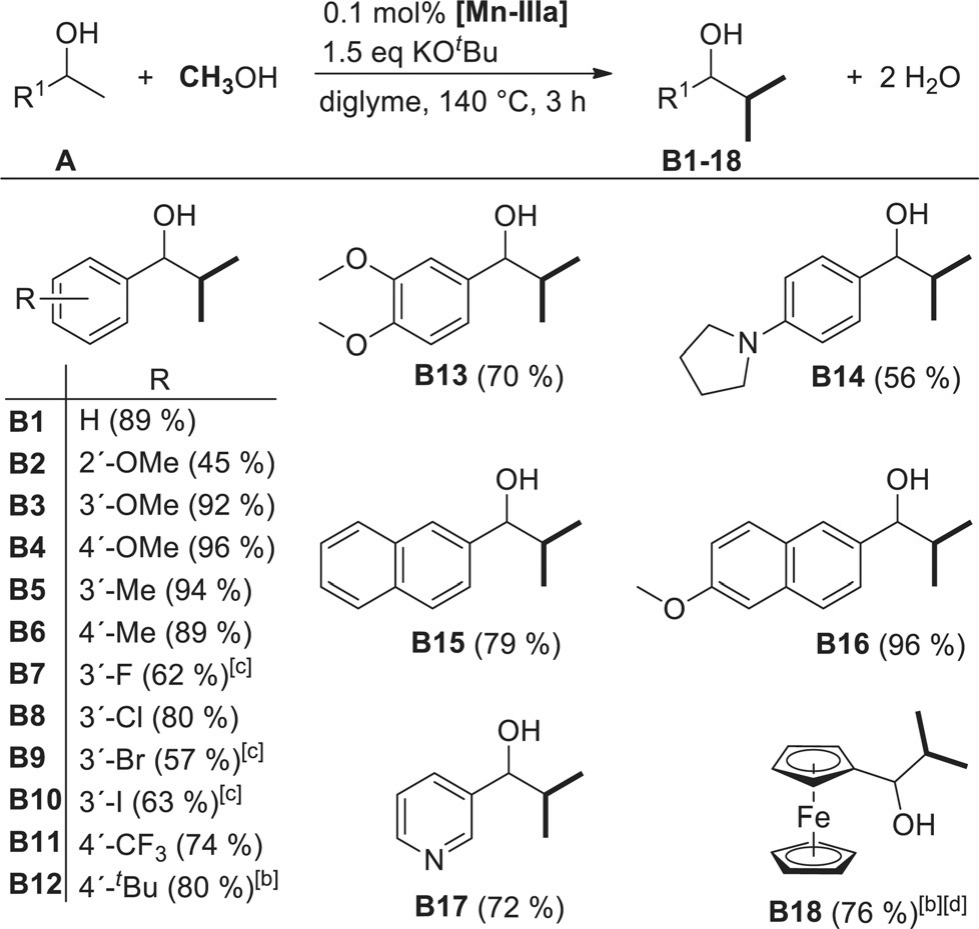

[a] Reaction conditions: 0.1 mol % **[Mn‐IIIa]** (1 μmol), 1.5 equiv KO^*t*^Bu (168 mg), **A** (1 mmol), MeOH (3 mmol, 122 μL), diglyme (2 mL), 140 °C (oil bath), 3 h. Isolated yields. [b] Yields determined by GC with *n*‐decane as internal standard. [c] 0.3 mol % **[Mn‐IIIa]**, 1.5 equiv NaO^*t*^Bu (144 mg). [d] 2 mol % **[Mn‐IIIa]**, 12 h.

After finding a broad substrate scope for the double methylation, we were interested in the mono methylation of secondary carbon atoms. We showed for three examples (**B19**–**B21**) that the methylation of secondary β‐carbon atoms (Table 3) is possible in similarly attractive yields and became interested in the methylation of more challenging substrates, which gave the products **B22**–**B24** in 65 to 91 % yields. After synthesizing the ephedrine derivative **B26** in 84 % yield, we became interested whether the secondary amine functionality found in ephedrine itself would also be tolerated. Fortunately, the catalyst system did indeed tolerate this functional group, leading to the synthesis of ephedrine (**B25**) from commercially available 2‐(methylamino)‐1‐phenylethan‐1‐ol in 80 % yield using 0.5 mol % catalyst loading. This experiment was easily scaled up to produce 5.97 g (76 %) of ephedrine **B25**. Furthermore, the catalyst is able to methylate a purely aliphatic amino alcohol (68 % **B27**). Eventually, we showcase three examples (**B28**–**B30**) including an aliphatic unsaturated one (**B30**) where the catalyst system methylates primary alcohols in very good to excellent yields.

**Table 3:** Substrate scope for the mono methylation of the secondary C‐atom.^[a]^

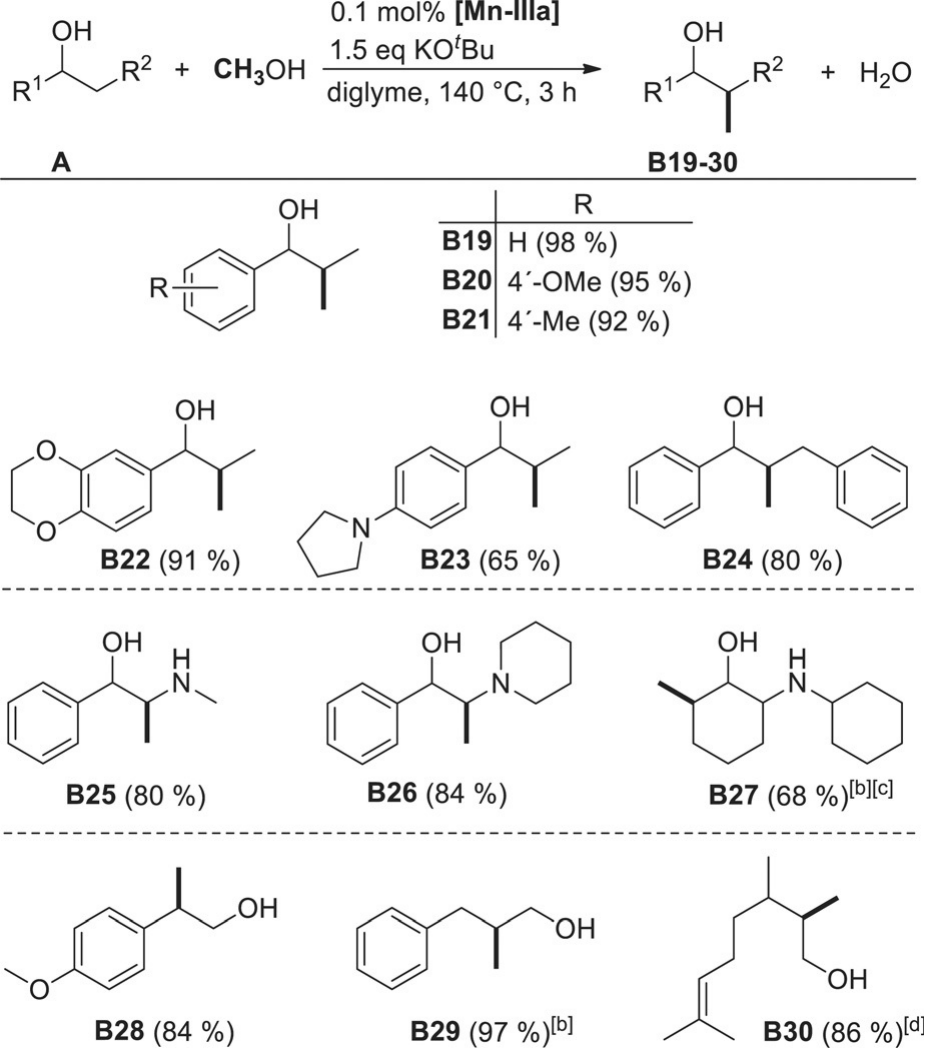

[a] Reaction conditions: 0.1 mol % **[Mn‐IIIa]** (1 μmol), 1.5 equiv KO^*t*^Bu (168 mg), **A** (1 mmol), MeOH (3 mmol, 122 μL), diglyme (2 mL), 140 °C (oil bath), 3 h. Isolated yields. [b] Yields determined by GC with *n*‐decane as internal standard. [c] 2 mol % **[Mn‐IIIa]**, 12 h. [d] 6 h.

Purely aliphatic 2‐octanol and alicyclic dodecanol were methylated in reasonable yields after increasing the catalyst loading, methanol amount, and reaction time, giving the corresponding products **B31** and **B32** in 73 % yield and 61 % yield, respectively (Scheme 1).

**Scheme 1:**
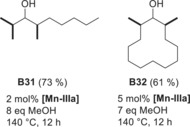
Examples for the multi methylation of secondary alcohols.

To gain insight into the reaction mechanism, a series of control experiments was conducted. In analogy to our previously published results,^22^ we postulate **[Mn‐IIIaH]K_2_** to be the active species in the hydride‐transfer step of the reaction sequence. To verify this assumption, we synthesized **[Mn‐IIIaH]K_2_** from **[Mn‐IIIaH]H_2_** by deprotonation with a potassium base (Scheme 2, experiment A). Next, we showed that the active species **[Mn‐IIIaH]K_2_** is formed under similar‐to‐catalysis conditions by comparing the ^31^P NMR spectra and the hydride signal in the ^1^H NMR spectra (experiment B). Using the in situ‐generated **[Mn‐IIIaH]K_2_**, we set out to investigate the reactivity towards unsaturated compounds. First, we investigated the stoichiometric reaction of **[Mn‐IIIaH]K_2_** with (*E*)‐chalcone. To our surprise, at room temperature using only 1 equiv of **[Mn‐IIIaH]K_2_** the C=C bond was quantitatively converted almost immediately, yielding 1,3‐diphenylpropan‐1‐one (experiment C). If we add 2 equiv of **[Mn‐IIIaH]K_2_**, both functionalities (olefin and ketone) become hydrogenated. 2 equiv of **[Mn‐IIIaH]H_2_** or **[Mn‐IIIaH]HK** do not transfer the hydride to the ketone and do not exist under catalytic conditions (excess of KO^*t*^Bu). Using the same conditions for the hydride transfer to *iso*‐butyrophenone (one observable intermediate of the reaction) yielded no product. The corresponding alcohol was obtained only after prolonged heating at 120 °C in 90 % yield (experiment D). Interestingly, there is nearly no decomposition of **[Mn‐IIIaH]K_2_** even within 24 h at 140 °C in the presence of an alcohol and base (catalysis relevant conditions). Note, we do the catalysis for between three and a maximum of 12 hours. The complex **[Mn‐IIIaH]K_2_** (no base and no alcohol, which can be dehydrogenated) decomposes by liberating H_2_ already at 50 °C. The hydride of **[Mn‐IIIaH]K_2_** and a proton (delivered by HO^*t*^Bu) form H_2_.^22^ We conclude that the catalyst is stable at 140 °C as long as it is kept busy by alcohol dehydrogenation and ketone hydrogenation, which means doing BH/HA.

**Scheme 2:**
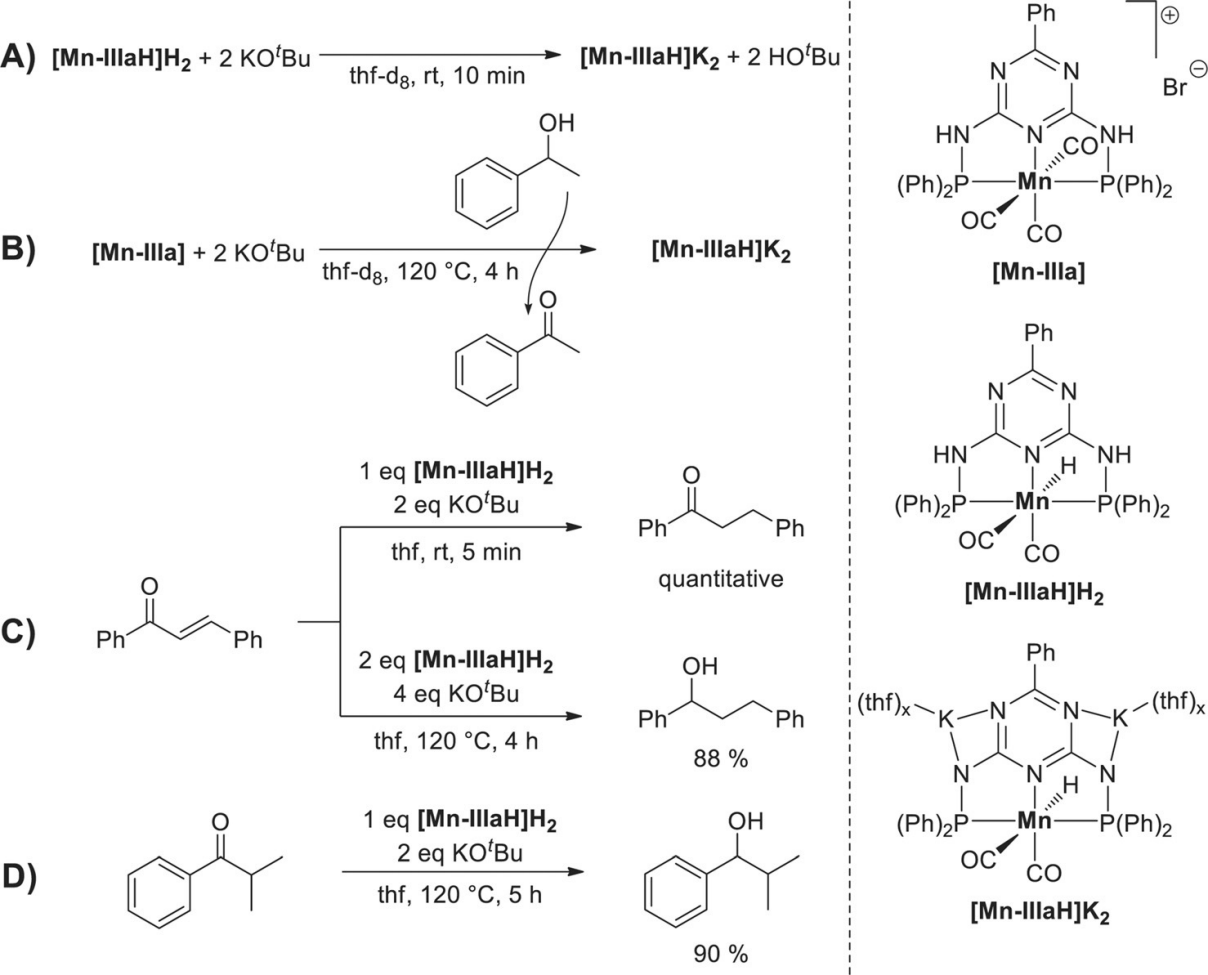
Control experiments to elucidate the mechanism of the reaction.

In summary, we report that an earth‐abundant catalyst permits the general methylation of alcohols by methanol. Our Mn‐based catalyst system permits the efficient single methylation of secondary carbon atoms and the double methylation of primary carbon atoms of primary and secondary alcohols, including purely aliphatic examples, and operates at low catalyst loadings (0.1 mol %) and short reaction times (3 h) at temperatures usually used for methanol‐based alkylation reactions. Many functional groups among them hydrogenation‐sensitive examples are tolerated, and upscaling is easily accomplished. Mechanistic investigations revealed that our novel bimetallic K‐Mn catalyst follows the borrowing hydrogen or hydrogen autotransfer mechanism. During the revision of our manuscript two related manuscripts appeared.^23^

## Conflict of interest

The authors declare no conflict of interest.

## Supporting information

As a service to our authors and readers, this journal provides supporting information supplied by the authors. Such materials are peer reviewed and may be re‐organized for online delivery, but are not copy‐edited or typeset. Technical support issues arising from supporting information (other than missing files) should be addressed to the authors.

Supplementary
