# Supplementary material for: Manganese‐Catalyzed β‐Methylation of Alcohols by Methanol
Source: Angew Chem Int Ed Engl. 2019 Dec 12;59(4):1485–90. doi: 10.1002/anie.201912055 (PMC7003965; doi:10.1002/anie.201912055)

Angewandte Chemie
Eine Zeitschrift der Gesellschaft Deutscher Chemiker

Supporting Information

Manganese-Catalyzed β-Methylation of Alcohols by Methanol

*Martin Schlagbauer, Fabian Kallmeier, Torsten Irrgang, and Rhett Kempe**

anie_201912055_sm_miscellaneous_information.pdf

Contents

General considerations 3
General procedures 4
Screening of the reaction parameters 6
Syntheses of the ligands 14
Syntheses of the precatalysts 14
Substrate scope 20
Mechanistic control experiments 33
References 40
Characterization data 41
  Characterization of complexes 41
  NMR-spectra of mechanistic control experiments 52
  NMR/MS-spectra 59
  Crystallographic data 87
  Checkcif report 88

General considerations

All reactions and manipulations with air or moisture sensitive compounds being present were performed under dry argon (Ar 5.0) or nitrogen (N_2 5.0), using Schlenk and glovebox techniques. Non-halogenated solvents were dried over sodium benzophenone or calcium hydride, halogenated solvents were dried over P_2O_5 and stored over molecular sieves (3 Å). Deuterated solvents were bought from Cambridge Isotope Laboratories, distilled accordingly, and stored over molecular sieves (3 Å). Other chemicals were purchased from commercial vendors and used without further purification if not noted otherwise. Alcohols that are not commercially available were obtained by reduction of the corresponding ketones with $LiAlH_4$ or $NaBH_4$ followed by purification via column chromatography or distillation. Hydrogenations were conducted in PARR Instrument stainless steel autoclaves N-MT5 300 mL equipped with heating mantles and temperature controllers. NMR spectra were collected on a Varian INOVA 300 (300 MHz for ^{1}H, 75 MHz for ^{13}C), Varian INOVA 400 (400 MHz for ^{1}H, 101 MHz for ^{13}C, 162 MHz for ^{31}P), or Bruker Avance III HD 500 (500 MHz for ^{1}H, 125.7 MHz for ^{13}C, 202 MHz for ^{31}P). Chemical shifts (δ) are reported in parts per million (ppm) relative to residual solvent signal. Coupling constants (J) are given in Hz (coupling patterns: s: singlet, d: doublet, t: triplet, q: quartet, m: multiplet). GC analyses were carried out using an Agilent Technologies 6890N system equipped with an Agilent HP-5 column (30 m, 320 µm, 0.25 µm) or an Agilent Technologies 6850 system equipped with a Macherey-Nagel (MN) Optima 17 column (30 m, 320 µm, 0.25 µm). GC/MS analyses were conducted on an Agilent 7890A GC system equipped with a HP 5MS column (30 m, 320 µm, 0.25 µm) and a 5975C inert MSD detector (EI, 70 eV). Gas mixtures were analyzed using an Agilent Technologies 6890N equipped with a TCD and an Agilent special PLOT and molsieve capillary column (30 m, 320 µm, 0.25 µm). FTIR measurements were carried out under a nitrogen atmosphere on an Agilent Cary 630 FTIR equipped with a Diamond ATR unit. Elemental analyses were performed using an Elementar Vario EL *III* or an Elementar Unicube. MN silica gel 60 (0.040– 0.063 mm particle size) was used for flash column chromatography. X-ray crystal structure analysis was performed with a STOE STADIVARI [λ(Mo-Kα) = 0.71073 Å] equipped with an Oxford Cryostream low temperature unit. Structure solution and refinement were accomplished with OlexSys 2[1], SHELXL-2014[2], WinGX[3] and Mercury 3.10.2[4].

General procedures

General Procedure 1 (GP1):

General procedure for the double methylation of primary β-carbon atoms of secondary alcohols:

In a glovebox, 1 mmol of educt alcohol, 1.5 eq KO^{t}Bu (1.5 mmol, 168 mg), 0.1 mol% precatalyst **[Mn-IIIa]** (1 µmol, 200 µL of a 5 µM stock solution in diglyme), 3 eq of methanol (3 mmol, 122 µL), and 1.8 mL diglyme were consecutively added to a pressure tube. The tube was sealed, taken outside of the glovebox, placed in a preheated oil bath at 140 °C and stirred for 3 h. Afterwards, the pressure tube was cooled to room temperature and the reaction was quenched by addition of water (1 mL). The aqueous layer was extracted using *tert*-butyl methyl ether (MTBE) or diethyl ether, the organic layers were combined, dried using Na_2SO_4 and the solvents removed *in vacuo*. The crude product was purified by column chromatography.

General Procedure 2 (GP2):

General procedure for the mono methylation of secondary β-carbon atoms of alcohols:

In a glovebox, 1 mmol of educt alcohol, 1.5 eq KO^{t}Bu (1.5 mmol, 168 mg), 0.1 mol% precatalyst **[Mn-IIIa]** (1 µmol, 200 µL of a 5 µM stock solution in diglyme), 3 eq of methanol (3 mmol, 122 µL), and 1.8 mL diglyme were consecutively added to a pressure tube. The tube was sealed, taken outside of the glovebox, placed in a preheated oil bath at 140 °C and stirred for 3 h. Afterwards, the pressure tube was cooled to room temperature and the reaction was quenched by addition of water (1 mL). The aqueous layer was extracted using MTBE or diethyl ether, the organic layers were combined, dried using Na_2SO_4 and the solvents removed *in vacuo*. The crude product was purified by column chromatography.

General Procedure 3 (GP3):

General procedure for the screening reaction:

In a glovebox, 1 mmol of 1-phenylethanol (121 µL), the chosen amount of base, precatalyst, methanol, and solvent were consecutively added to a pressure tube. The tube was sealed, taken outside of the glovebox, placed in a preheated oil bath and stirred for a selected time. Afterwards, the pressure tube was cooled to room temperature and the reaction was quenched by addition of water (1 mL). The organic layer was dried using Na_2SO_4 and analyzed by GC- and/or GCMS with decane as internal standard.

General Procedure 4 (GP4):

In a glovebox, 47.6 mmol of educt alcohol, 1.5 eq KO^{t}Bu (71.4 mmol, 8.011 g), 0.1 mol% precatalyst [Mn-IIIa] (37 mg) 3 eq of methanol (143 mmol, 5.77 mL), and 95 mL diglyme were consecutively added to a 2250 mL* Schlenk round-bottom flask. The flak was sealed tightly, taken outside of the glovebox, placed in a preheated oil bath at 140 °C and stirred for 3 h. After cooling the reaction mixture to room temperature, the pressure was carefully released and the reaction quenched by addition of water (50 mL). The aqueous layer was extracted using MTBE the organic layers were combined, dried using Na_2SO_4 and the solvents removed *in vacuo*. The crude product was purified by recrystallization or distillation. *: the ratio of the solvent volume to headspace volume must stay constant when upscaling.

Screening of the reaction parameters

The double methylation of 1-phenylethanol was chosen as model reaction for the optimization of the reaction parameters. Screening reactions were carried out according to GP3.

Table S1: Precatalyst screening[a]

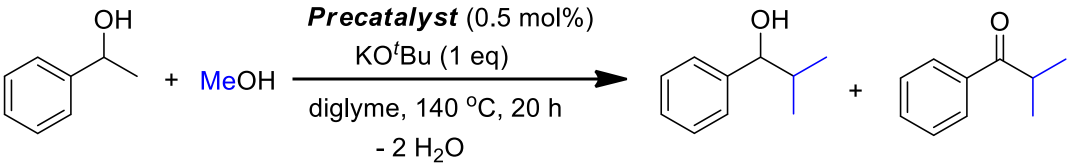

A1 **B1** **C1**

Entry	Precatalyst					Yield [%] B1	Yield [%] C1
1	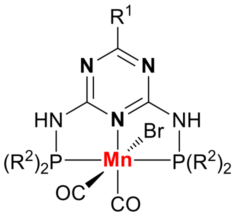	R^1 = H	R^2 = *i*Pr	**[Mn-I]**	(**a**)	45	9
2		R^1 = Me	R^2 = *i*Pr	**[Mn-II]**	(**a**)	47	10
3		R^1 = C_6H_5	R^2 = *i*Pr	**[Mn-III]**	(**a**)	54	9
4	(**a**)		R^2 = Ph	**[Mn-IIIa]**	(**b**)	63	10
5	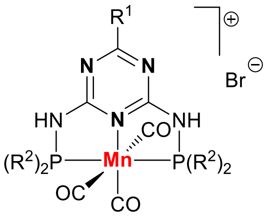		R^2 = Cy	**[Mn-IIIb]**	(**a**)	58	9
6		R^1 = 4-$CF_3(C_6H_4)$	R^2 = *i*Pr	**[Mn-IV]**	(**a**)	50	11
7		R^1 = NHC_3H_5	R^2 = *i*Pr	**[Mn-V]**	(**a**)	33	6
8	(**b**)	R^1 = NEt_2	R^2 = *i*Pr	**[Mn-VI]**	(**a**)	38	7
9	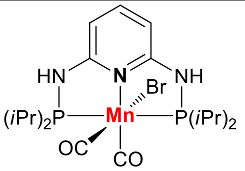			**[Mn-VII]**		26	4
10	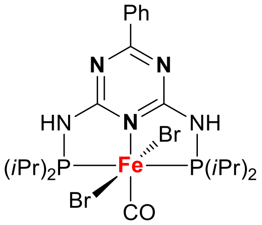			**[Fe-I]**		0	0
11	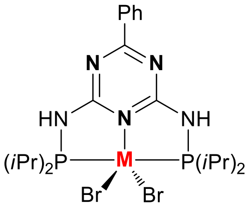	M = Mn		**[Mn-VIII]**		0	0
12		M = Fe		**[Fe-II]**		0	0
13		M = Co		**[Co-I]**		0	0

14	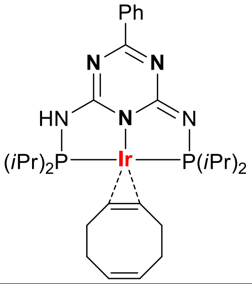	**[Ir-I]**	51	8
15	[$Mn(CO)_5Br$]		0	0
16	Precatalyst free		0	0

[a] Reaction conditions: 0.5 mol% precatalyst (0.005 mmol), KO^{t}Bu (1 mmol, 112.2 mg), **A1** (1 mmol, 121 µL), MeOH (3 mmol, 122 µL), diglyme (2 mL), 140 °C (oil bath), 20 h. Yields of **B1** and **C1** were determined by GC-analysis using *n*-decane as internal standard.

Table S2: Precatalyst **[Mn-IIIa]** loading screening[a]

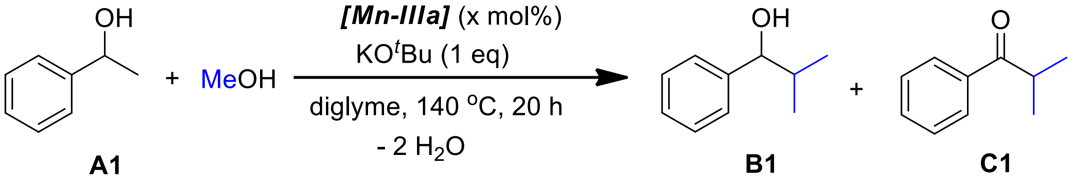

A1 **B1** **C1**

Entry	Precatalyst **[Mn-IIIa]** [mol%]	Yield **B1**	[%] **C1**
1	0.5	63	10
2	0.25	69	16
3	0.1	71	10
4	0.05	57	7
6	0.01	7	0

[a] Reaction conditions: **[Mn-IIIa]**, KO^{t}Bu (1 mmol, 112.2 mg), **A1** (1 mmol, 121 µL), MeOH (3 mmol, 122 µL), diglyme (2 mL), 140 °C (oil bath), 20 h. Yields of **B1** and **C1** were determined by GC-analysis using *n*-decane as internal standard.

Table S3: Solvent screening[a]

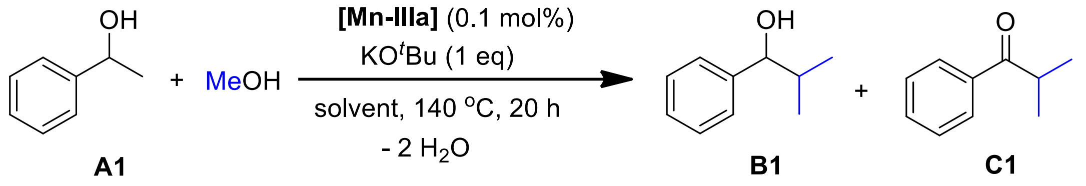

Entry	Solvent (2 mL)	Yield **B1**	[%] **C1**
1	decane	18	0
2	toluene	18	2
3	xylenes	28	2
4	dimethoxyethane	42	14
5	diethoxyethane	29	7
6	1-methoxy-2-(2-methoxyethoxy)ethane (diglyme)	71	10
7	thf	38	10
8	2Me-thf	32	4
9	2-methylbutan-2-ol	11	0
10	methanol	0	0
11	1,4-dioxane	19	0
12	*N*,*N*-dimethylformamide (DMF)	0	0
13	dimethyl sulfoxide (DMSO)	0	0
14	pyridine	14	0
15	methoxycyclopentane (CPME)	14	2

[a] Reaction conditions: 0.1 mol% **[Mn-IIIa]**, KO^{t}Bu (1 eq), **A1** (1 mmol, 121 µL), MeOH (3 mmol, 122 µL), solvent (2 mL), 140 °C (oil bath), 20 h. Yields of **B1** and **C1** were determined by GC-analysis using *n*-decane as internal standard.

Table S4: Base screening[a]

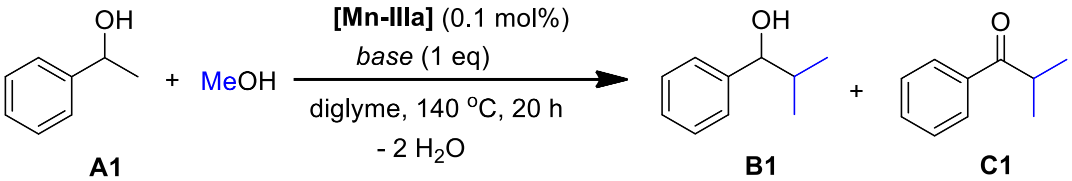

Entry	Base	Yield B1	[%] C1
1	LiOH	0	0
2	LiO^{t}Bu	0	0
3	LiN(TMS)$_2$	0	0
4	NaOH	6	0
5	NaO^{t}Bu	35	4
6	NaN(TMS)$_2$	0	0
7	KOH	19	0
8	KO^{t}Bu	71	10
9	KN(TMS)$_2$	66	9
10	KOAc	0	0
11	K_2CO_3	0	0
12	Cs_2CO_3	0	0

[a] Reaction conditions: 0.1 mol% **[Mn-IIIa]**, base (1 mmol, 112.2 mg), **A1** (1 mmol, 121 µL), MeOH (3 mmol, 122 µL), diglyme (2 mL), 140 °C (oil bath), 20 h. Yields of **B1** and **C1** were determined by GC-analysis using *n*-decane as internal standard.

Table S5: KO^{t}Bu loading screening[a]

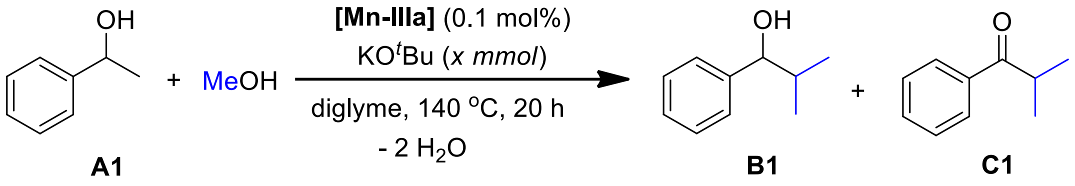

Entry	KO^{t}Bu [mmol]	Yield **B1**	[%] **C1**
1	0	0	0
2	0.1	10	3
3	0.5	42	15
4	0.8	52	9
5	1	71	10
6	1.2	74	9
7	1.5	90	9
8	1.7	73	10
9	2	73	5
10	2.2	56	0
11	2.5	52	0
12	3	25	0
13	4	3	0

[a] Reaction conditions: 0.1 mol% **[Mn-IIIa]**, KO^{t}Bu, **A1** (1 mmol, 121 µL), MeOH (3 mmol, 122 µL), diglyme (2 mL), 140 °C (oil bath), 20 h. Yields of **B1** and **C1** were determined by GC-analysis using *n*-decane as internal standard.

Table S6: Reaction temperature screening[a]

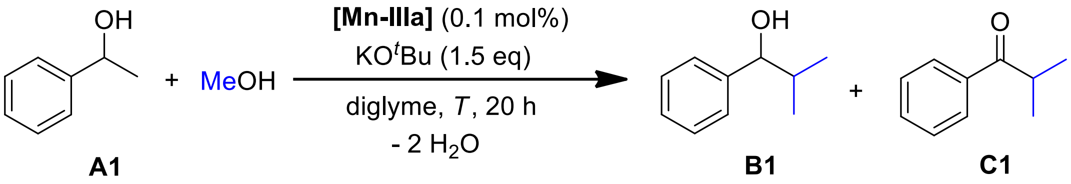

Entry	T [°C]	Yield **B1**	[%] **C1**
1	60	2	0
2	80	23	3
3	100	67	6
4	120	80	13
5	140	90	9
6	160	83	13

[a] Reaction conditions: 0.1 mol% **[Mn-IIIa]**, KO^{t}Bu (1.5 eq), **A1** (1 mmol, 121 µL), MeOH (3 mmol, 122 µL), diglyme (2 mL), *T* [°C] (oil bath), 20 h. Yields of **B1** and **C1** were determined by GC-analysis using *n*-decane as internal standard.

Table S7: Screening of the solvent (diglyme) quantity[a]

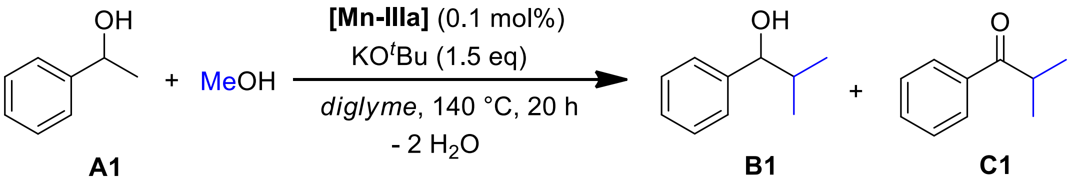

Entry	Diglyme [mL]	Yield **B1**	[%] **C1**
1	0.2	81	7
2	0.5	83	7
3	1	78	10
4	2	90	9
5	3	76	13
6	4	75	10

[a] Reaction conditions: 0.1 mol% **[Mn-IIIa]**, KO^{t}Bu (1.5 eq), **A1** (1 mmol, 121 µL), MeOH (3 mmol, 122 µL), *diglyme*, 140 °C (oil bath), 20 h. Yields of **B1** and **C1** were determined by GC-analysis using *n*-decane as internal standard.

Table S8: Screening of the methanol quantity[a]

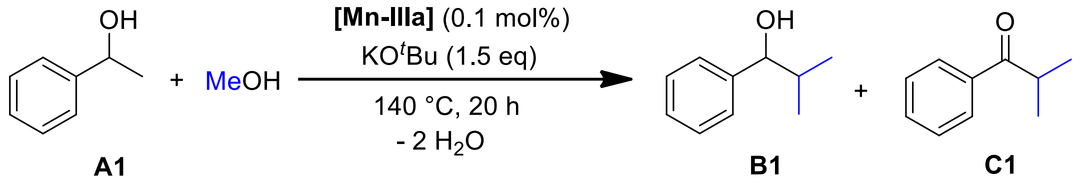

Entry	Diglyme [mL]	Methanol [mmol]	Yield B1	[%] C1
1	2	2	77	9
2	2	2.2	82	11
3	2	2.25	84	9
4	2	2.75	87	9
5	2	3	90	9
6	2	3.5	88	9
7	2	4	87	9
8	2	5	89	10
9	2	8	69	4
10	no	52.4	0	0

[a] Reaction conditions: 0.1 mol% **[Mn-IIIa]**, KO^{t}Bu (1.5 eq), **A1** (1 mmol, 121 µL), *MeOH*, 140 °C (oil bath), 20 h. Yields of **B1** and **C1** were determined by GC-analysis using *n*-decane as internal standard.

Time-Conversion Analysis

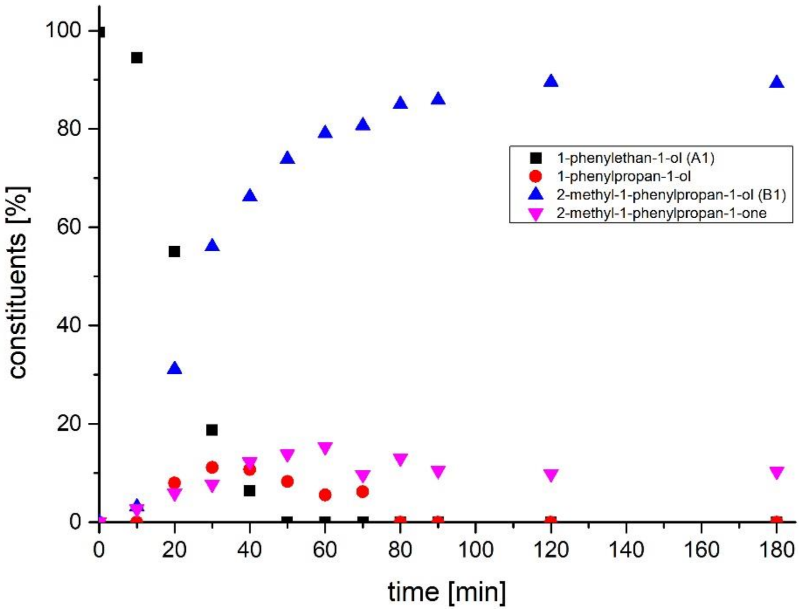

Figure S1: Time-conversion plot of the reaction.

After optimization of the reaction conditions a time-conversion study was carried out.

In a glovebox, 12 mmol of 1-phenylethanol, 1.5 eq KO^{t}Bu (18 mmol, 2020 mg), 0.1 mol% precatalyst [Mn-IIIa] (1 µmol, 2400 µL of a 5 µM stock solution in diglyme), 3 eq of methanol (36 mmol,1460 µL), and 21.6 mL diglyme were consecutively added to a 50 mL round-bottom flask and stirred for 5 min (1200 µL decane was added as internal standard). The reaction mixture was evenly divided between 12 pressure tubes, the tubes were sealed, taken outside of the glovebox, placed in a heated oil bath at 140 °C. After the chosen amount of time one pressure tube was removed from the oil bath and quickly cooled down by placing it into an ice bath, quenched by addition of water (1 mL), and extracted with diethyl ether. The organic layer was dried using Na_2SO_4 and analyzed by GC.

Syntheses of the ligands

The ligands N^2,N^4-bis(diisopropylphosphanyl)-1,3,5-triazine-2,4-diamine[5], N^2,N^4-bis(diisopropyl-phosphanyl)-6-methyl-1,3,5-triazine-2,4-diamine[5], N^2,N^4-bis(diisopropylphosphanyl)-6-phenyl-1,3,5-triazine-2,4-diamine[5], N^2-cyclopropyl-N^4,N^6-bis(diisopropylphosphanyl)-1,3,5-triazine-2,4,6-triamine[6], N^2,N^4-bis(diisopropylphosphanyl)-N^6,N^6-diethyl-1,3,5-triazine-2,4,6-triamine[7], N^2,N^4-bis(diisopropylphosphanyl)-6-(4-(trifluoromethyl)phenyl)-1,3,5-triazine-2,4-diamine[8], N^2,N^6-bis(diisopropylphosphanyl)pyridine-2,6-diamine[5] were prepared according to literature procedures.

The ligands N^2,N^4-bis(diphenylphosphanyl)-6-phenyl-1,3,5-triazine-2,4-diamine and N^2,N^4-bis(dicyclohexylphosphanyl)-6-phenyl-1,3,5-triazine-2,4-diamine were synthesized following a modified procedure of the mentioned ligand syntheses, *vide supra*.

Syntheses of the precatalysts

The precatalysts [Mn-I][9], [Mn-II][7], [Mn-III][7], [Mn-IV][10], [Mn-V][7], [Mn-VI][7],[Mn-VII][11], [Fe-I][12],[Fe-II][12],[Ir-I][5], were prepared according to literature. The precatalysts [Mn-VIII][7] and [Co-I][6] were prepared analogous to already published protocols of our group.

Preparation of [Mn-IIIa]:

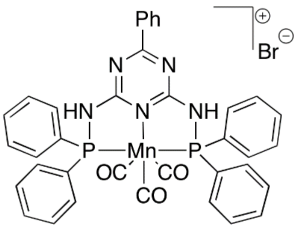

In a glovebox, 1 mmol [$Mn(CO)_5Br$] (275 mg), 1 mmol N^2,N^4-bis(diphenylphosphanyl)-6-phenyl-1,3,5-triazine-2,4-diamine (556 mg) were placed in a 100 mL Schlenk-tube and dissolved in 15 mL of THF. The tube was sealed, taken outside of the glovebox and a reflux condenser with a bubble counter was attached under argon. The reaction was heated to reflux (oil bath 90 °C) and stirred for 3 h. After the suspension cooled down to room temperature, precipitation was completed by adding 20 mL n-hexane. The precipitate was filtered off, the orange solid residue was washed two times with n-hexane and dried *in vacuo*. Crystals, suitable for X-Ray single crystal analysis were grown by evaporation of a saturated solution of **[Mn-IIIa]** in diglyme under nitrogen.

Yield: [**Mn-IIIa**]: 89 % (0.901 mmol, 698 mg) as an orange solid.

^{1}H NMR (500 MHz, DMSO-d$_6$): δ = 10.97 (s, 2H), 8.49 – 8.38 (m, 2H), 7.96 (d, *J* = 5.2 Hz, 4H), 7.72 – 7.57 (m, 7H), 7.52 – 7.41 (m, 10H), 7.38 (t, *J* = 7.3 Hz, 2H) ppm.

^{13}C NMR (126 MHz, DMSO-d$_6$): δ = 170.76, 170.64, 170.52, 170.42, 138.66, 138.48, 138.29, 135.14, 134.73, 134.55, 132.78, 132.14, 132.10, 130.23, 129.91, 129.55, 129.50, 128.78, 128.51, 128.46, 127.88 ppm.

^{31}P NMR (202 MHz, DMSO-d$_6$): δ = 113.5 ppm.

Elemental analysis calcd for $C_{36}H_{27}BrMnN_5O_3P_2$ (M: 774.43 g/mol) [%]: C 55.83, H 3.51, N 9.04, found: C 56.00, H 3.97, N 9.12.

IR: (C=O): 1939.32 cm^{-1}, (C=O): 1992.60 cm^{-1}, (C=O): 1835,15 cm^{-1} (see spectra at S42).

Preperation of [Mn-IIIb]:

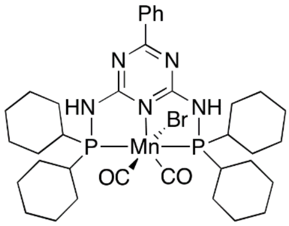

In a glovebox, 1 mmol [Mn(CO)$_5$Br] (275 mg) and 1 mmol *N*^{2},*N*^{4}-bis(dicyclohexylphosphanyl)-6-phenyl-1,3,5-triazine-2,4-diamine (580 mg) were placed in a 50 mL Schlenk tube and dissolved in 30 mL of dry thf. The tube was sealed, taken outside of the glovebox and a reflux condenser with a bubble counter was attached under argon. The reaction was heated to reflux (oil bath 90 °C) and stirred for 3 h. Afterwards the hot solution was filtered through a metal cannula equipped with a glass microfiber filter and the solvent removed *in vacuo*. The orange residue was scraped off the glass, suspended in 20 mL dry n-hexane and refluxed for 5 min. The hot hexanes were filtered off and the residue dried *in vacuo*. Crystals, suitable for X-Ray single crystal analysis were grown by slowly cooling a hot, saturated solution of **[Mn-IIIb]** in thf to room temperature.

Yield: [**Mn-IIIb**]: 95 % (0.951 mmol, 732 mg) as an orange solid.

^{1}H NMR (500 MHz, thf-d$_8$): δ = 8.39 – 8.26 (m, 4H), 7.48 (t, *J* = 6.5 Hz, 1H), 7.41 (t, *J* = 7.1 Hz, 2H), 3.28 (s, 2H), 2.60 – 2.39 (m, 4H), 2.19 – 2.01 (m, 4H), 1.97 – 1.76 (m, 16H), 1.62 – 1.42 (m, 6H), 1.40 – 1.25 (m, 12H) ppm.

^{13}C NMR (126 MHz, thf-d$_8$): δ = 172.04, 171.93, 171.83, 171.14, 137.29, 132.84, 129.40, 129.08, 39.83, 39.75, 39.67, 39.64, 39.56, 30.27, 29.27, 28.77, 28.57, 28.51, 28.45, 28.28, 28.24, 28.20, 28.15, 28.12, 28.09, 27.97, 27.92, 27.90, 27.79, 27.45, 26.96, 26.55 ppm.

^{31}P NMR (202 MHz, thf-d$_8$): δ = 126.1 ppm.

Elemental analysis calcd for $C_{35}H_{51}BrMnN_5O_2P_2$ (M: 770.62 g/mol) [%]: C 54.55, H 6.67, N 9.09, found: C 54.53, H 7.06, N 9.05.

IR: (C=O): 1928.43 cm^{-1}, (C=O): 1845.75 cm^{-1} (see spectra at S44).

Preperation of [Mn-IIIaH]H_2:

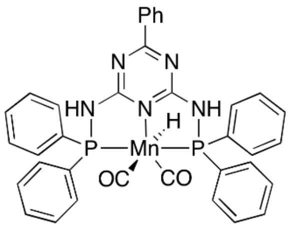

In a glovebox, 1 mmol **[Mn-IIIa]** (774 mg) and 1 mmol KO^{t}Bu (112 mg) were placed in a glass insert of a 300 mL autoclave and dissolved in 20 mL of dry thf. The autoclave was sealed, taken outside of the glovebox and purged three times with hydrogen, before adjusting the pressure to 30 bar. The reaction was heated to 30 °C and stirred for 24 h. After cooling to room temperature the pressure was carefully released, the autoclave purged with nitrogen and taken inside a glovebox. The suspension was filtered, the red solution transferred to a Schlenk-tube and the solvent was removed *in vacuo*. The red residue was recrystallized from a thf/hexane mixture at -20 °C.

Yield **[Mn-IIIaH]H_2**: 70% (467 mg) as a red solid.

^{1}H NMR (500 MHz, thf-d_8): δ = 9.29 (s, 2H), 8.29 (d, *J* = 7.5 Hz, 2H), 7.94 (d, *J* = 5.8 Hz, 4H), 7.70 (d, *J* = 5.8 Hz, 4H), 7.36 (dt, *J* = 12.0, 6.9 Hz, 15H), -5.08 (t, *J* = 50.6 Hz, 1H) ppm.

^{13}C NMR (126 MHz, thf-d_8): δ = 170.00, 169.87, 169.74, 168.22, 141.09, 140.92, 140.78, 140.74, 140.62, 140.46, 136.37, 132.43, 132.37, 132.32, 131.43, 130.43, 130.38, 130.33, 129.67, 129.07, 128.07, 128.00, 127.86, 127.82, 127.78, 127.73, 127.69, 127.65, 124.94 ppm.

^{31}P NMR (202 MHz, thf-d_8): δ = 133.83 ppm.

Elemental analysis calcd for $C_{35}H_{28}MnN_5O_2P_2$(1x thf (C_4H_8O) (M: 667.53 g/mol) [%]: C 63.33, H 4.91, N 9.47, found: C 63.51, H 5.01, N 9.40.

IR: (C=O): 1914.43 cm^{-1}, (C=O): 1851.875 cm^{-1} (see spectra at S46).

Hydrogen elimination in solution

^{1}H and ^{31}P NMR experiments indicate that the complex **[Mn-IIIaH]H₂** eliminates hydrogen in solution, see NMR spectra and figure S2 below, respectively.

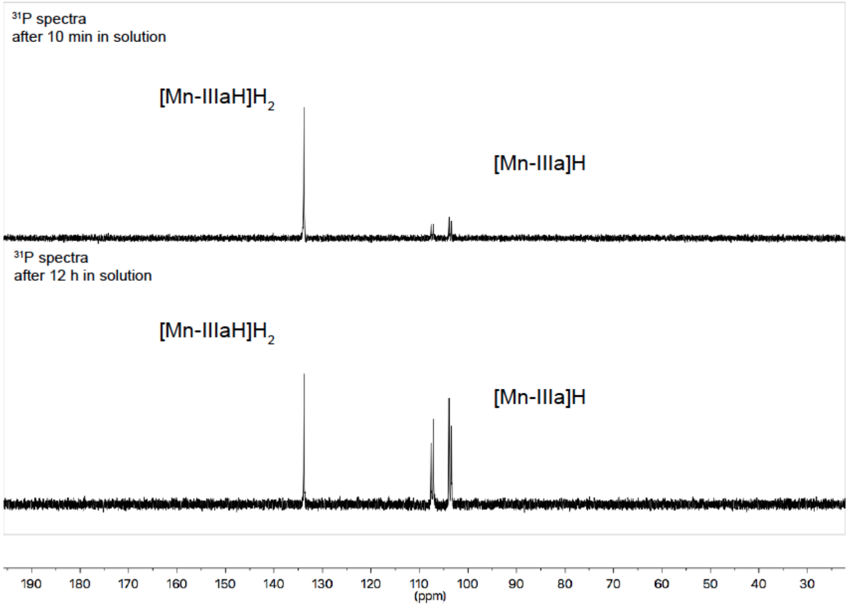

Figure S2: ^{31}P analytic of **[Mn-IIIaH]H_2**, top: ^{31}P spectra directly after dissolving the complex. Bottom: ^{31}P spectra 12 hours after dissolving the complex.

In a glovebox with nitrogen atmosphere, 150 mg of **[Mn-IIIaH]H_2** were placed in a 5 mL Schlenk-tube and dissolved in 4 mL dry thf, sealed with a septum and taken outside of the glovebox. After 3 h at room temperature the gas mixture in the headspace of the solution were analyzed by gas-chromatography. The result of this experiment verifies that hydrogen is eliminated from **[Mn-IIIaH]H_2**. (see figure S3 below)

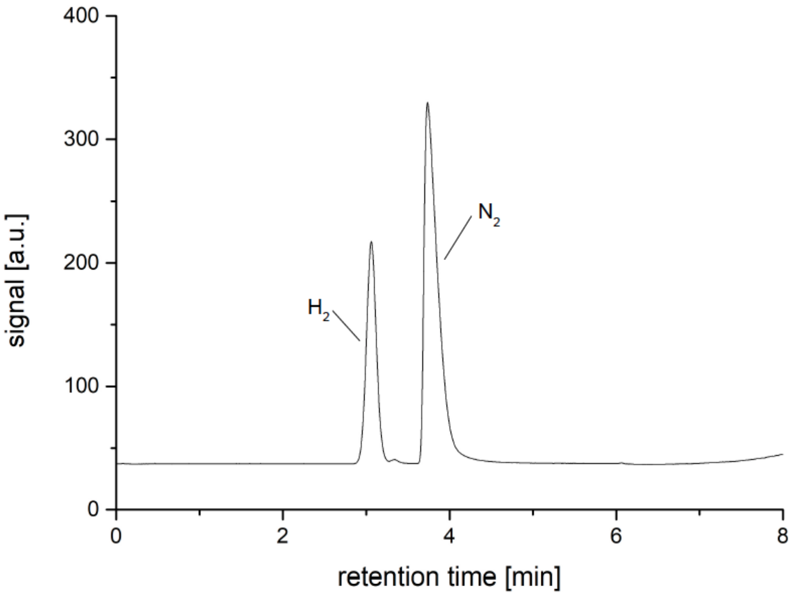

Figure S3: Gas-chromatogram of the gas mixture in the headspace over a solution of **[Mn-IIIaH]H_2** in thf after 3hours.

Preperation of [Mn-IIIaH]K_2 with KO^{t}Bu:

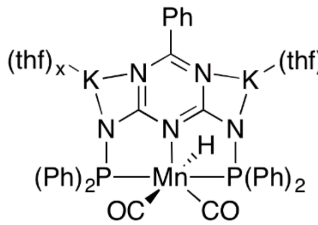

In a glovebox, 37.5 µmol **[Mn-IIIaH]H_2** (25 mg) were placed in a Young NMR tube, dissolved with 200 µL thf-d_8 and 2 eq KO^{t}Bu (74.9 µmol) as 500 µL of a 14.6 µM stock solution in thf-d_8 were added. The mixture was analyzed by ^{1}H and ^{31}P NMR. NMR analysis indicates that the double deprotonated hydride species **[Mn-IIIaH]K_2** is formed. The species **[Mn-IIIa]K** is formed as by-product due to hydrogen elimination.

In a glovebox, 150 µmol **[Mn-IIIaH]H_2** (100 mg) were placed in a Schlenk tube, dissolved with 1000 µL thf and 2 eq KO^{t}Bu (300 µmol) were added. The tube was sealed, taken outside the glovebox and purged with hydrogen. The mixture was heated to 50 °C for 30 min, cooled to -78 °C and dried *in vacuo*

^{1}H NMR (400 MHz, thf-d_8): δ = 8.23 – 8.04 (m, 2H), 8.05 – 7.92 (m, 4H), 7.88 – 7.72 (m, 4H), 7.25 – 7.18 (m, 3H), 7.05 (q, *J* = 6.8, 6.4 Hz, 8H), 7.00 – 6.95 (m, 2H), 6.90 (t, *J* = 7.4 Hz, 2H), -4.57 (t, *J* = 47.7 Hz, 1H) ppm.
^{13}C NMR (75 MHz, thf-d_8): δ = 176.78, 176.56, 176.35, 151.73, 151.56, 151.46, 151.32, 151.21, 151.08, 143.80, 134.06, 133.98, 133.90, 132.71, 132.64, 132.56, 129.91, 129.21, 129.18, 128.47, 128.42, 128.37, 128.27, 128.22, 128.16, 128.11, 127.77 ppm.

^{31}P NMR (162 MHz, thf-d_8): δ = 128.63 ppm.

Elemental analysis calcd for $C_{35}H_{26}K_2MnN_5O_2P_2$(1x thf ($C_4H_8O$) (M: 743.71 g/mol) [%]: C 57.42, H 4.20, N 8.58, found: C 57.59, H 4.38, N 8.74.

IR: (C=O): 1944.00 cm^{-1}, (C=O): 1840,84 cm^{-1} (see spectra at S48).

Preperation of [Mn-IIIaH]K_2 with KH:

In a glovebox, 37.5 µmol **[MnIIIaH]H_2** (25 mg) were placed in a Young NMR tube, dissolved with 200 µL thf-d_8 and 2 eq KH (74.9 µmol, as suspension in 400 µL thf-d_8) were added. The mixture was analyzed by ^{1}H and ^{31}P NMR. The double deprotonated hydride species **[Mn-IIIaH]K_2** is formed. The species **[Mn-IIIa]K** is formed as by-product due to hydrogen elimination.

In a glovebox, 150 µmol **[Mn-IIIaH]H$_2$** (100 mg) were placed in a Schlenk tube, dissolved with 1000 µL thf and 2 eq KH (300 µmol) were added. The tube was sealed taken, outside the glovebox and purged with hydrogen. The mixture was heated to 50 °C for 30 min, cooled to -78 °C and dried *in vacuo*

^{1}H NMR (500 MHz, thf-d$_8$): δ = -4,47 (t, *J* = 47.7 Hz, 1H) ppm.

^{31}P NMR (202 MHz, thf-d$_8$): δ = 128.3 ppm.

Preperation of [Mn-IIIa]K:

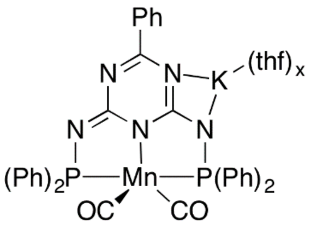

In a glovebox, 25.8 µmol **[Mn-IIIa]** (10 mg) was dissolved with 200 µL thf-d$_8$ and 2 eq KO^{t}Bu (51.6 µmol) as 500 µL of a 10.3 µM stock solution in thf-d$_8$ was added, the solution was filtered and placed in a Young NMR tube. The mixture was analyzed by ^{31}P NMR.

^{31}P NMR (202 MHz, thf-d$_8$): δ = 105.3 ppm.

Substrate scope

The starting materials 1-(4-(pyrrolidin-1-yl)phenyl)ethan-1-ol[13], 1-(4-(pyrrolidin-1-yl)phenyl)propan-1-ol[13],1-phenyl-2-(piperidin-1-yl)ethan-1-ol[14] were prepared according to literature.

2-methyl-1-phenylpropan-1-ol (B1 / B19)

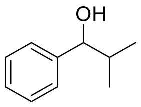

Molecular Weight: 150,22

Synthesised starting from 1-phenylethan-1-ol following GP1.

Purification by column chromatography (pentane 90 / 10 diethyl ether). Yield: 89 % (0.892 mmol, 134 mg) as a colourless oil.

Synthesised starting from 1-phenylpropan-1-ol following GP2:

Purification by column chromatography (pentane 90 / 10 diethyl ether). Yield: 98 % (0.979 mmol, 147 mg) as a colourless oil.

^{1}H NMR (400 MHz, $CDCl_3$): δ = 7.35 – 7.18 (m, 1H), 4.29 (d, *J* = 6.8 Hz, 1H), 2.23 (s, 1H), 1.91 (dq, *J* = 13.5, 6.8 Hz, 1H) ppm.

^{13}C NMR (126 MHz, $CDCl_3$): δ = 143.73, 128.17, 127.38, 126.66, 79.99, 35.26, 19.01, 18.37 ppm.

1-(2-methoxyphenyl)-2-methylpropan-1-ol (B2)

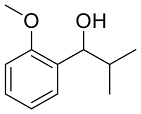

Molecular Weight: 180,25

Synthesised starting from 1-(2-methoxyphenyl)ethan-1-ol following GP1.

Purification by column chromatography (pentane 88 / 12 MTBE). Yield: 45 % (0.455 mmol, 82 mg) as a colourless oil.

^{1}H NMR (500 MHz, CD_2Cl_2): δ = 7.28 – 7.20 (m, 2H), 6.96 – 6.91 (m, 1H), 6.90 (d, *J* = 8.0 Hz, 1H), 4.48 (t, *J* = 7.1 Hz, 1H), 3.82 (s, 3H), 2.44 (d, *J* = 7.0 Hz, 1H), 2.08 – 1.98 (m, 1H), 0.98 (d, *J* = 6.7 Hz, 3H), 0.78 (d, *J* = 6.8 Hz, 3H) ppm.

^{13}C NMR (126 MHz, CD_2Cl_2): δ = 157.30, 132.41, 128.54, 128.52, 120.87, 111.08, 77.10, 55.72, 34.65, 19.89, 18.63 ppm.

1-(3-methoxyphenyl)-2-methylpropan-1-ol (B3)

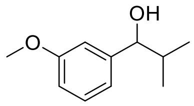

Molecular Weight: 180,25

Synthesised starting from 1-(3-methoxyphenyl)ethan-1-ol following GP1.

Purification by column chromatography (pentane 88 / 12 MTBE). Yield: 92 % (0.915 mmol, 165 mg) as a colourless oil.

^{1}H NMR (500 MHz, CD_2Cl_2): δ = 7.24 (t, *J* = 7.8 Hz, 1H), 6.91 – 6.84 (m, 2H), 6.83 – 6.77 (m, 1H), 4.31 (d, *J* = 6.7 Hz, 1H), 3.79 (s, 3H), 1.99 (s, 1H), 1.97 – 1.86 (m, 1H), 0.97 (d, *J* = 6.7 Hz, 3H), 0.80 (d, *J* = 6.8 Hz, 3H) ppm.

^{13}C NMR (126 MHz, CD_2Cl_2): δ = 160.14, 146.28, 129.56, 119.41, 113.08, 112.58, 80.19, 55.66, 35.83, 19.40, 18.39 ppm.

1-(4-methoxyphenyl)-2-methylpropan-1-ol (B4 / B20)

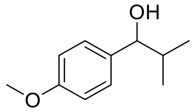

Molecular Weight: 180,25

Synthesised starting from 1-(4-methoxyphenyl)ethan-1-ol following GP1.

Purification by column chromatography (pentane 88 / 12 MTBE). Yield: 96 % (0.960 mmol, 175 mg) as a colourless oil.

Synthesised starting from 1-(4-methoxyphenyl)propan-1-ol following GP2.

Purification by column chromatography (pentane 88 / 12 MTBE). Yield: 95 % (0.949 mmol, 171 mg) as a colourless oil.

Upscaling starting from 1-(4-methoxyphenyl)ethan-1-ol following GP 4.

Purification by distillation (bp 75 °C, $2*10^{-2}$ mbar). Yield: 87 % (41.3 mmol, 7.46 g) as a colourless oil.

^{1}H NMR (500 MHz, CD_2Cl_2): δ = 7.22 (d, *J* = 8.6 Hz, 2H), 6.86 (d, *J* = 8.7 Hz, 2H), 4.27 (dd, *J* = 6.9, 2.8 Hz, 1H), 3.78 (s, 3H), 1.94 – 1.78 (m, 2H), 0.97 (d, *J* = 6.7 Hz, 3H), 0.75 (d, *J* = 6.8 Hz, 3H) ppm.

^{13}C NMR (126 MHz, CD_2Cl_2): δ = 159.47, 136.62, 128.20, 113.92, 79.98, 55.72, 35.91, 19.27, 18.68 ppm.

2-methyl-1-(*m*-tolyl)propan-1-ol (B5)

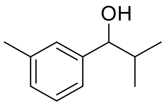

Molecular Weight: 164,25

Synthesised starting from 1-(*m*-tolyl)ethan-1-ol following GP1.

Purification by column chromatography (pentane 90 / 10 MTBE). Yield: 94 % (0.938 mmol, 154 mg) as a colourless oil.

^{1}H NMR (500 MHz, CD_2Cl_2): δ = 7.22 (t, *J* = 7.5 Hz, 1H), 7.13 (s, 1H), 7.09 (d, *J* = 7.4 Hz, 2H), 4.29 (dd, *J* = 6.8, 2.2 Hz, 1H), 2.35 (s, 3H), 2.00 – 1.86 (m, 2H), 0.98 (d, *J* = 6.7 Hz, 3H), 0.79 (d, *J* = 6.8 Hz, 3H) ppm.

^{13}C NMR (126 MHz, CD_2Cl_2): δ = 144.50, 138.32, 128.49, 128.47, 127.77, 124.17, 80.36, 35.82, 21.74, 19.41, 18.50 ppm.

2-methyl-1-(*p*-tolyl)propan-1-ol (B6 / B21)

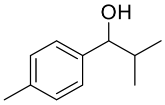

Molecular Weight: 164,25

Synthesised starting from 1-(*p*-tolyl)ethan-1-ol following GP1.

Purification by column chromatography (pentane 98 / 2 ethyl acetate). Yield: 89 % (0.888 mmol, 146 mg) as a colourless oil.

Synthesised starting from 1-(*m*-tolyl)propan-1-ol following GP2:

Purification by column chromatography (pentane 98 / 2 ethyl acetate). Yield: 92 % (0.919 mmol, 151 mg) as a colourless oil.

^{1}H NMR (500 MHz, $CDCl_3$): δ = 7.10 (d, *J* = 8.1 Hz, 2H), 7.05 (d, *J* = 7.9 Hz, 2H), 4.21 (d, *J* = 7.0 Hz, 1H), 2.26 (s, 3H), 1.85 (dq, *J* = 13.5, 6.8 Hz, 2H), 0.91 (d, *J* = 6.7 Hz, 3H), 0.69 (d, *J* = 6.8 Hz, 3H) ppm.

^{13}C NMR (126 MHz, $CDCl_3$): δ = 140.80, 137.10, 128.96, 126.62, 80.03, 35.29, 21.22, 19.10, 18.50 ppm.

1-(3-fluorophenyl)-2-methylpropan-1-ol (B7)

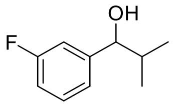

Molecular Weight: 168,21

Synthesised starting from 1-(3-fluorophenyl)ethan-1-ol following GP1.

Precatalyst loading has been raised 0.3 mol% and NaO^tBu was used (see also synthesis of B9).

Purification by column chromatography (pentane 90 / 10 diethyl ether). Yield: 62 % (0.624 mmol, 105 mg) as a colourless oil.

^{1}H NMR (500 MHz, CD_2Cl_2): δ = 7.30 (td, *J* = 7.9, 6.0 Hz, 1H), 7.30 (td, *J* = 7.9, 6.0 Hz, 1H), 7.09 (d, *J* = 7.7 Hz, 1H), 7.07 – 7.03 (m, 1H), 6.99 – 6.93 (m, 1H), 4.39 (dd, *J* = 6.4, 3.4 Hz, 1H), 1.97 – 1.86 (m, 2H), 0.94 (d, *J* = 6.7 Hz, 3H), 0.82 (d, *J* = 6.8 Hz, 3H) ppm.

^{13}C NMR (126 MHz, CD_2Cl_2): δ = 164.32, 162.37, 147.34, 147.29, 130.13, 130.07, 122.81, 122.79, 114.50, 114.33, 113.88, 113.71, 79.47, 79.46, 35.87, 19.24, 18.04 ppm.

1-(3-chlorophenyl)-2-methylpropan-1-ol (B8)

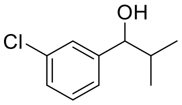

Molecular Weight: 184,66

Synthesised starting from 1-(3-chlorophenyl)ethan-1-ol following GP1.

Purification by column chromatography (pentane 85 / 15 MTBE). Yield: 80 % (0.804 mmol, 148 mg) as a colourless oil.

^{1}H NMR (500 MHz, CD_2Cl_2): δ = 7.35 – 7.17 (m, 4H), 4.37 (dd, *J* = 6.4, 3.4 Hz, 1H), 1.97 (d, *J* = 3.5 Hz, 1H), 1.90 (dp, *J* = 13.4, 6.7 Hz, 1H), 0.94 (d, *J* = 6.7 Hz, 3H), 0.81 (d, *J* = 6.8 Hz, 3H) ppm.

^{13}C NMR (126 MHz, CD_2Cl_2): δ = 146.70, 134.42, 129.96, 127.78, 127.13, 125.39, 79.47, 35.86, 19.24, 18.04 ppm.

1-(3-bromophenyl)-2-methylpropan-1-ol (B9)

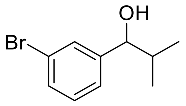

Molecular Weight: 229,12

Synthesised starting from 1-(3-bromophenyl)ethan-1-ol following GP1.

In order to avoid dehalogenation NaO^{t}Bu was used. To compensate the lowered activity, the precatalyst loading had to be adjusted. A small precatalyst-loading screening was carried out for the double methylation of 1-(3-bromophenyl)ethan-1-ol.

Table S9: Screening of the precatalyst loading when NaO^{t}Bu is used. [a]

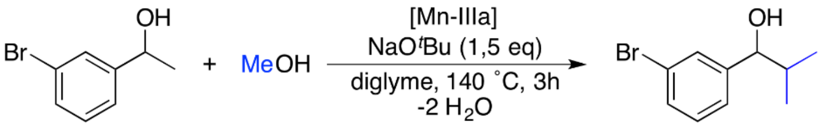

Precatalyst loading [**Mn-IIIa**][mol%]	Yield 1-(3-bromophenyl)-2-methylpropan-1-ol [%]
0.1	57
0.3	66
0.5	63
0.8	64

[a] Reaction conditions: **[Mn-IIIa]**, NaO^{t}Bu (1.5 eq), 1-(3-bromophenyl)ethan-1-ol (1 mmol), MeOH (3 mmol, 122 µL), *diglyme*, 140 °C (oil bath), 3 h. Yields of were determined by GC-analysis using *n*-decane as internal standard.

The Reaction was carried out with a precatalyst loading of 0.3 mol% [Mn-IIIa] and 1.5 eq NaO^{t}Bu was used.

Purification by column chromatography (pentane 88 / 12 diethyl ether). Yield: 57 % (0.572 mmol, 131 mg) as a colourless oil.

^{1}H NMR (500 MHz, CD_2Cl_2): δ = 7.50 – 7.47 (m, 1H), 7.40 (dt, *J* = 7.4, 1.7 Hz, 1H), 7.27 – 7.19 (m, 2H), 4.36 (dd, *J* = 6.4, 3.3 Hz, 1H), 1.95 (d, *J* = 3.5 Hz, 1H), 1.90 (dq, *J* = 13.4, 6.7 Hz, 1H), 0.94 (d, *J* = 6.7 Hz, 3H), 0.81 (d, *J* = 6.8 Hz, 3H) ppm.

^{13}C NMR (126 MHz, CD_2Cl_2): δ = 146.97, 130.74, 130.27, 130.07, 125.85, 122.71, 79.42, 35.87, 19.24, 18.03 ppm.

1-(3-iodophenyl)-2-methylpropan-1-ol (B10)

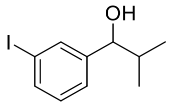

Molecular Weight: 276,12

Synthesised starting from 1-(3-iodophenyl)ethan-1-ol following GP1.

Precatalyst loading has been raised 0.3 mol% and NaO^{t}Bu was used (see also synthesis of B9).

Purification by column chromatography (pentane 90 / 10 diethyl ether). Yield: 63 % (0.634 mmol, 175 mg) as a colourless oil.

^{1}H NMR (500 MHz, $CDCl_3$): δ = 7.69 (d, *J* = 1.4 Hz, 1H), 7.61 (dd, *J* = 7.8, 0.9 Hz, 1H), 7.28 (d, *J* = 6.9 Hz, 1H), 7.08 (t, *J* = 7.7 Hz, 1H), 4.48 – 4.19 (m, 1H), 2.01 – 1.84 (m, 2H), 0.94 (d, *J* = 6.7 Hz, 3H), 0.81 (d, *J* = 6.8 Hz, 3H) ppm.

^{13}C NMR (126 MHz, $CDCl_3$): δ = 146.98, 136.77, 136.06, 130.42, 126.46, 94.56, 79.34, 35.86, 19.26, 18.05 ppm.

2-methyl-1-(4-(trifluoromethyl)phenyl)propan-1-ol (B11)

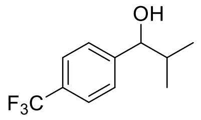

Molecular Weight: 218,22

Synthesised starting from 1-(4-(trifluoromethyll)phenyl))ethan-1-ol following GP1.

Purification by column chromatography (cyclohexane 90 / 10 MTBE). Yield: 74 % (0.738 mmol, 161 mg) as a colourless oil.

^{1}H NMR (500 MHz, $CDCl_3$): δ = 7.60 (d, *J* = 8.1 Hz, 2H), 7.44 (d, *J* = 8.1 Hz, 2H), 4.48 (dd, *J* = 6.3, 2.8 Hz, 1H), 2.01 – 1.91 (m, 1H), 1.89 (s, 1H), 0.97 (d, *J* = 6.7 Hz, 3H), 0.84 (d, *J* = 6.8 Hz, 3H) ppm.

^{13}C NMR (126 MHz, $CDCl_3$): δ = 147.50, 129.96, 129.70, 129.45, 129.19, 126.84, 125.16, 125.13, 125.10, 125.07, 123.12, 79.19, 35.34, 18.90, 17.72 ppm.

1-(4-(*tert*-butyl)phenyl)-2-methylpropan-1-ol (B12)

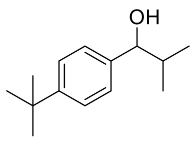

Molecular Weight: 206,33

Synthesised starting from 1-(4-(*tert*-butyl)phenyl))ethan-1-ol following GP1.

Yield (80 %) determined by GC-analysis, Identification by MS

1-(3,4-dimethoxyphenyl)-2-methylpropan-1-ol (B13)

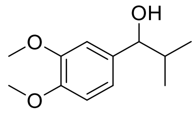

Molecular Weight: 210,27

Synthesised starting from 1-(3,4-dimethoxyphenyl)ethan-1-ol following GP1.

Purification by column chromatography (pentane 60 / 40 diethyl ether). Yield: 70 % (0.699 mmol, 147 mg) as a colourless oil.

^{1}H NMR (500 MHz, $CDCl_3$): δ = 6.86 (s, 1H), 6.80 (s, 2H), 4.26 (d, *J* = 7.2 Hz, 1H), 3.87 (s, 3H), 3.86 (s, 3H), 1.95 – 1.85 (m, 2H), 1.00 (d, *J* = 6.6 Hz, 3H), 0.76 (d, *J* = 6.8 Hz, 3H) ppm.

^{13}C NMR (126 MHz, $CDCl_3$): δ = 148.90, 148.34, 136.49, 118.98, 110.70, 109.51, 80.08, 55.96, 55.92, 35.39, 19.16, 18.61 ppm.

2-methyl-1-(4-(pyrrolidin-1-yl)phenyl)propan-1-ol (B14 /B23)

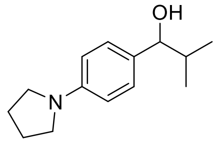

Molecular Weight: 219,33

Synthesised starting from 1-(4-(pyrrolidin-1-yl)phenyl)ethan-1-ol following GP1.

Purification by column chromatography (pentane 89 / 11 MTBE). Yield: 56 % (0.561 mmol, 123 mg) as a yellow oil.

Synthesised starting from 1-(4-(pyrrolidin-1-yl)phenyl)propan-1-ol following GP2:

Purification by column chromatography (pentane 89 / 11 MTBE). Yield: 65 % (0.652 mmol, 143 mg) as a yellow oil.

^{1}H NMR (500 MHz, CD_2Cl_2): δ = 7.04 (d, *J* = 8.5 Hz, 1H), 6.43 (d, *J* = 8.5 Hz, 1H), 4.08 (d, *J* = 7.4 Hz, 1H), 3.21 – 3.13 (m, 1H), 1.95 – 1.88 (m, 1H), 1.80 (dq, *J* = 13.5, 6.8 Hz, 1H), 1.67 (s, 1H), 0.90 (d, *J* = 6.6 Hz, 1H), 0.64 (d, *J* = 6.8 Hz, 1H) ppm.

^{13}C NMR (126 MHz, CD_2Cl_2): δ = 148.11, 131.06, 127.98, 111.70, 80.50, 48.19, 35.84, 25.98, 19.39, 19.10 ppm.

Elemental analysis calcd. for $C_{14}H_{21}NO$: C 76.67, H 9.65, N 6.39; found: C 76.37, H 9.51, N 6.39.

2-methyl-1-(naphthalen-2-yl)propan-1-ol (B15)

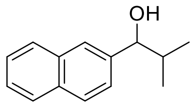

Molecular Weight: 200,28

Synthesised starting from 1-(naphthalen-2-yl))ethan-1-ol following GP1.

Purification by column chromatography (pentane 92 / 8 MTBE). Yield: 79 % (0.793 mmol, 159 mg) as a colourless oil.

^{1}H NMR (500 MHz, CD_2Cl_2): δ = 7.84 (dd, *J* = 8.0, 5.2 Hz, 3H), 7.76 (s, 1H), 7.52 – 7.43 (m, 3H), 4.53 (dd, *J* = 6.7, 3.2 Hz, 1H), 2.12 – 1.94 (m, 2H), 1.01 (d, *J* = 6.7 Hz, 3H), 0.83 (d, *J* = 6.8 Hz, 3H) ppm.

^{13}C NMR (126 MHz, CD_2Cl_2): δ = 142.01, 133.72, 133.46, 128.38, 128.28, 128.10, 126.54, 126.20, 125.83, 125.27, 80.37, 35.81, 19.45, 18.40 ppm.

1-(6-methoxynaphthalen-2-yl)-2-methylpropan-1-ol (B16)

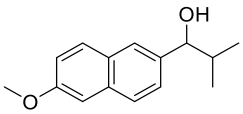

Molecular Weight: 230,31

Synthesised starting from 1-(6-methoxynaphthalen-2-yl)-ethan-1-ol following GP1.

Purification by column chromatography (pentane 85 / 15 MTBE). Yield: 96 % (0.964 mmol, 222 mg) as a colourless oil.

^{1}H NMR (500 MHz, CD_2Cl_2): δ = 7.73 (d, *J* = 8.5 Hz, 2H), 7.68 (s, 1H), 7.42 (dd, *J* = 8.4, 1.6 Hz, 1H), 7.19 – 7.11 (m, 2H), 4.47 (dd, *J* = 6.9, 1.3 Hz, 1H), 3.91 (s, 3H), 2.09 – 1.97 (m, 2H), 1.02 (d, *J* = 6.7 Hz, 3H), 0.81 (d, *J* = 6.8 Hz, 3H) ppm.

^{13}C NMR (126 MHz, CD_2Cl_2): δ = 158.17, 139.71, 134.59, 129.83, 129.11, 127.16, 125.80, 125.74, 119.28, 106.11, 80.44, 55.81, 35.81, 19.44, 18.55 ppm.

2-methyl-1-(pyridin-3-yl)propan-1-ol (B17)

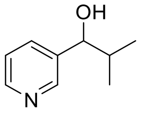

Molecular Weight: 151,21

Synthesised starting from 1-(pyridine-3-yl)-ethan-1-ol following GP1.

Purification by column chromatography (pentane 30 / 70 diethyl ether). Yield: 72 % (0.774 mmol, 117 mg, contains ~5 % diglyme) as a colourless oil.

^{1}H NMR (500 MHz, CD_2Cl_2): δ = 8.52 (d, *J* = 4.8 Hz, 1H), 7.69 (td, *J* = 7.7, 1.7 Hz, 1H), 7.28 – 7.15 (m, 2H), 4.51 (s, 1H), 4.18 (d, *J* = 4.3 Hz, 1H), 2.08 – 1.91 (m, 1H), 0.99 (d, *J* = 6.9 Hz, 3H), 0.74 (d, *J* = 6.8 Hz, 3H) ppm.

^{13}C NMR (126 MHz, CD_2Cl_2): δ = 161.93, 148.41, 136.90, 122.70, 121.51, 77.37, 35.68, 19.78, 16.20 ppm.

2-methyl-1-(ferrocenyl)propanol (B18)

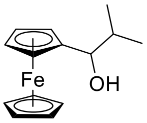

Molecular Weight: 258,14

Synthesised starting from 1-(ferrocenyl)ethan-1-ol following GP1.

Precatalyst loading has been raised 2 mol%, and the reaction time increased to 12 hours.

Yield (76 %) determined by GC-analysis, Identification by MS

1-(2,3-dihydrobenzo[*b*][1,4]dioxin-6-yl)-2-methylpropan-1-ol (B22)

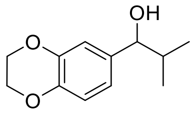

Molecular Weight: 208,26

Synthesised starting from 1-(2,3-dihydrobenzo[*b*][1,4]dioxin-6-yl)ethan-1-ol following GP1.

Purification by column chromatography (pentane 85 / 15 MTBE). Yield: 91 % (0.912 mmol, 190 mg) as a colourless oil.

^{1}H NMR (500 MHz, CD_2Cl_2): δ = 6.79 (d, *J* = 7.8 Hz, 2H), 6.75 (dd, *J* = 8.3, 1.7 Hz, 1H), 4.23 (s, 4H), 4.20 (d, *J* = 7.0 Hz, 1H), 1.91 – 1.80 (m, 2H), 0.96 (d, *J* = 6.7 Hz, 3H), 0.76 (d, *J* = 6.8 Hz, 3H) ppm.

^{13}C NMR (126 MHz, CD_2Cl_2): δ = 143.82, 143.32, 137.95, 120.03, 117.17, 115.82, 79.89, 65.01, 64.98, 35.81, 19.32, 18.62 ppm.

2-methyl-1,3-diphenylpropan-1-ol (B24)

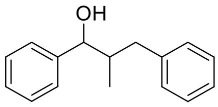

Molecular Weight: 226,32

Synthesised starting from 1,3-diphenylpropan-1-ol following GP2.

Purification by column chromatography (pentane 92 / 8 MTBE). Yield: 80 % (0.796 mmol, 180 mg) as a colourless oil.

^{1}H NMR (500 MHz, $CDCl_3$): δ = 7.39 – 7.26 (m, 14H), 7.23 – 7.13 (m, 6H), 4.61 (t, *J* = 4.3 Hz, 1H), 4.50 (dd, *J* = 7.1, 3.1 Hz, 1H), 3.08 (dd, *J* = 13.3, 3.8 Hz, 1H), 2.79 (dd, *J* = 13.4, 5.8 Hz, 1H), 2.39 (ddd, *J* = 13.4, 9.4, 6.2 Hz, 2H), 2.22 – 2.07 (m, 2H), 1.89 (d, *J* = 3.1 Hz, 1H), 1.80 (d, *J* = 3.8 Hz, 1H), 0.86 (d, *J* = 6.7 Hz, 3H), 0.68 (d, *J* = 6.8 Hz, 3H) ppm.

^{13}C NMR (126 MHz, $CDCl_3$): δ = 143.77, 143.48, 141.08, 140.97, 129.51, 129.25, 128.47, 128.41, 128.38, 128.32, 127.76, 127.44, 126.83, 126.36, 125.99, 125.91, 78.89, 77.08, 42.54, 42.29, 39.88, 38.79, 15.67, 13.92 ppm.

2-(methylamino)-1-phenylpropan-1-ol (B25)

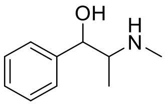

Molecular Weight: 165,24

The catalyst loading was increased to 0.5 mol%, the reaction time was increased to 6 h.

Synthesised starting from 1-(methylamino)-1-phenylethan-1-ol following GP2.

Purification by column chromatography (methylene chloride 95 / 5 methanol). Yield: 80 % (0.799 mmol, 132 mg) as a colourless solid.

Upscaling starting from 1-(methylamino)-1-phenylethan-1-ol following GP4.

Purification by crystallisation from diethyl ether / pentane. Yield: 76 % (36.1 mmol, 5.97 g) as a colourless solid.

^{1}H NMR (500 MHz, CD_2Cl_2): δ = 7.38 – 7.22 (m, 5H), 4.11 (d, *J* = 8.2 Hz, 1H), 2.55 (dq, *J* = 12.9, 6.4 Hz, 1H), 2.42 (s, 3H), 0.91 (d, *J* = 6.4 Hz, 3H) ppm.

^{13}C NMR (126 MHz, CD_2Cl_2): δ = 143.30, 128.64, 127.57, 126.59, 77.99, 62.08, 33.84, 15.92 ppm.

1-phenyl-2-(piperidin-1-yl)propan-1-ol (B26)

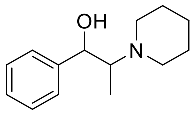

Molecular Weight: 219,33

Synthesised starting from 1-phenyl-2-(piperidin-1-yl)ethan-1-ol following GP2.

Purification by column chromatography (pentane 80 / 20 ethyl acetate, + 1 % NEt_3). Yield: 84 % (0.843 mmol, 185 mg) as a colourless oil.

^{1}H NMR (500 MHz, $CDCl_3$): δ = 7.30 – 7.17 (m, 5H), 4.15 (d, *J* = 9.7 Hz, 1H), 2.62 (t, *J* = 7.3 Hz, 2H), 2.46 (dq, *J* = 9.7, 6.7 Hz, 1H), 2.31 (s, 2H), 1.66 – 1.49 (m, 4H), 1.41 (s, 2H), 1.18 (s, 1H), 0.69 (d, *J* = 6.7 Hz, 3H) ppm.

^{13}C NMR (126 MHz, $CDCl_3$): δ = 142.50, 128.32, 127.74, 127.51, 74.34, 67.20, 49.46, 26.70, 24.83, 8.03 ppm.

2-(cyclohexylamino)-6-methylcyclohexan-1-ol (B27)

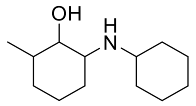

Synthesised starting from 2-(cyclohexylamino)cyclohexan-1-ol following GP2.

Precatalyst loading has been raised 2 mol%, and the reaction time increased to 12 hours.

Yield (68 %) determined by GC-analysis, Identification by MS

2-(4-methoxyphenyl)propan-1-ol (B28)

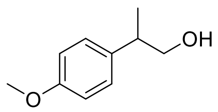

Molecular Weight: 166,22

Synthesised starting from 2-(4-methoxyphenyl)ethan-1-ol following GP2.

Purification by column chromatography (pentane 80 / 20 MTBE). Yield: 84 % (0.842 mmol, 140 mg) as a colourless oil.

^{1}H NMR (500 MHz, $CDCl_3$): δ = 7.19 – 7.12 (m, 2H), 6.90 – 6.84 (m, 2H), 3.80 (s, 3H), 3.71 – 3.59 (m, 2H), 2.90 (h, *J* = 7.0 Hz, 1H), 1.38 (s, 1H), 1.25 (d, *J* = 7.0 Hz, 3H) ppm.

^{13}C NMR (126 MHz, $CDCl_3$): δ = 158.46, 135.69, 128.52, 114.17, 68.94, 55.39, 41.70, 17.86 ppm.

2-methyl-3-phenylpropan-1-ol (B29)

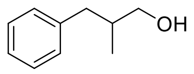

Molecular Weight: 150,22

Synthesised starting from 3-phenylpropan-1-ol following GP2.

Yield (97 %) determined by GC-analysis, Identification by MS

2,3,7-trimethyloct-6-en-1-ol (B30)

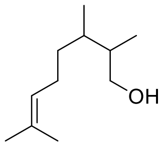

Molecular Weight: 170,30

Synthesised starting from 3,7-dimethyloct-6-en-1-ol following GP2. The reaction time was increased to 6 hours.

Purification by column chromatography (pentane 90 / 10 MTBE). Yield: 86 % (0.857 mmol, 146 mg) as a colourless oil.

^{1}H NMR (500 MHz, $CDCl_3$): δ = 5.16 – 5.04 (m, 1H), 3.73 – 3.39 (m, 2H), 2.11 – 1.81 (m, 2H), 1.68 (s, 3H), 1.60 (s, 4H), 1.56 – 1.46 (m, 1H), 1.42 – 1.27 (m, 2H), 1.26 – 1.06 (m, 1H), 0.89 (dd, J = 6.9, 3.8 Hz, 2H), 0.80 (dd, J = 9.9, 6.8 Hz, 3H) ppm.

^{13}C NMR (126 MHz, $CDCl_3$): δ = 131.52, 131.43, 124.94, 124.87, 66.97, 66.23, 40.81, 39.66, 35.19, 34.19, 33.05, 32.92, 26.09, 26.08, 25.86, 17.80, 17.79, 17.02, 14.38, 13.62, 11.46 ppm.

2,4-dimethylnonan-3-ol (B31)

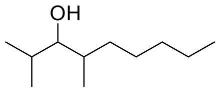

Molecular Weight: 172,31

Synthesised starting from 2-octanol following GP1.

Precatalyst loading has been raised 2 mol%, the amount of MeOH was increased to 8 eq and the reaction time increased to 12 hours.

Yield (73 %) determined by GC-analysis, Identification by MS

2,12-dimethylcyclododecan-1-ol (B32)

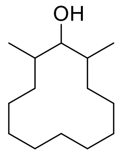

Molecular Weight: 212,38

Synthesised starting from cyclododecanol following GP2.

Precatalyst loading has been raised 5 mol%, the amount of MeOH was increased to 7 eq and the reaction time increased to 12 hours.

Yield (61 %) determined by GC-analysis, Identification by MS

Mechanistic control experiments

Preparation of [Mn-IIIaH]K$_2$ starting from [Mn-IIIa]

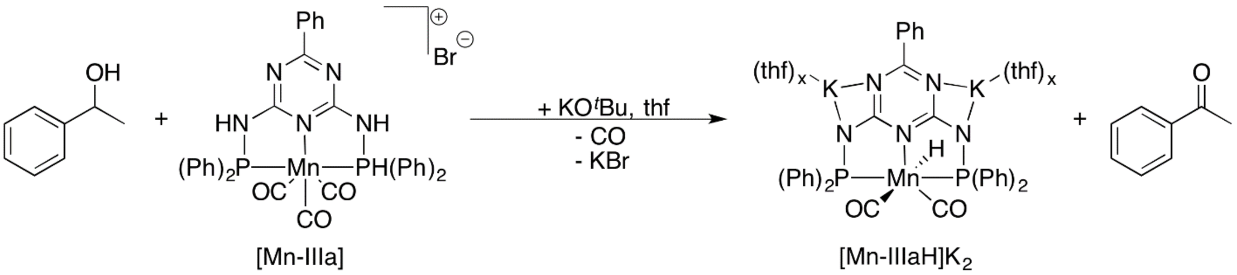

The complex **[Mn-IIIaH]K$_2$** was synthesized starting from **[Mn-IIIa]**.

In a glovebox 25.8 µmol **[Mn-IIIa]** (20 mg) and (350 µmol) KO^{t}Bu were dissolved in 700 µL thf-d$_8$, 250 µmol 1-phenylethanol was added, the solution was filtrated and placed in a Young NMR tube. The reaction mixture was heated to 120 °C for 4 h and analyzed by NMR. ^{31}P and ^{1}H NMR analytics indicates that the species **[Mn-IIIaH]K$_2$** is formed (see figure S4).

^{1}H NMR (500 MHz, thf-d$_8$): δ = - 4,81 (t, *J* = 48.4 Hz, 1H) ppm.

NMR (202 MHz, thf-d$_8$): δ = 129.0 ppm.

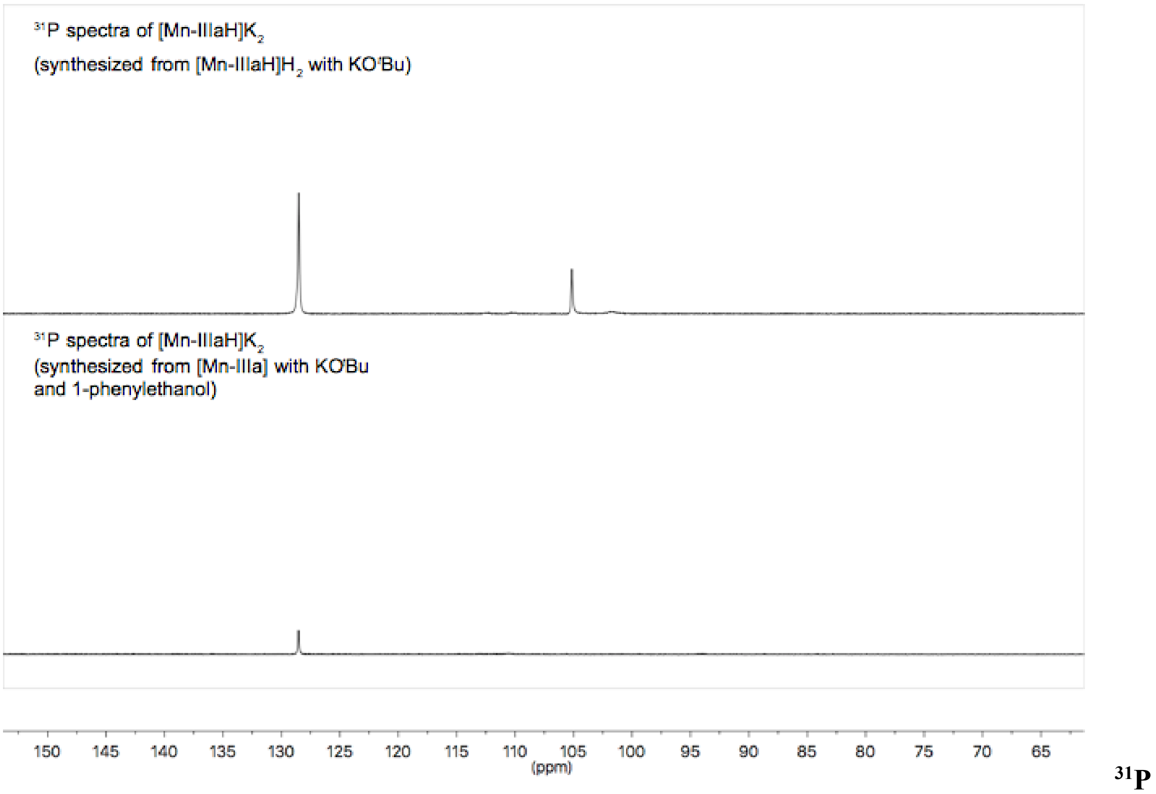

Figure S4: ^{31}P analytic of **[Mn-IIIaH]K$_2$**, top: ^{31}P spectra of **[Mn-IIIaH]K$_2$** synthesized from **[Mn-IIIaH]H$_2$** with 2 eq KO^{t}Bu. Bottom: ^{31}P spectra of **[Mn-IIIaH]K$_2$** synthesized from **[Mn-IIIa]**, KO^{t}Bu and 1-phenylethanol.

Additionally, the ^{13}C NMR spectra shows a resonance typical for carbonyl compounds.

The reaction mixture was quenched with water, extracted with diethyl ether and dried over Na_2SO_4. The organic layer was analysed by GC-MS. It was possible to detect aldol coupling compounds of acetophenone.

Hydrogenation of the C=C double bond in a chalcone compound at room temperature

The hydrogenation of the C=C double bond of (*E*)-chalcone was chosen as model reaction.

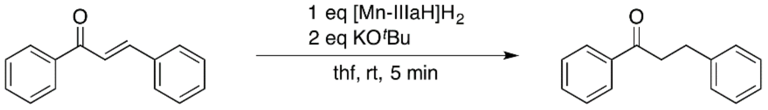

In a glovebox, 37.5 µmol **[Mn-IIIaH]H₂** (25 mg), 75 µmol KO^{t}Bu (8.4 mg, 2 eq), and 37.5 µmol (*E*)-chalcone (7.8 mg) were consecutively added to a 1 mL Schlenk-tube, dissolved in 800 µL dry thf and stirred for 5 minutes. The tube was sealed, taken outside the Glovebox, quenched with water, extracted with diethyl ether and dried over Na_2SO_4. GC-MS analysis of the organic phase indicates that 1,3-diphenylpropan-1-one is produced quantitatively.

Hydrogenation of the C=O and C=C double bonds in a chalcone compound at 120 °C

The hydrogenation of the C=O and C=C double bonds of (*E*)-chalcone was chosen as model reaction.

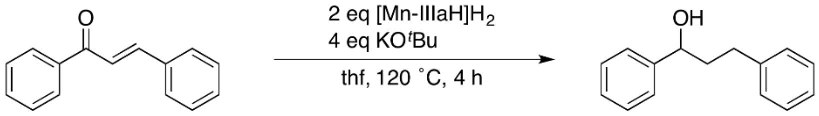

In a glovebox, 44.9 µmol **[Mn-IIIaH]H₂** (30 mg), 90 µmol KO^{t}Bu (10.07 mg, 2 eq), and 22.45 µmol (*E*)-chalcone (4.72 mg) were consecutively added to a 1 mL Schlenk-tube, dissolved in 1000 µL dry thf. The tube was sealed, taken outside the Glovebox and placed in a heated oil bath at 120 ˚C for 4 h. After cooling down to room temperature the reaction was quenched with water, extracted with diethyl ether and dried over Na_2SO_4. GC analysis of the organic phase indicates that 1,3-diphenylpropan-1-ol is produced with a yield of 88 %.

Hydrogenation of isobutyrophenone

The hydrogenation of the isobutyrophenone was chosen as model reaction.

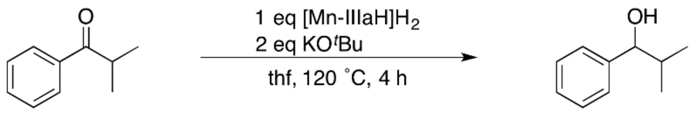

In a glovebox, 37.5 µmol **[Mn-IIIaH]H$_2$** (25 mg), 2 eq KO^{t}Bu (75 µmol, 8.4 mg) and 37.5 µmol isobutyrophenone (5.6 µL) were consecutively added to a 2 mL Schlenk-tube, dissolved in 800 µL dry thf and stirred for 5 minutes. A sample for GC-MS analysis was taken, afterwards the tube was sealed, taken outside the Glovebox, and placed in a heated oil bath at 120 ˚C for 4 h. After the reaction, the tube was cooled to room temperature, quenched with water, extracted with diethyl ether and dried over Na_2SO_4. GC-MS analysis of the organic phase indicates that 2-phenyl-1-methylpropan-1-ol is produced in a yield of 90 %.

Hydrogenation activity of [Mn-IIIaH]K$_2$, [Mn-IIIaH]HK, [Mn-IIIaH]H$_2$

The hydrogenation of the isobutyrophenone was chosen as model reaction to verify that [Mn-IIIaH]K$_2$ is actually the active species. The reaction temperature and time were chosen analogues to the hydrogenation of the C=C and C=O double bonds *vide supra*.

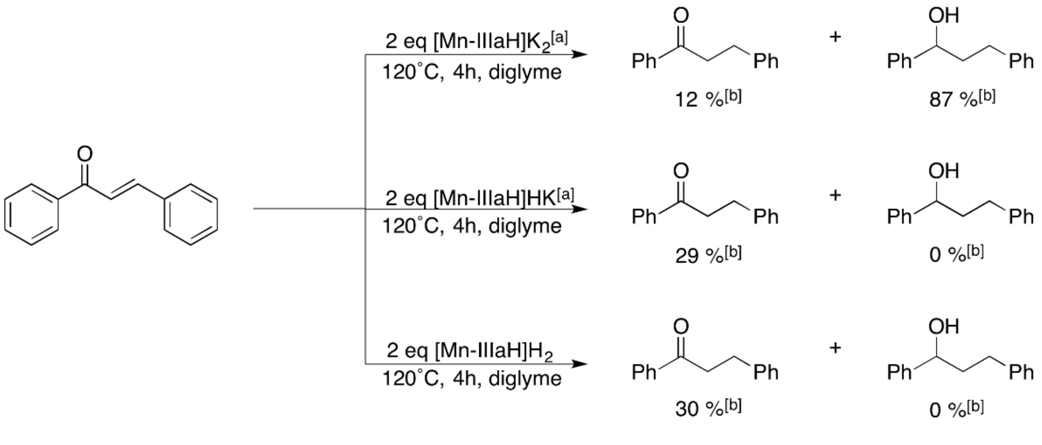

[a] *in situ* generated. [b] Yields of were determined by GC-analysis using *n*-decane as internal standard.

Figure S5: overview of the Hydrogenation activity of [Mn-IIIaH]K$_2$, [Mn-IIIaH]HK, [Mn-IIIaH]H$_2$

In a glovebox, 44.9 µmol **[Mn-IIIaH]H$_2$** (30 mg), 90 µmol KO^{t}Bu (10.07 mg, 2 eq), and 22.45 µmol (*E*)-chalcone (4.72 mg) were consecutively added to a 1 mL Schlenk-tube, dissolved in 1000 µL dry diglyme. The tube was sealed, taken outside the Glovebox and placed in a heated oil bath at 120 ˚C for 4 h. After cooling down to room temperature the reaction was quenched with water, extracted with diethyl ether and dried over Na_2SO_4. GC analysis of the

organic phase indicates that 1,3-diphenylpropan-1-ol is produced with a yield of 87 %; 1,3-diphenylpropan-1-one is produced with a yield of 12%

In a glovebox, 44.9 µmol **[Mn-IIIaH]H$_2$** (30 mg), 44.9 µmol KO^{t}Bu (5.03 mg, 1 eq), and 22.45 µmol (*E*)-chalcone (4.72 mg) were consecutively added to a 1 mL Schlenk-tube, dissolved in 1000 µL dry diglyme. The tube was sealed, taken outside the Glovebox and placed in a heated oil bath at 120 ˚C for 4 h. After cooling down to room temperature the reaction was quenched with water, extracted with diethyl ether and dried over Na_2SO_4. GC analysis of the organic phase indicates that no 1,3-diphenylpropan-1-ol is produced;1,3-diphenylpropan-1-one is produced with a yield of 29 %.

In a glovebox, 44.9 µmol **[Mn-IIIaH]H$_2$** (30 mg) and 22.45 µmol (*E*)-chalcone (4.72 mg) were consecutively added to a 1 mL Schlenk-tube, dissolved in 1000 µL dry diglyme. The tube was sealed, taken outside the Glovebox and placed in a heated oil bath at 120 ˚C for 4 h. After cooling down to room temperature the reaction was quenched with water, extracted with diethyl ether and dried over Na_2SO_4. GC analysis of the organic phase indicates that no 1,3-diphenylpropan-1-ol is produced;1,3-diphenylpropan-1-one is produced with a yield of 30 %.

Temperature studies with [Mn-IIIaH]K$_2$

In order to examine the temperature stability of **[Mn-IIIaH]K$_2$**, 53 µmol (40 mg) of the complex were dissolved in 1 mL of thf-d_8 and placed in a NMR-tube with Young valve (glovebox) and heated to 50 °C for 12 h. ^{1}H and ^{31}P NMR analytics show that the complex eliminates hydrogen an starting to decompose. Further heating to 140 °C for additionally 3 h led to complete decomposition of **[Mn-IIIaH]K$_2$**.

Another temperature study under was carried out to prove that the complex **[Mn-IIIaH]K$_2$** is stable under similar to catalysis conditions. For this experiment 53 µmol of **[Mn-IIIaH]K$_2$** (40 mg) were dissolved in 1 ml of thf-d_8,0.250 µmol 1-phenylethanol, 375 µmol KO^{t}Bu were added and the solution was placed in a NMR-tube with Young valve (glovebox). The NMR-tube taken outside the glovebox, analyzed by ^{1}H and ^{31}P NMR and placed in a heated oil bath at 140 °C. The probe was analyzed after 1, 3 and 24 h of heating by ^{1}H and ^{31}P NMR.

NMR analysis shows that the complex **[Mn-IIIaH]K$_2$** is stable in presence of an secondary alcohol and base over a long period of time. Nearly no decomposition could be detected after 3 h of heating at 140 °C. After 24 h at 140 °C partly decomposition was noticeable.

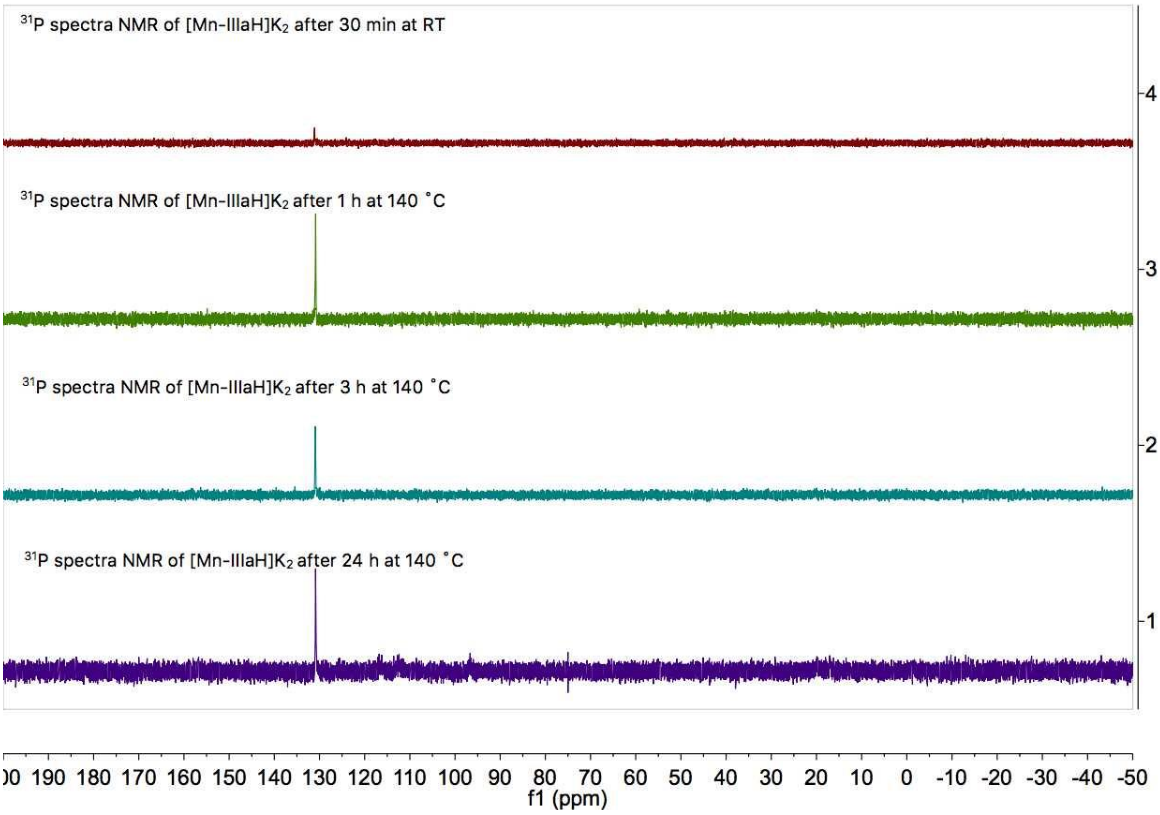

Figure S6: ^{31}P analytic of **[Mn-IIIaH]K$_2$** after prolonged heating.

In order to prove that the complex is still active in the double methylation of secondary alcohols after prolonged heating under the chosen conditions, the 3 h tempered complex was used in the catalysis. The double methylation of 1-(4-methoxyphenyl)ethan-1-ol following GP1 was chosen as model reaction.

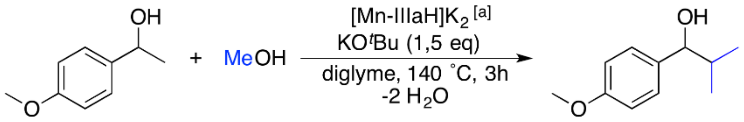

Table S10: Testing the catalyst activities after heating to 140 °C for 3 h

Precatalyst loading [**Mn-IIIaH**]$\mathbf{K_2}$ after heating to 140 °C for 3 h in thf-d_8[mol%]	Yield 1-(4-methoxyphenyl)-2-methylpropan-1-ol [%]
0.1	88
0.5	83
1	81

[a] Reaction conditions: [**Mn-IIIaH**]$\mathbf{K_2}$, KO^{t}Bu (1.5 eq), 1-(4-methoxyphenyl)ethan-1-ol (1 mmol), MeOH (3 mmol, 122 µL), *diglyme*, 140 °C (oil bath), 3 h. Yields of were determined by GC-analysis using *n*-decane as internal standard.

As shown in table S10, the catalyst remains active after heating under similar to catalysis conditions.

Detection of hydrogen in headspace of the reaction

In a glovebox, 1 mmol of 1-phenylethanol, 1.5 eq KO^{t}Bu (1.5 mmol, 168 mg), 0.1 mol% precatalyst (1 µmol, 200 µL of a 5 µM stock solution in diglyme), 3eq of methanol (3 mmol, 122 µL), and 1.8 mL diglyme were consecutively added to a pressure tube. The tube was sealed with a septum, taken outside of the glovebox, placed in a heated oil bath at 140 °C and stirred for 3 h. Afterwards, the pressure tube was cooled to room temperature, the gas mixture in the headspace of reaction was analyzed by gas-chromatography with methane as internal standard (see Figure S5 below).

Afterwards, the pressure tube was opened, and the reaction was quenched by addition of water (1 mL). The aqueous layer was extracted with MTBE, the organic layers were combined, and dried using Na_2SO_4. The crude product was analyzed by GC.

Yield of 2-phenyl-1-methylpropan-1-ol: 90 % (GC)

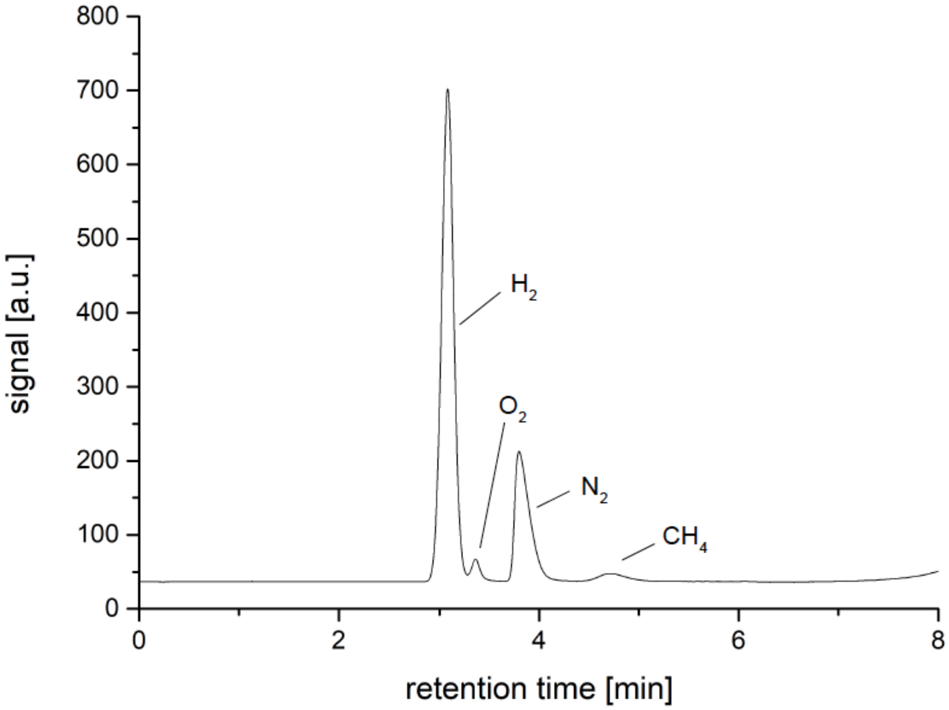

Figure S5: Chromatogram of the gas-chromatographic analysis of the gas mixture in the headspace over the reaction mixture after the reaction.

References

[1] O.V. Dolomanov, L. J. Bourhis, R. J. Gildea, J. A. K. Howard, H. Puschmann, *J. Appl. Cryst.* **2009**, *42*, 339- 341.

[2] G. M. Sheldrick, *Acta Cryst.* **2015**, *C71*, 3–8.

[3] L. J. Farrugia, *J. Appl. Cryst.* **2012**, *45*, 849–854.

[4] C. F. Macrae, I. J. Bruno, J. A. Chisholm, P. R. Edgington, P. McCabe, E. Pidcock, L. Rodriguez-Monge, R. Taylor, J. van de Streek, P. A. Wood, *J. Appl. Cryst.* **2008**, *41*, 466-470.

[5] S. Michlik, R. Kempe, *Nat. Chem.* **2013**, *5*, 140-144.

[6] S. Rösler, M. Ertl, T. Irrgang, R. Kempe, *Angew. Chem. Int. Ed.* **2015**, *54*, 15046-15050

[7] F. Kallmeier, T. Irrgang, T. Dietel, R. Kempe, *Angew. Chem. Int. Ed.* **2016**, *55*, 11806-11809.

[8] S. Michlik, R. Kempe, *Angew. Chem. Int. Ed.* **2013**, *52*, 6326-6329.

[9] G. Zhang, T. Irrgang, T. Dietel, F. Kallmeier, R. Kempe, *Angew. Chem. Int. Ed.* **2018**, *57*, 9131-9135.

[10] N. Deibl, R. Kempe, *Angew. Chem. Int. Ed.* **2017**, *56*, 1663-1666.

[11] A. M. Tondreau, J. M. Boncella, *Polyhedron* **2016**, *116*, 96-104.

[12] M. Mastalir, B. Stöger, E. Pittenauer, M. Puchberger, G. Allmaier, K. Kirchner, *Adv. Synth. Catal.* **2016**, *358*, 3824 -3831.

[13] R. Soni, T. H. Hall, B. P. Mitchell, M. R. Owen, M. Wills, *J. Org. Chem.* **2015**, *80*, 6784–6793.

[14] C. E. Harris, G. B. Fisher, D. Beardsley, L. Lee, C. T. Goralski, L. W. Nicholson, B. Singaram, *J. Org. Chem.* **1994**, *59*, 7746–7751.

Characterization data

Characterization of complexes

[Mn-IIIa]

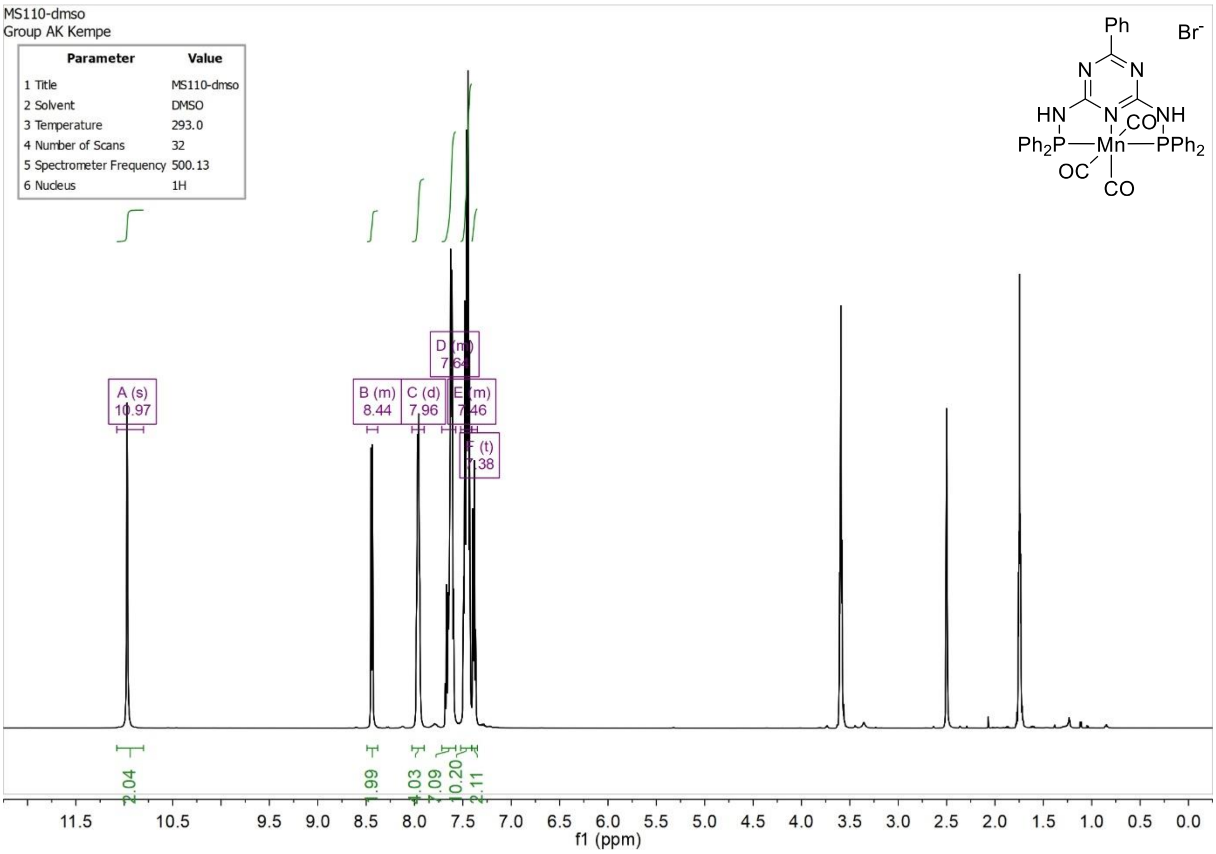

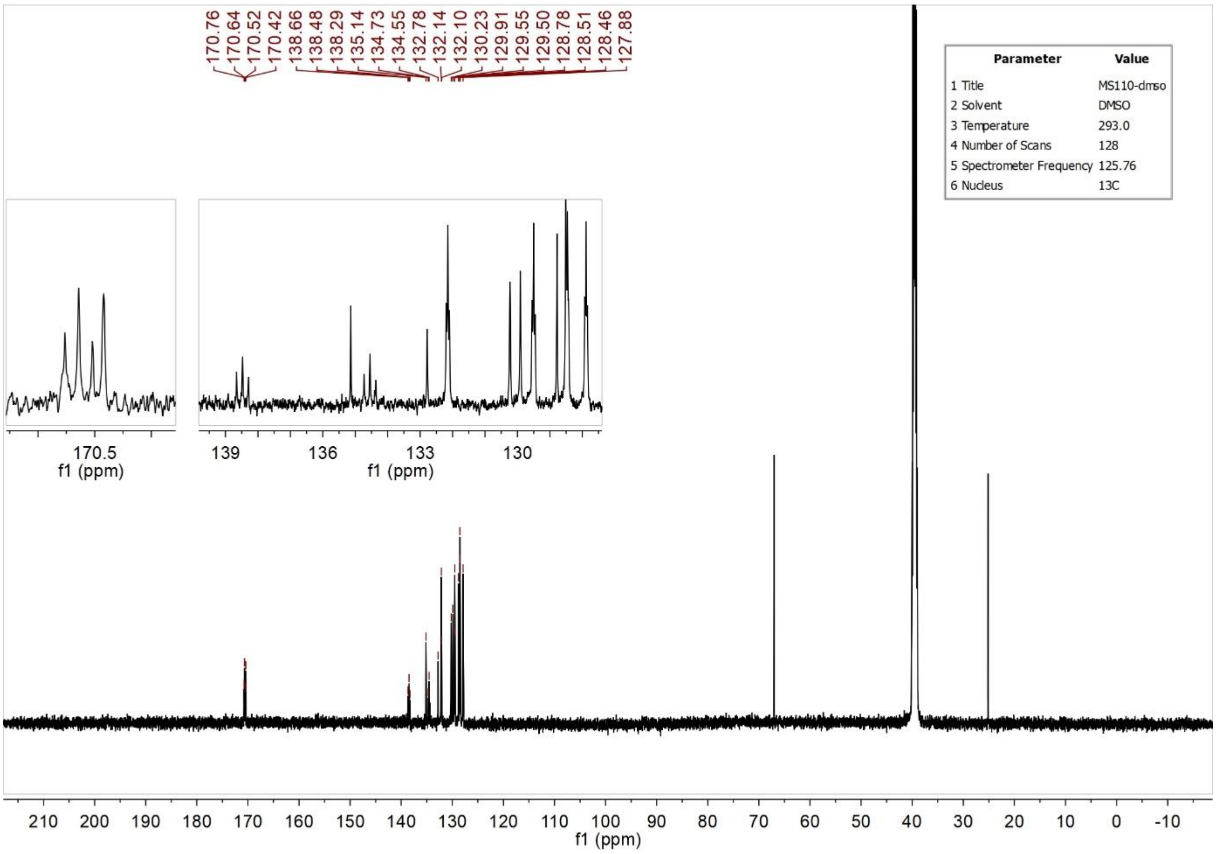

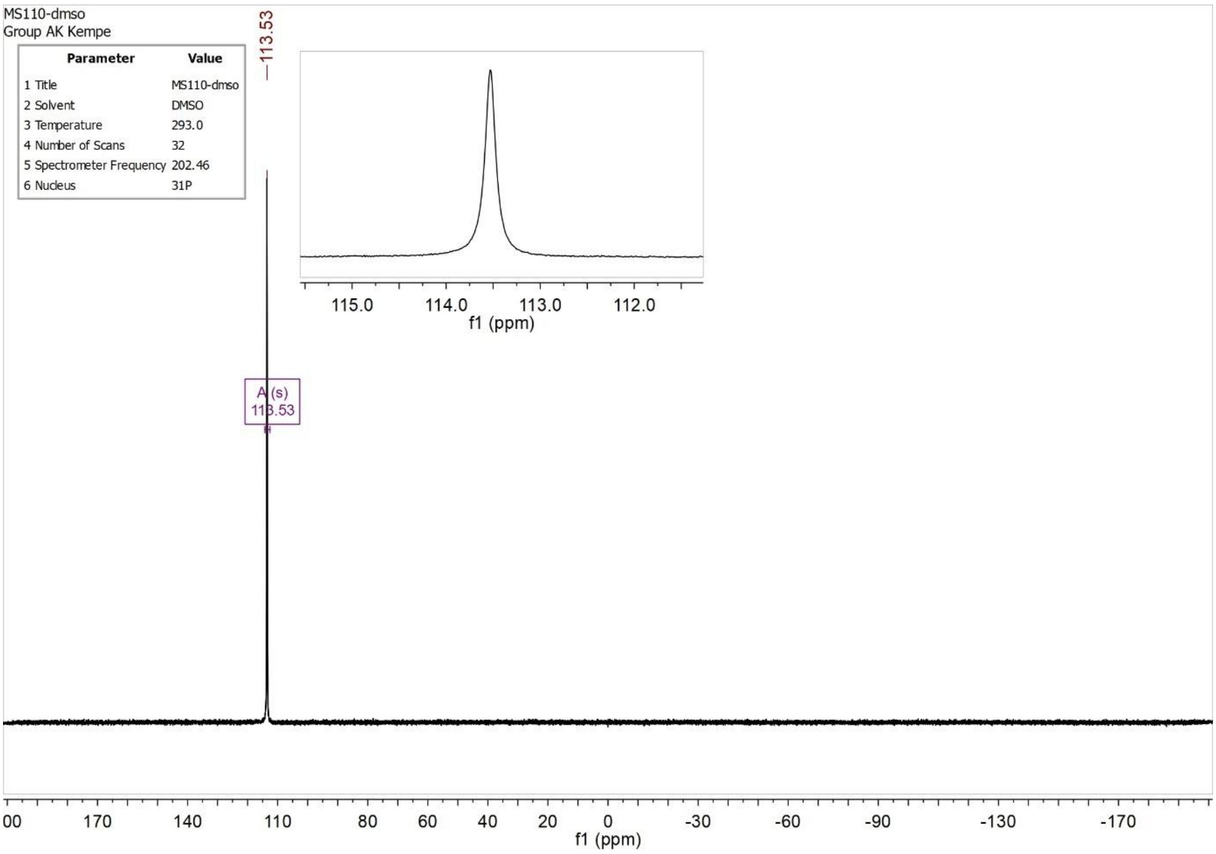
MS110-dmso
Group AK Kempe
Parameter | Value
1 Title MS110-dmso
2 Solvent DMSO
3 Temperature 293.0
4 Number of Scans 32
5 Spectrometer Frequency 202.46
6 Nucleus 31P
—113.53
A (s) 113.53
115.0 114.0 113.0 112.0
f1 (ppm)
00 170 140 110 80 60 40 20 0 -30 -60 -90 -130 -170
f1 (ppm)

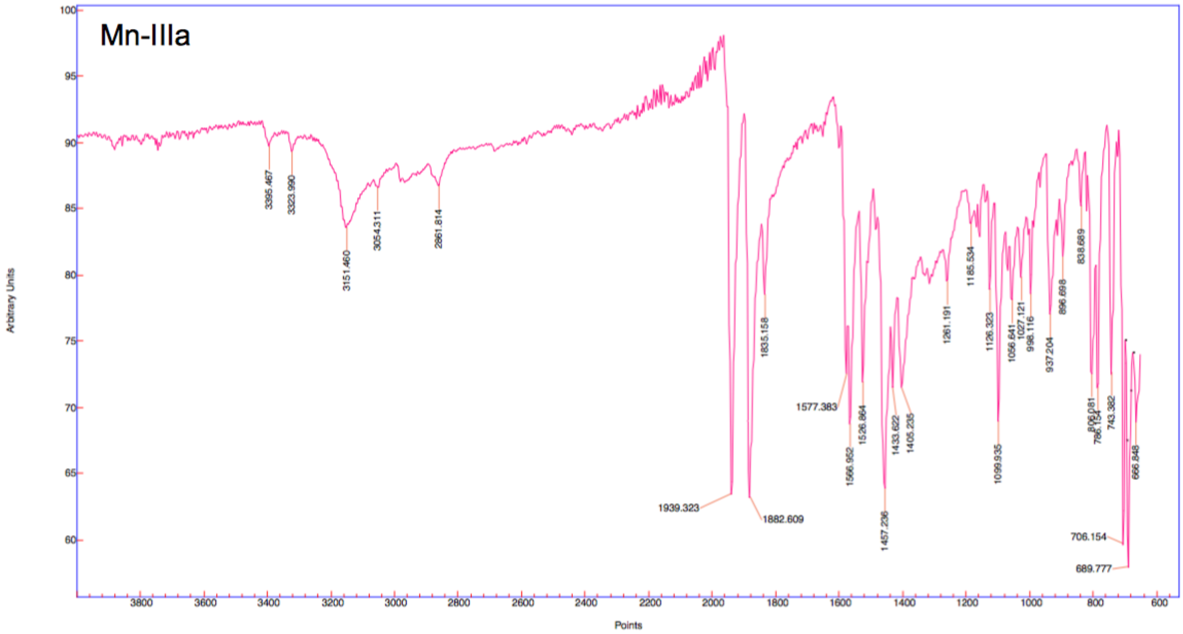
Mn-IIIa
Arbitrary Units
Points
3395.467
3323.990
3151.460
3054.311
2861.814
1939.323
1882.609
1835.158
1577.383
1566.952
1526.864
1457.236
1433.622
1405.235
1261.191
1185.534
1126.323
1099.935
1056.641
1027.121
998.116
937.204
896.698
838.689
806.081
743.382
706.154
689.777
666.848

[**Mn-IIIb**]

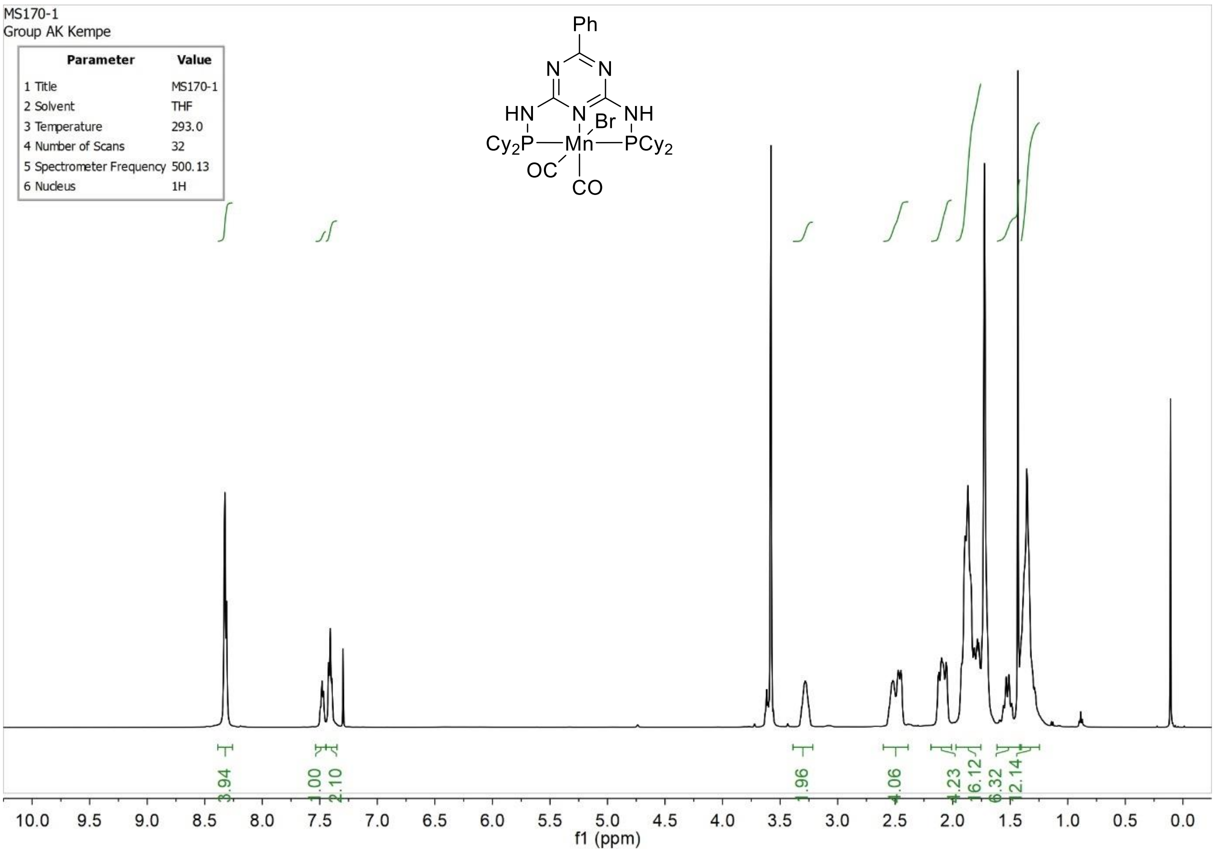
MS170-1
Group AK Kempe
Parameter | Value
1 Title | MS170-1
2 Solvent | THF
3 Temperature | 293.0
4 Number of Scans | 32
5 Spectrometer Frequency | 500.13
6 Nucleus | 1H
Ph
N
N
HN
N
NH
Br
Cy2P
Mn
PCy2
OC
CO
3.94
1.00
2.10
1.96
4.06
4.23
16.12
6.32
12.14
f1 (ppm)

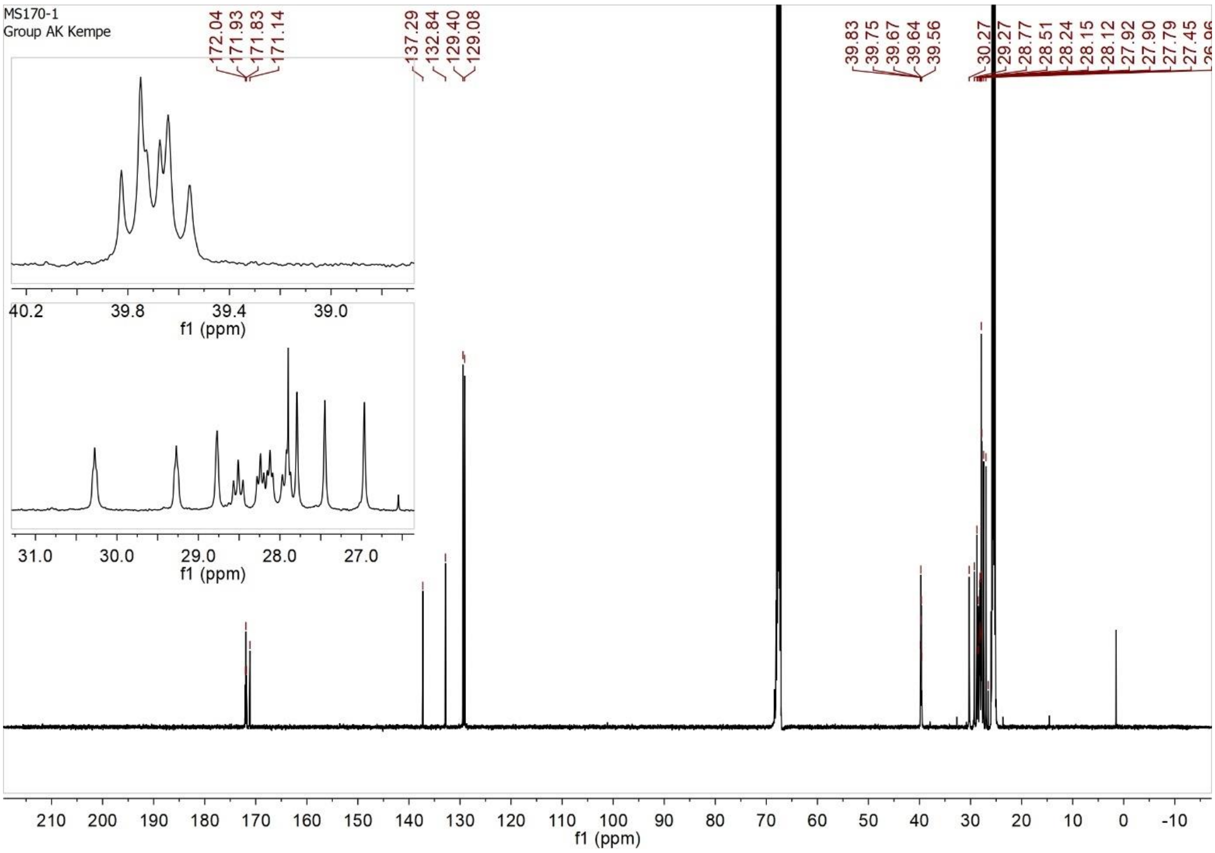
MS170-1
Group AK Kempe
172.04
171.93
171.83
171.14
137.29
132.84
129.40
129.08
39.83
39.75
39.67
39.64
39.56
30.27
29.27
28.77
28.51
28.24
28.15
28.12
27.92
27.90
27.79
27.45
f1 (ppm)

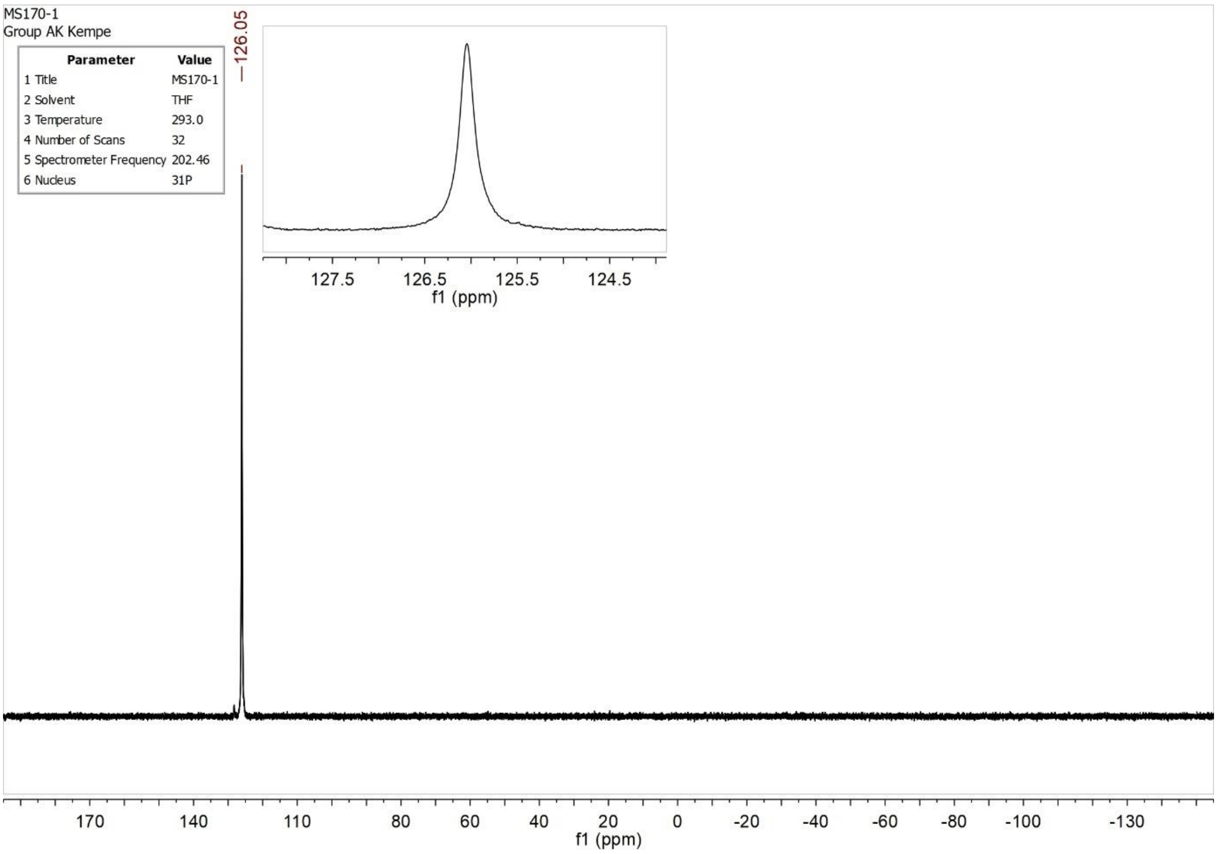
MS170-1
Group AK Kempe
Parameter Value
1 Title MS170-1
2 Solvent THF
3 Temperature 293.0
4 Number of Scans 32
5 Spectrometer Frequency 202.46
6 Nucleus 31P
—126.05
127.5
126.5
125.5
124.5
f1 (ppm)
170
140
110
80
60
40
20
0
-20
-40
-60
-80
-100
-130
f1 (ppm)

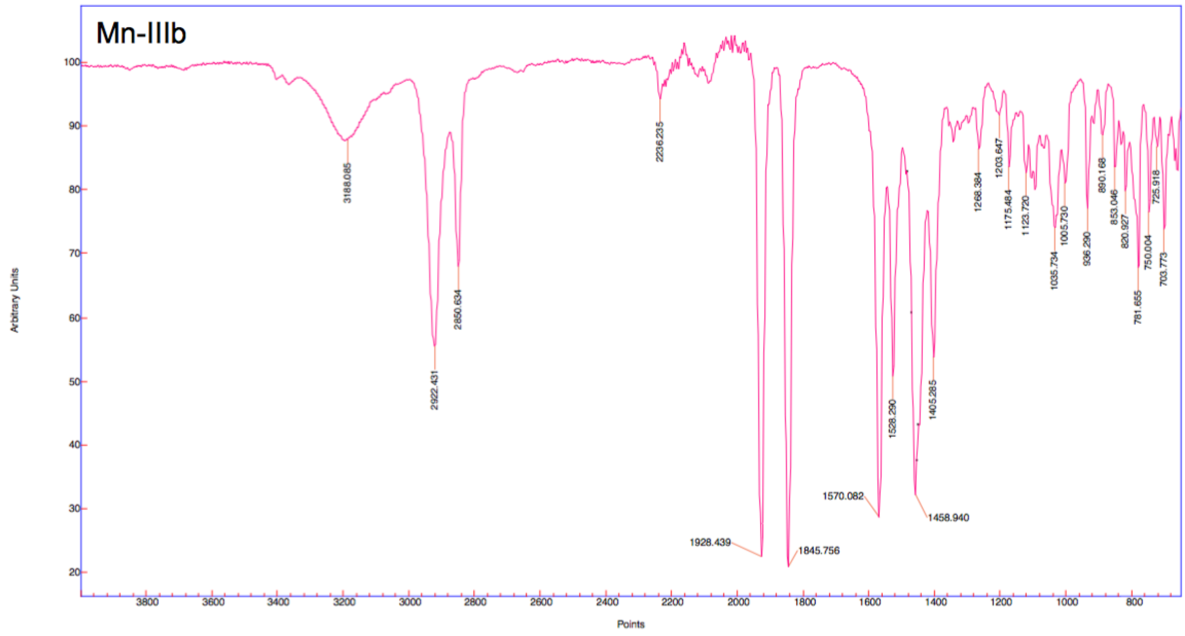
Mn-IIIb
Arbitrary Units
Points
3188.085
2922.431
2850.634
2236.235
1928.439
1845.756
1570.082
1528.290
1458.940
1405.285
1268.384
1203.647
1175.484
1123.720
1035.734
1005.730
936.290
890.168
853.046
820.927
781.655
750.004
725.918
703.773

[**Mn-IIIaH**]H_2

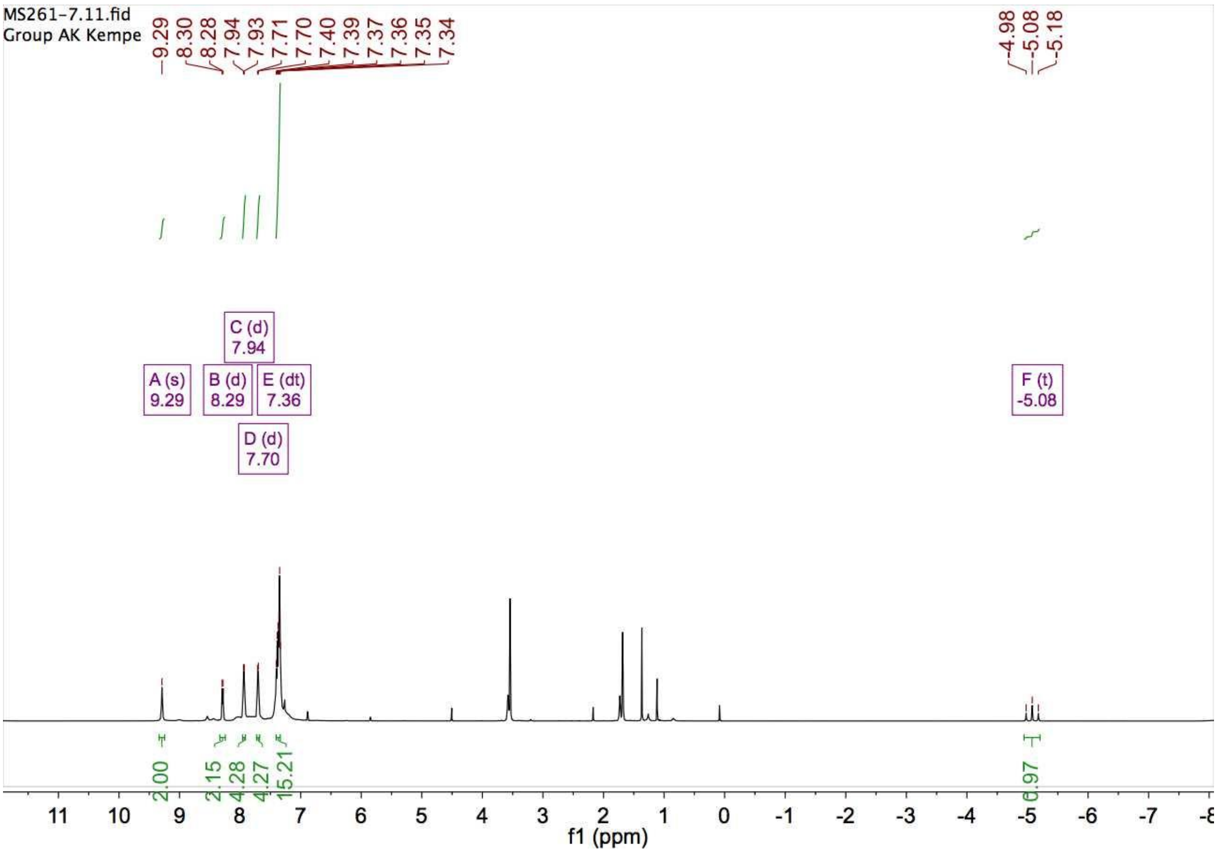
MS261-7.11.fid
Group AK Kempe
9.29
8.30
8.28
7.94
7.93
7.71
7.70
7.40
7.39
7.37
7.36
7.35
7.34
-4.98
-5.08
-5.18
A (s) 9.29
B (d) 8.29
C (d) 7.94
D (d) 7.70
E (dt) 7.36
F (t) -5.08
2.00
2.15
4.28
4.27
15.21
0.97
f1 (ppm)

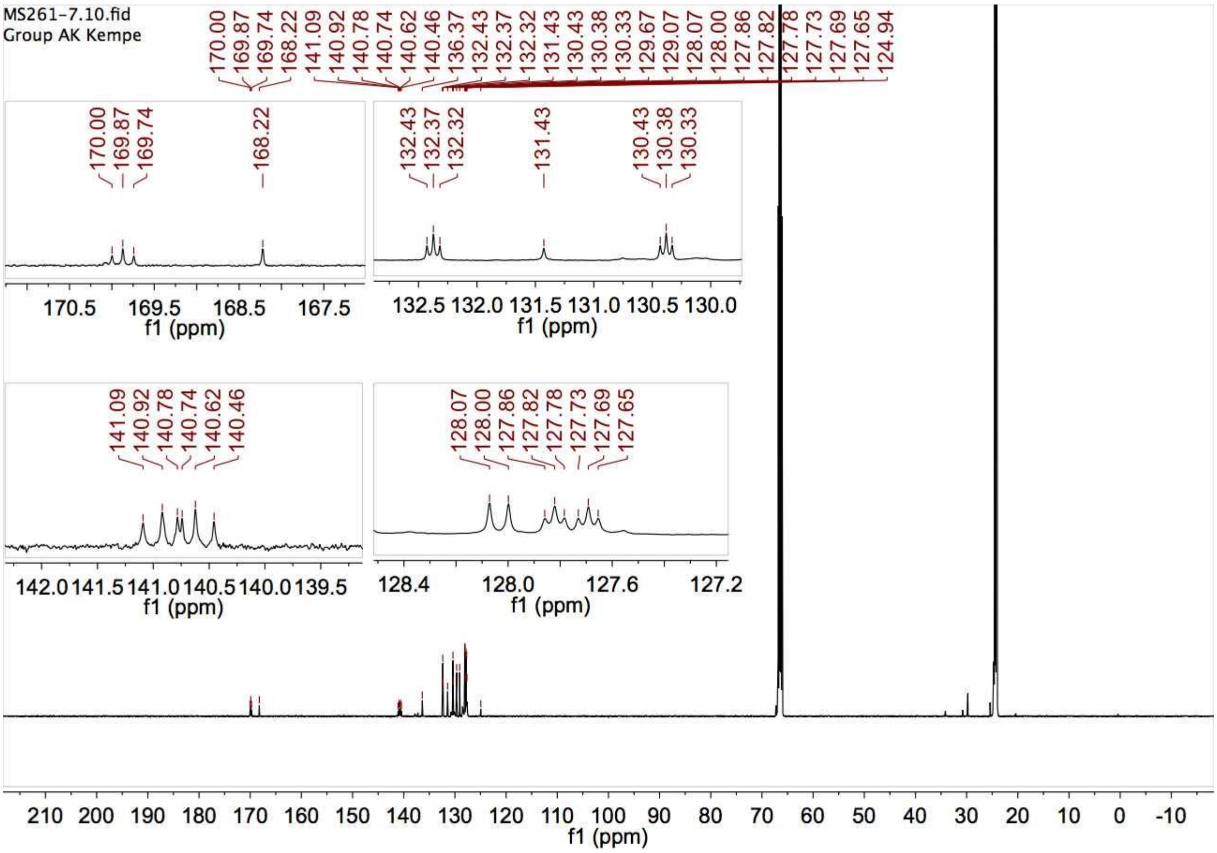
MS261-7.10.fid
Group AK Kempe
170.00
169.87
169.74
168.22
141.09
140.92
140.78
140.74
140.62
140.46
136.37
132.43
132.37
132.32
131.43
130.43
130.38
130.33
129.67
129.07
128.07
128.00
127.86
127.82
127.78
127.73
127.69
127.65
124.94
f1 (ppm)

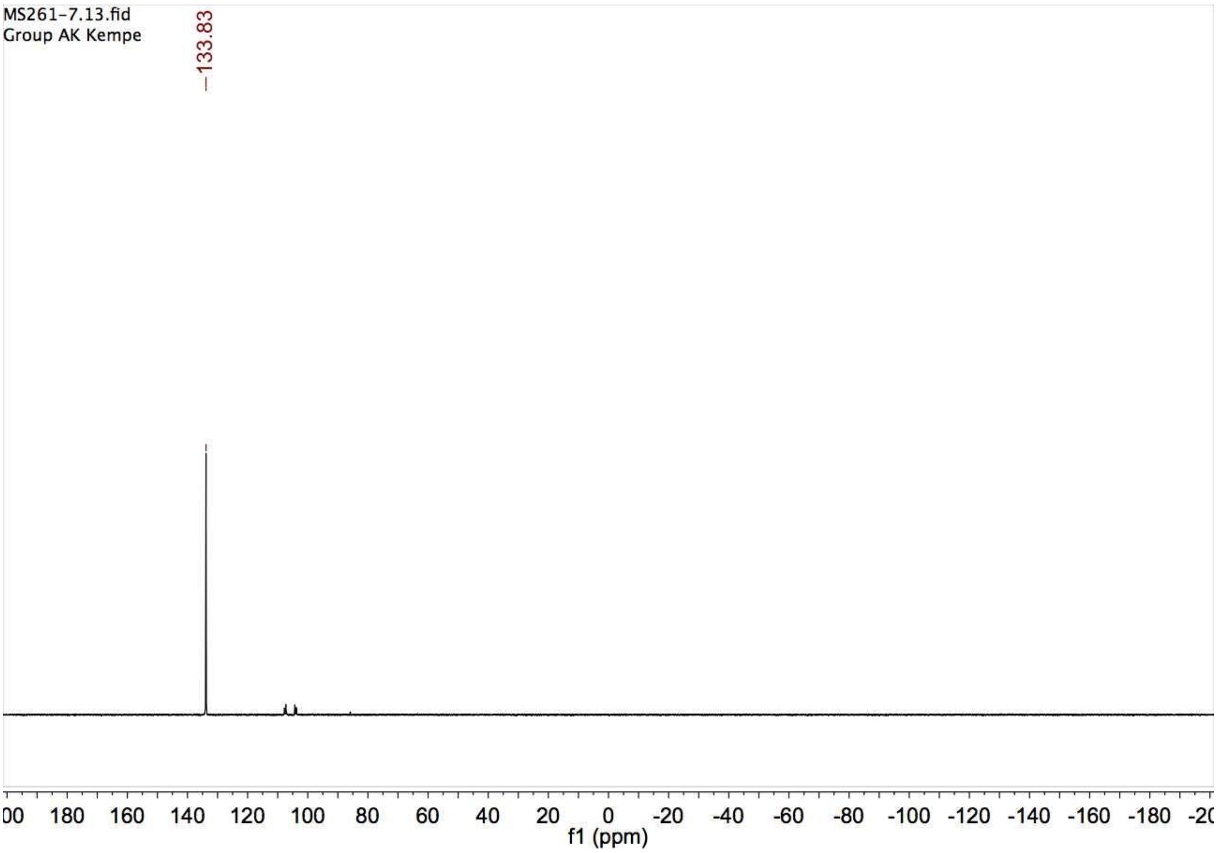
MS261-7.13.fid
Group AK Kempe
133.83
f1 (ppm)

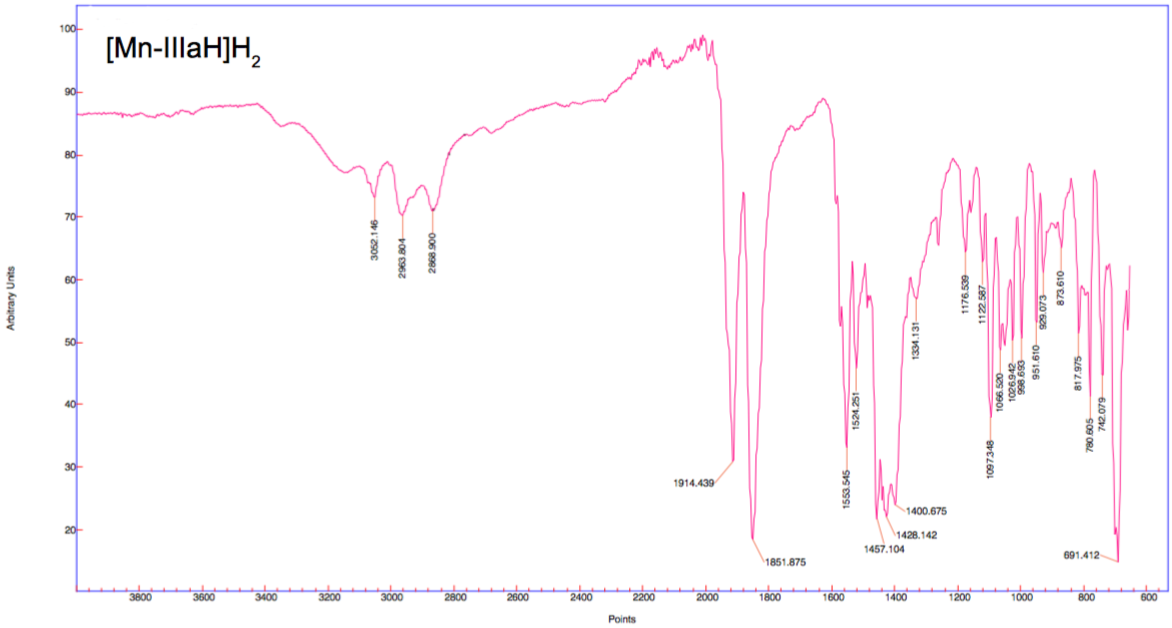
[Mn-IIIaH]H_2
Arbitrary Units
Points
3052.146
2963.804
2868.900
1914.439
1851.875
1553.545
1524.251
1457.104
1428.142
1400.675
1334.131
1176.539
1122.587
1097.348
1066.520
1026.942
998.693
951.610
929.073
873.610
817.975
780.605
742.079
691.412

[**Mn-IIIaH**]**K**$_2$ using KO^{t}Bu

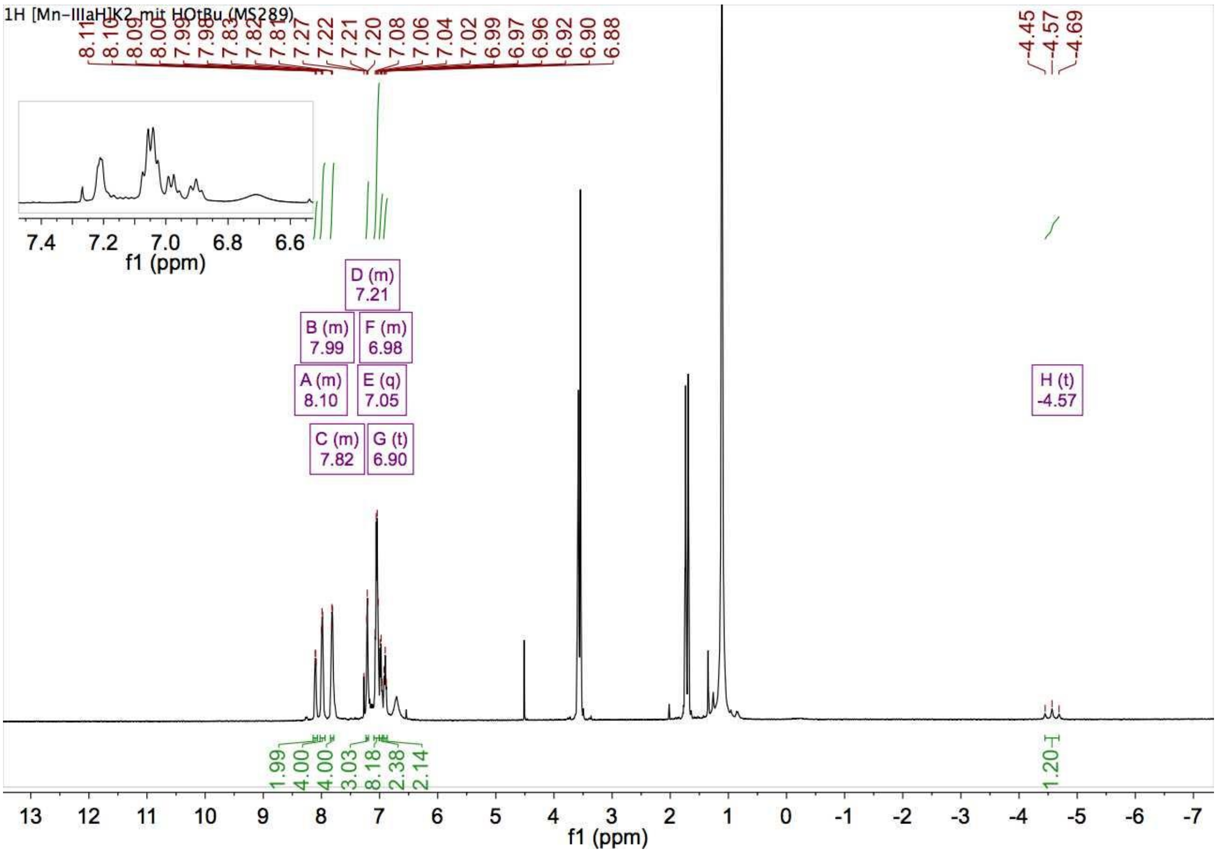

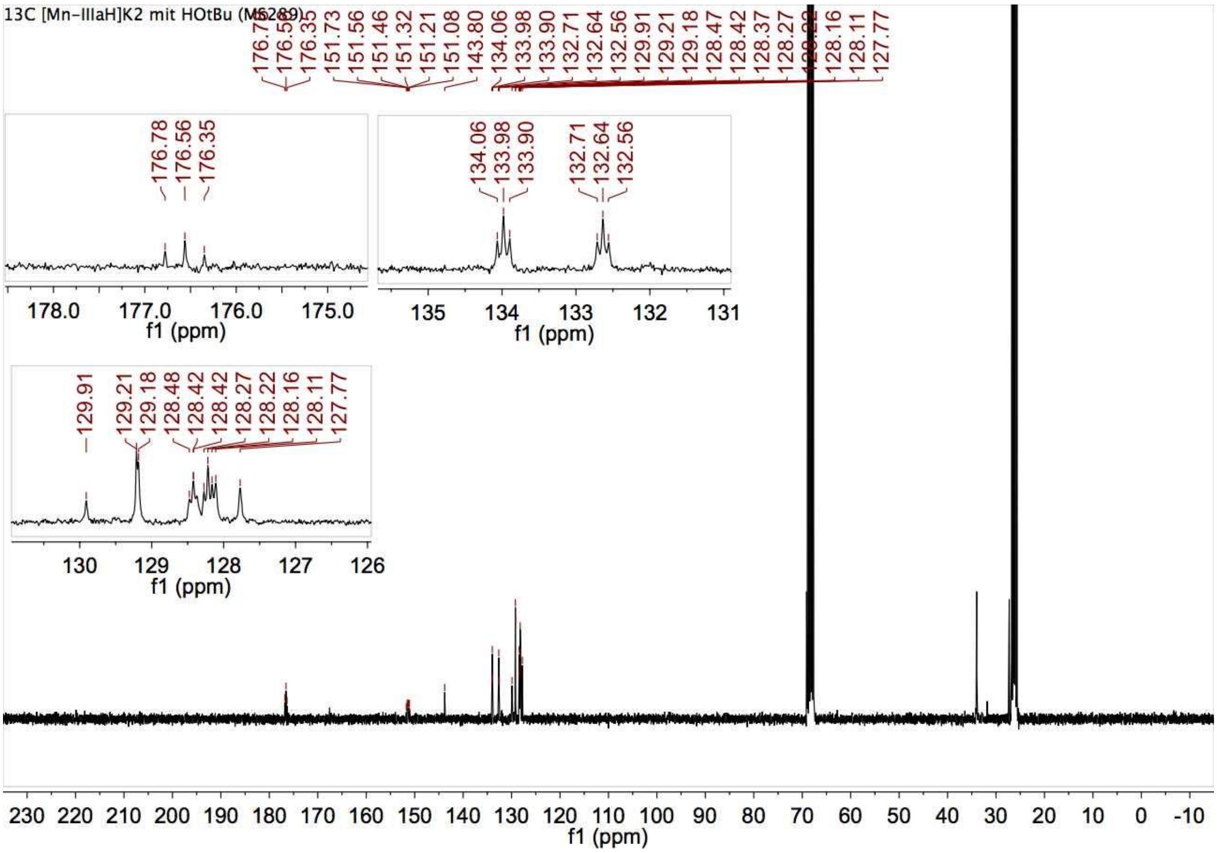

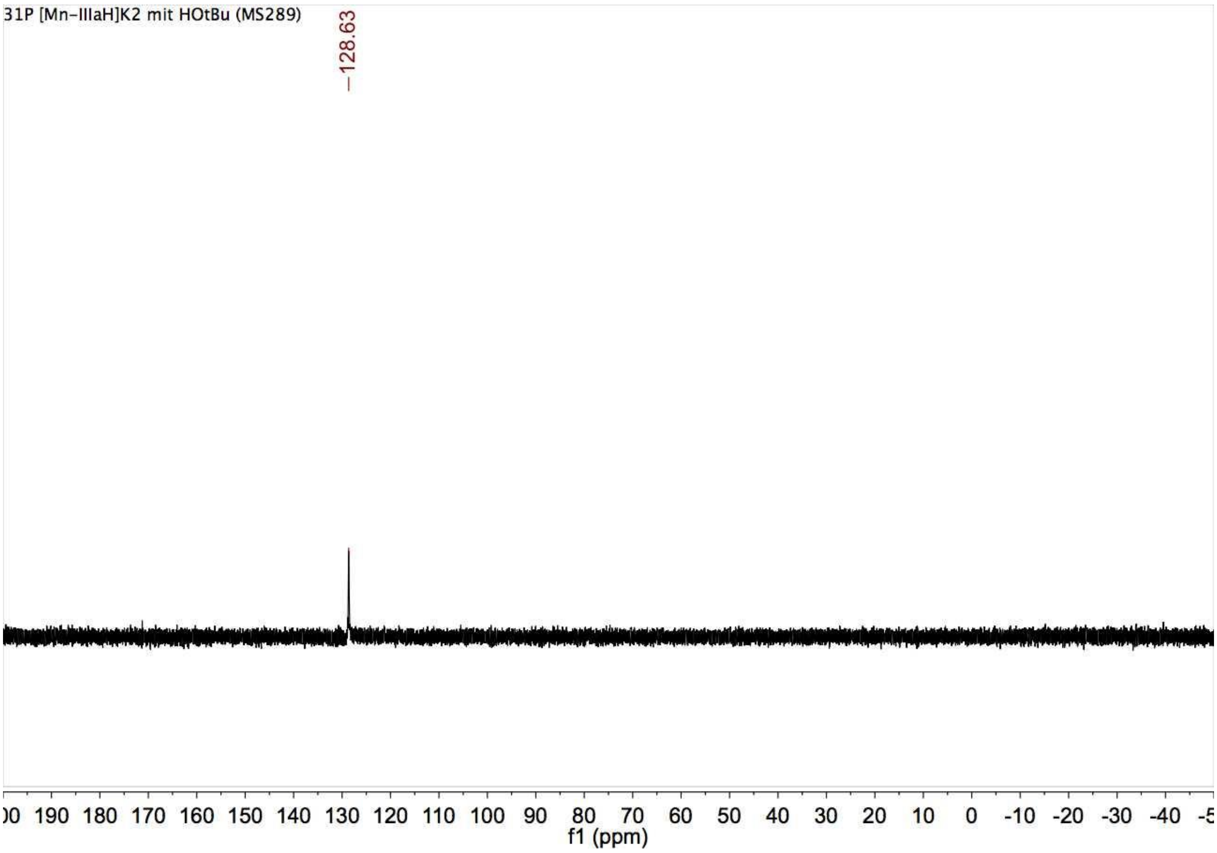
31P [Mn-IIIaH]K2 mit HOtBu (MS289)
128.63
f1 (ppm)

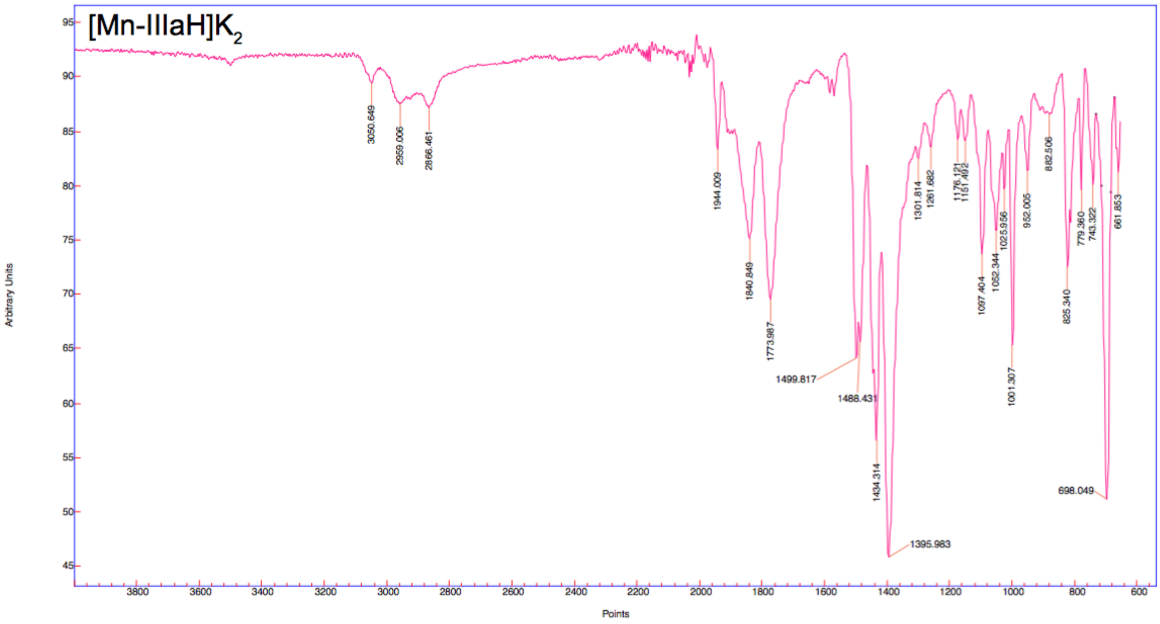
[Mn-IIIaH]K$_2$
Arbitrary Units
Points
3050.649
2959.006
2866.461
1944.009
1840.849
1773.987
1499.817
1488.431
1434.314
1395.983
1301.814
1261.682
1176.121
1151.492
1097.404
1052.344
1025.956
1001.307
952.005
882.506
825.340
779.360
743.322
698.049
661.853

[**Mn-IIIaH**]**K**$_2$ using KH

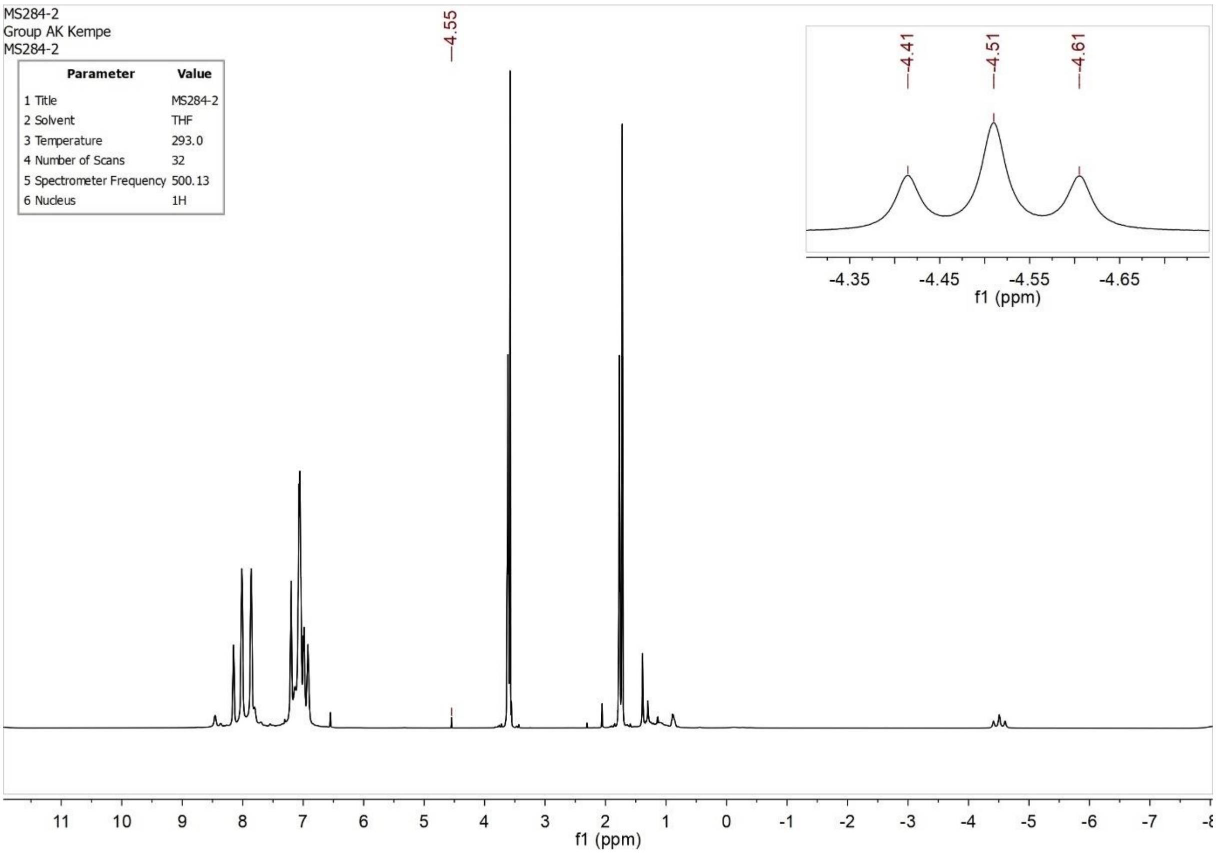

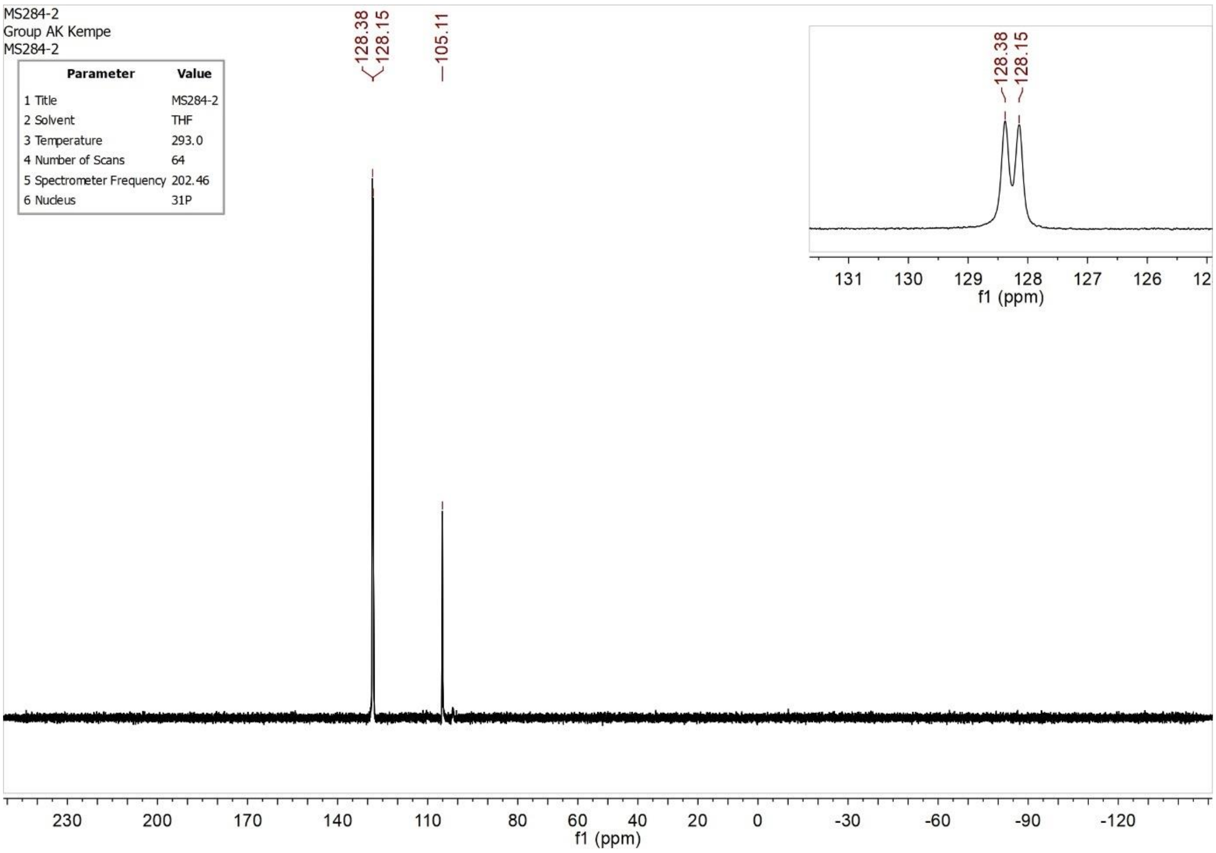

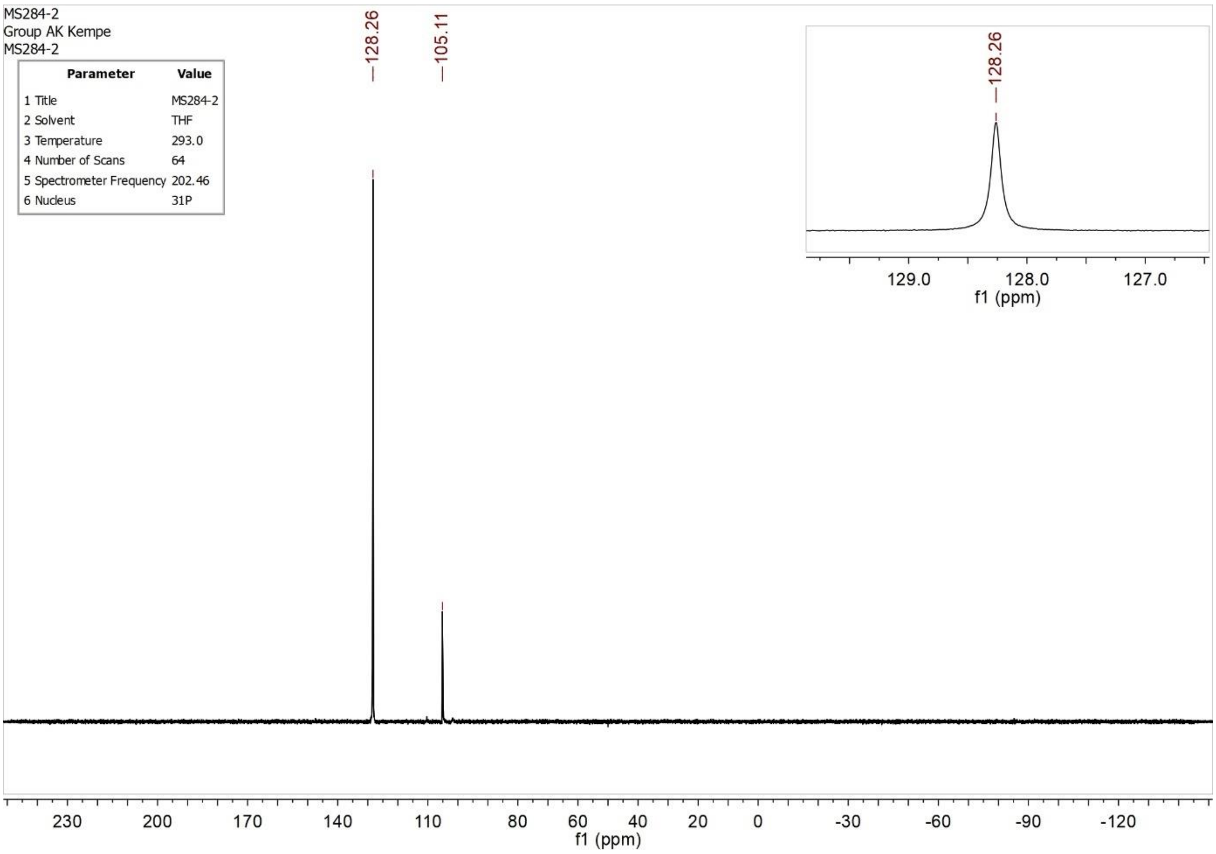
MS284-2
Group AK Kempe
MS284-2
Parameter Value
1 Title MS284-2
2 Solvent THF
3 Temperature 293.0
4 Number of Scans 64
5 Spectrometer Frequency 202.46
6 Nucleus 31P
128.26
105.11
f1 (ppm)

[Mn-IIIa]K

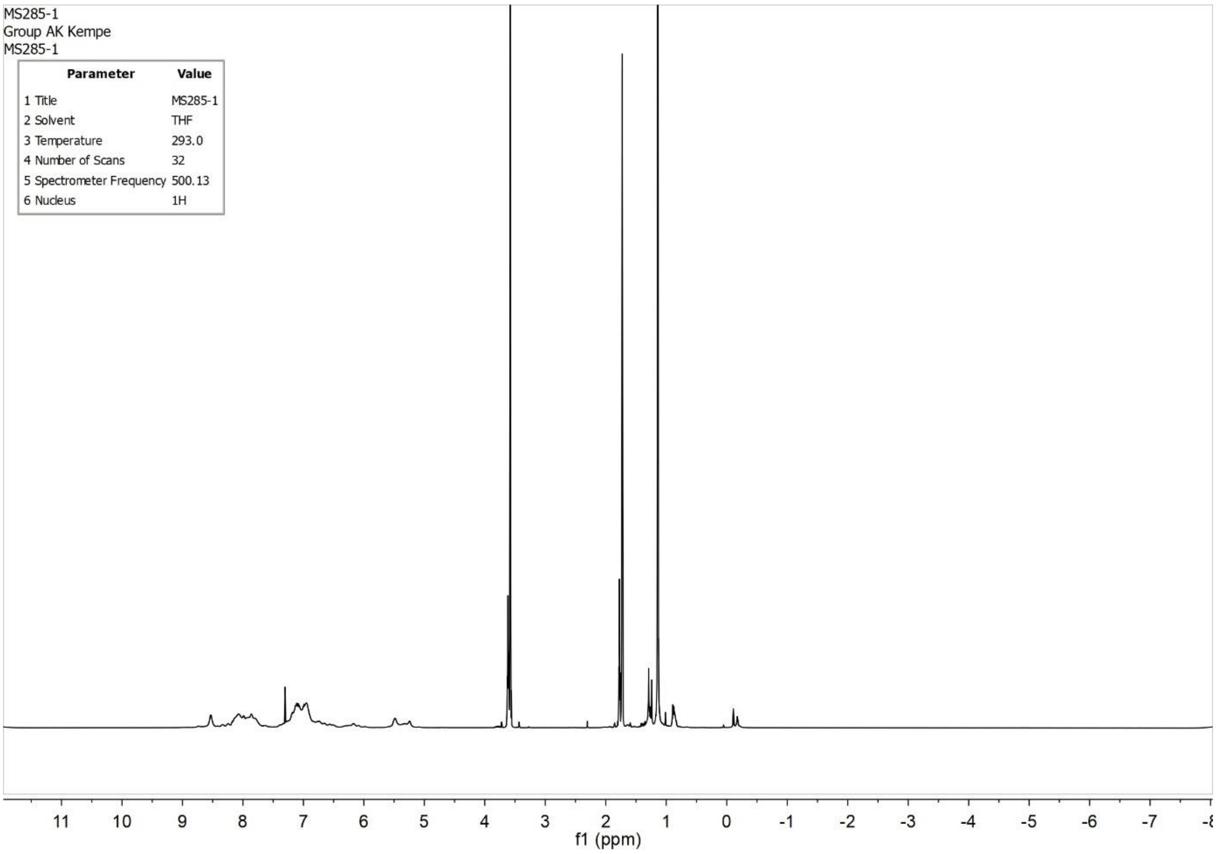

MS285-1
Group AK Kempe
MS285-1
Parameter Value
1 Title MS285-1
2 Solvent THF
3 Temperature 293.0
4 Number of Scans 32
5 Spectrometer Frequency 500.13
6 Nucleus 1H
11 10 9 8 7 6 5 4 3 2 1 0 -1 -2 -3 -4 -5 -6 -7
f1 (ppm)

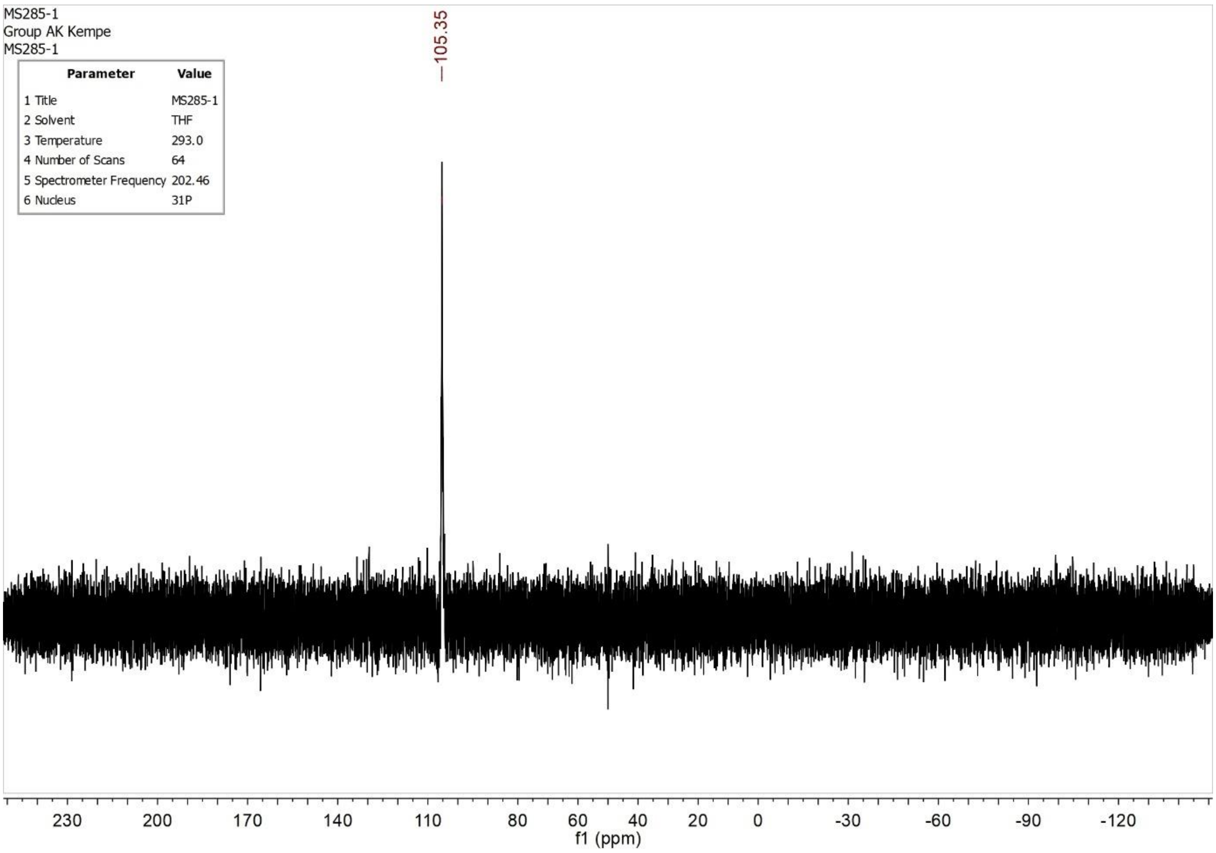

MS285-1
Group AK Kempe
MS285-1
105.35
Parameter Value
1 Title MS285-1
2 Solvent THF
3 Temperature 293.0
4 Number of Scans 64
5 Spectrometer Frequency 202.46
6 Nucleus 31P
230 200 170 140 110 80 60 40 20 0 -30 -60 -90 -120
f1 (ppm)

NMR-spectra of mechanistic control experiments

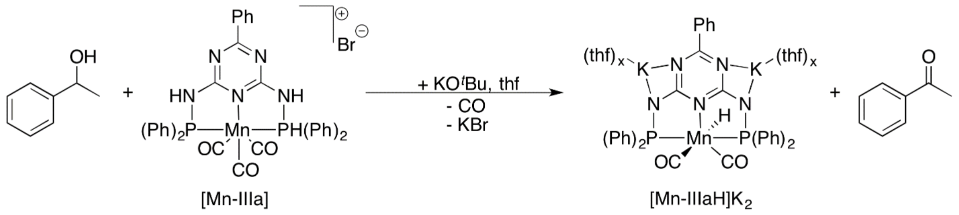

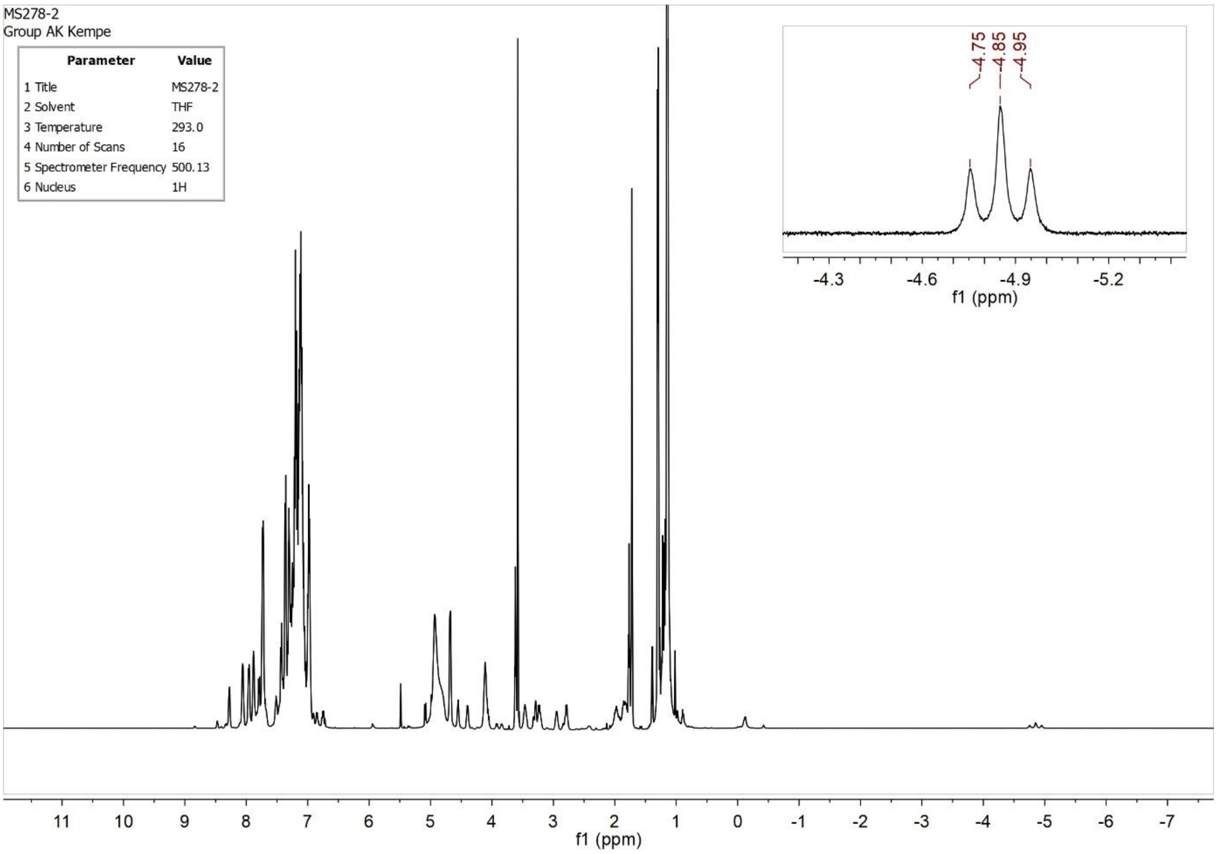

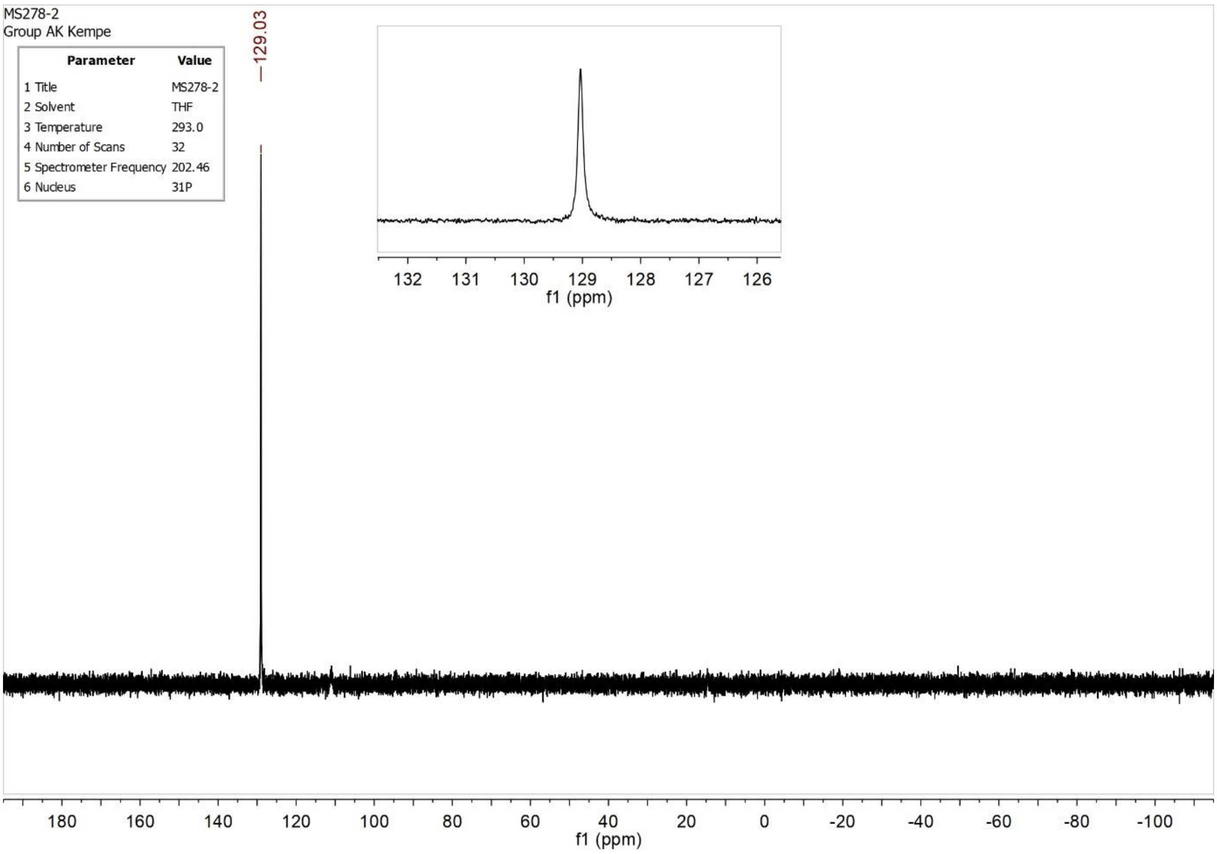
MS278-2
Group AK Kempe
—129.03
Parameter	Value
1 Title	MS278-2
2 Solvent	THF
3 Temperature	293.0
4 Number of Scans	32
5 Spectrometer Frequency	202.46
6 Nucleus	31P
132 131 130 129 128 127 126
f1 (ppm)
180 160 140 120 100 80 60 40 20 0 -20 -40 -60 -80 -100
f1 (ppm)

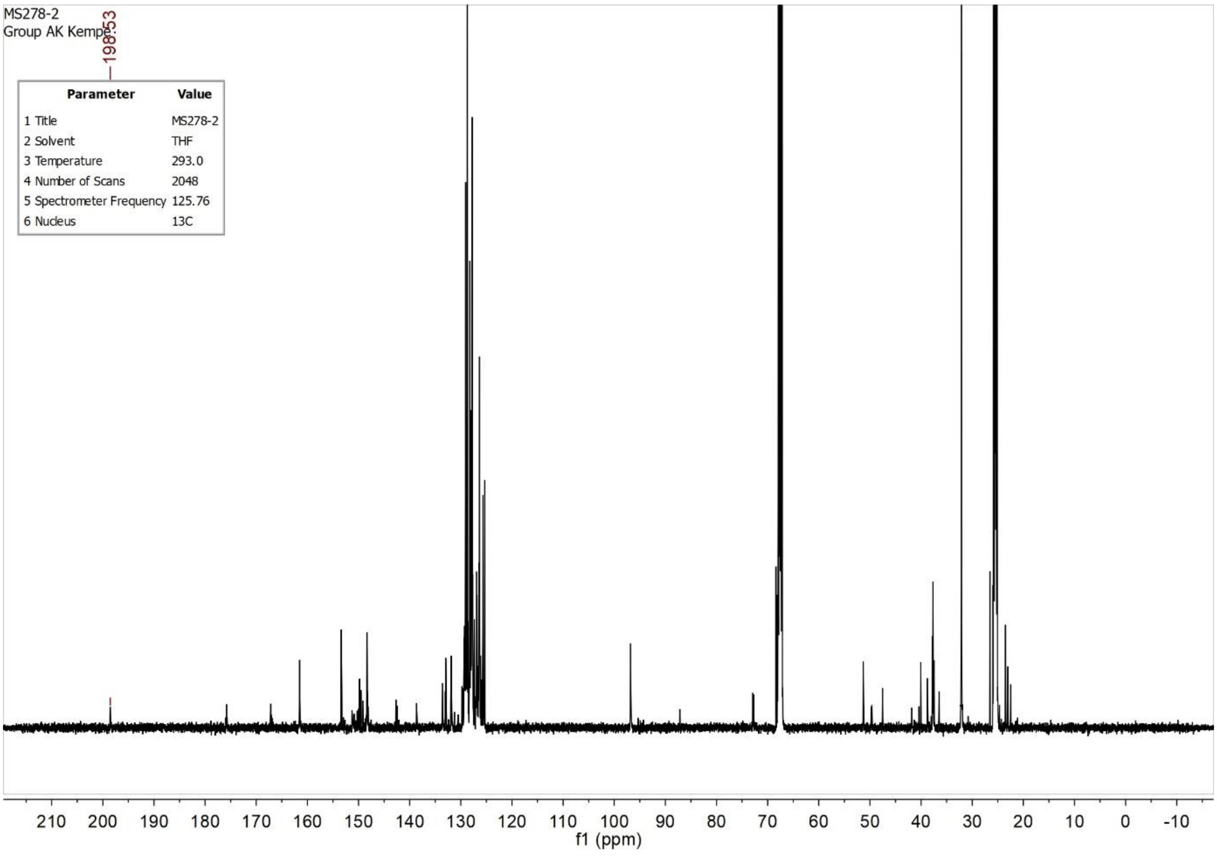
MS278-2
Group AK Kempe
—198.53
Parameter	Value
1 Title	MS278-2
2 Solvent	THF
3 Temperature	293.0
4 Number of Scans	2048
5 Spectrometer Frequency	125.76
6 Nucleus	13C
210 200 190 180 170 160 150 140 130 120 110 100 90 80 70 60 50 40 30 20 10 0 -10
f1 (ppm)

NMR-spectra of temperature studies with [Mn-IIIaH]K_2

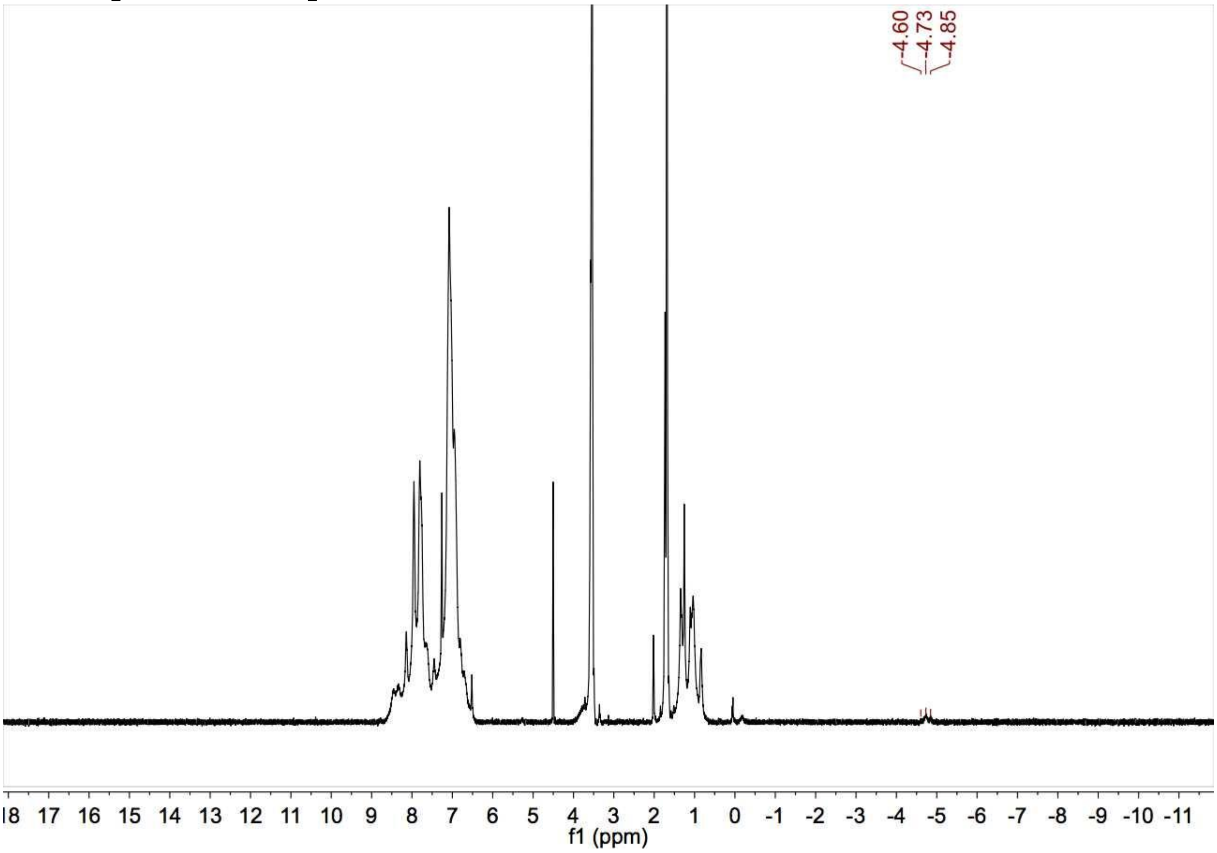

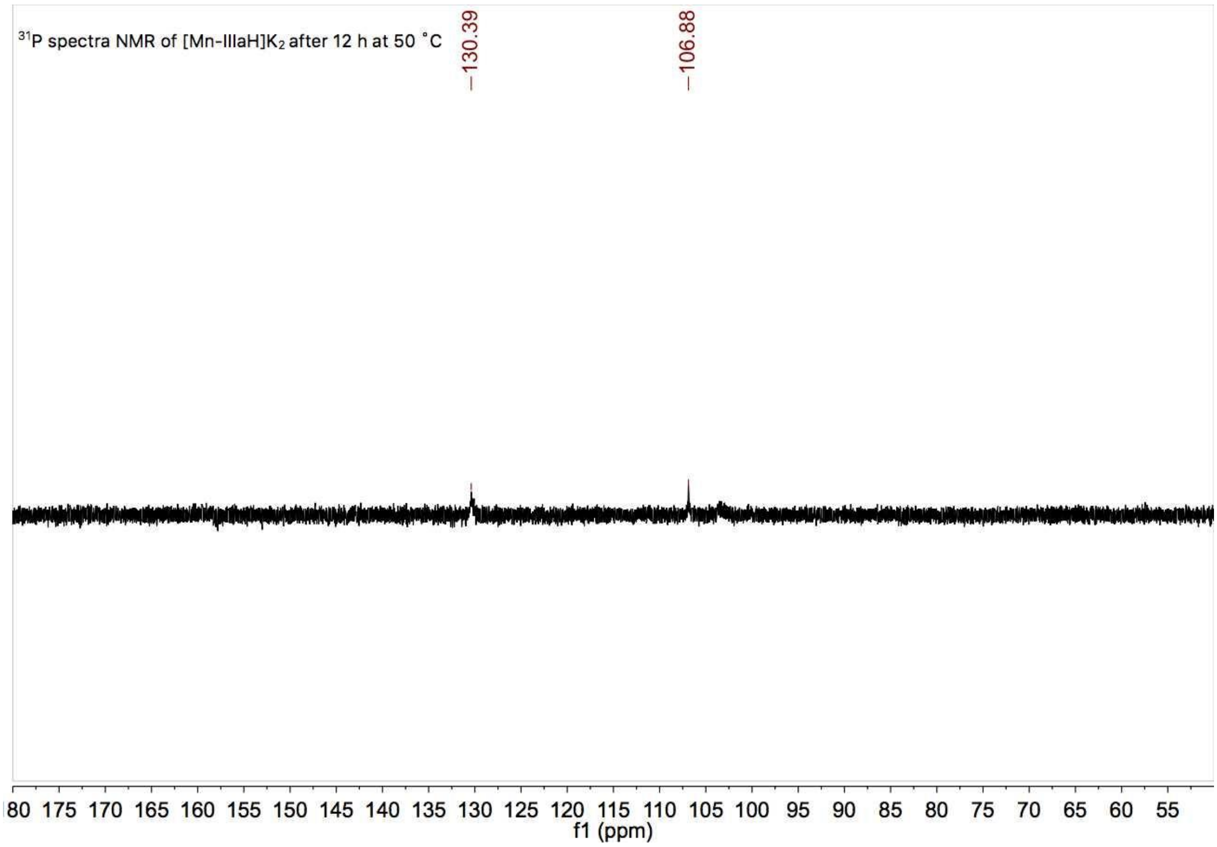

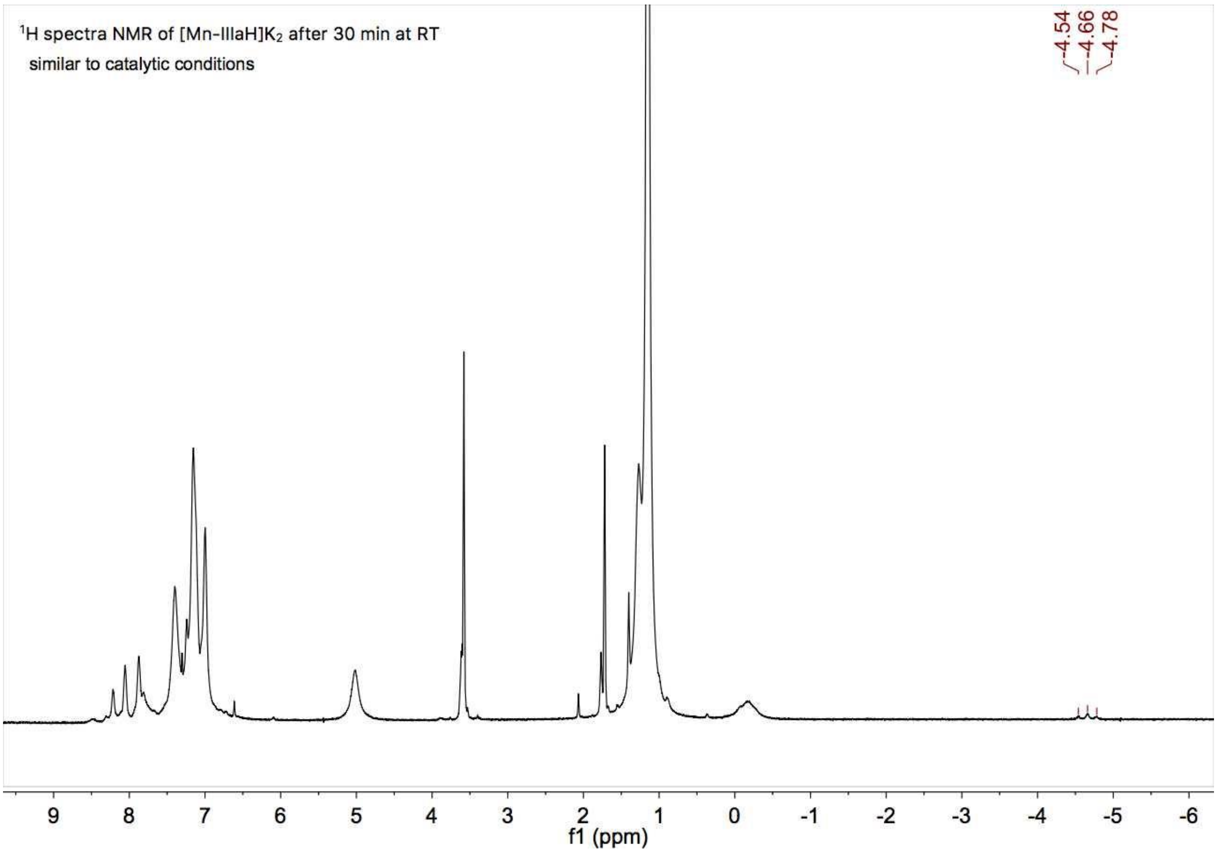

^{1}H spectra NMR of [Mn-IIIaH]K_2 after 30 min at RT
similar to catalytic conditions
-4.54
-4.66
-4.78
9 8 7 6 5 4 3 2 1 0 -1 -2 -3 -4 -5 -6
f1 (ppm)

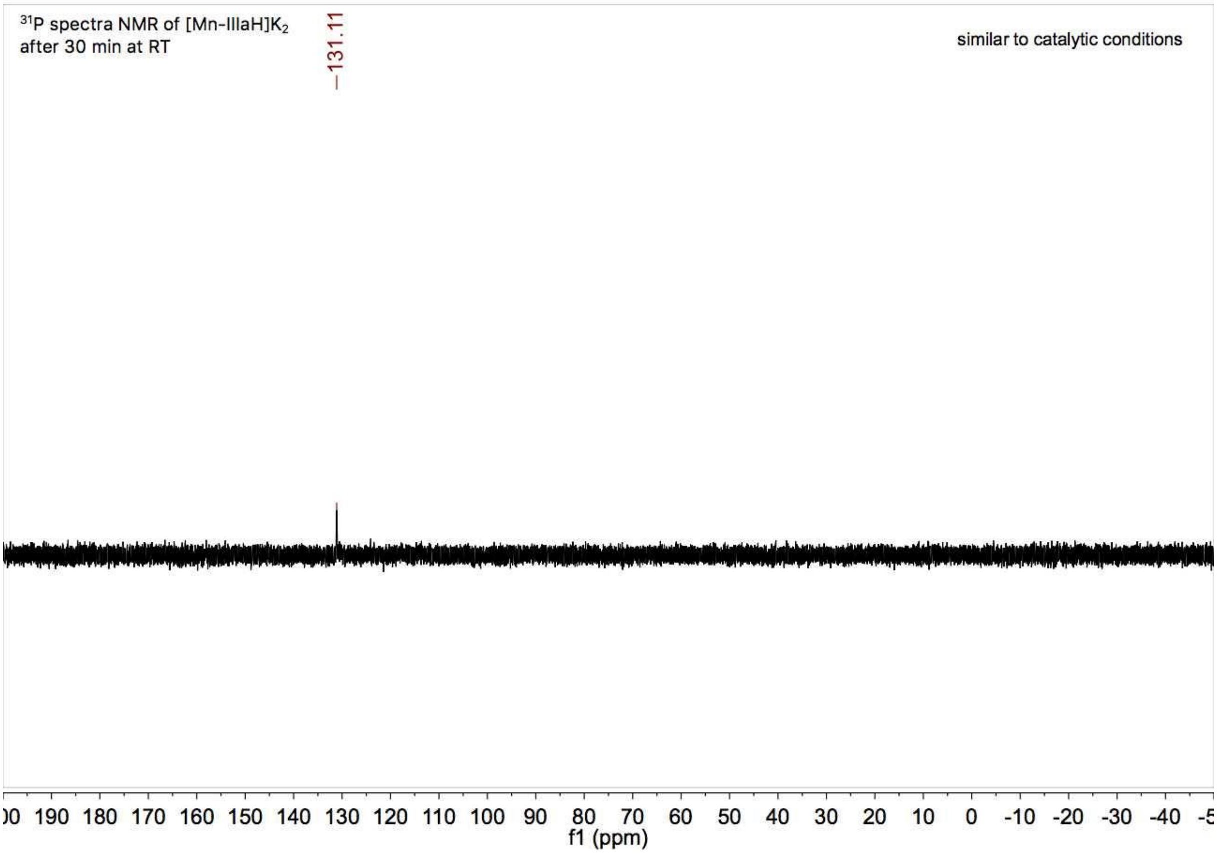

^{31}P spectra NMR of [Mn-IIIaH]K_2
after 30 min at RT
-131.11
similar to catalytic conditions
190 180 170 160 150 140 130 120 110 100 90 80 70 60 50 40 30 20 10 0 -10 -20 -30 -40
f1 (ppm)

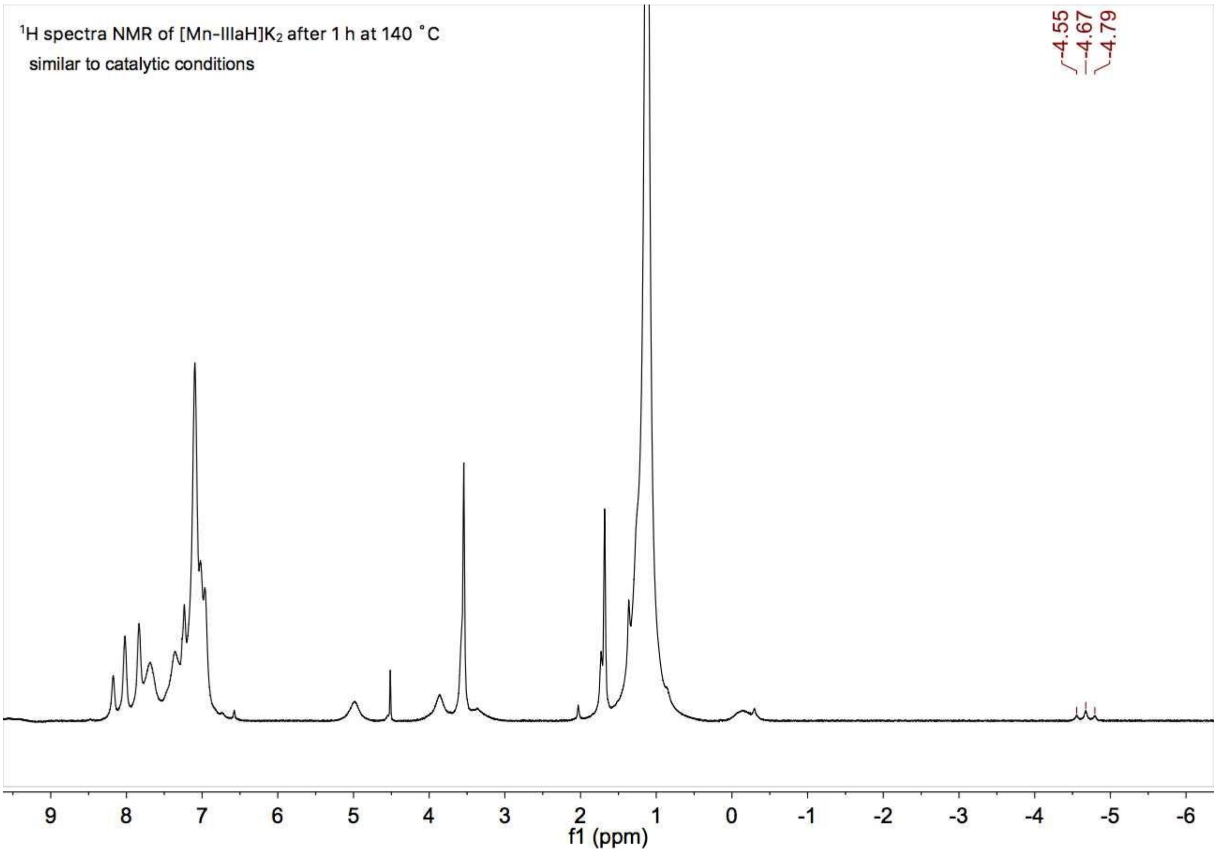

^{1}H spectra NMR of [Mn-IIIaH]K_2 after 1 h at 140 ˚C
similar to catalytic conditions
-4.55
-4.67
-4.79
9 8 7 6 5 4 3 2 1 0 -1 -2 -3 -4 -5 -6
f1 (ppm)

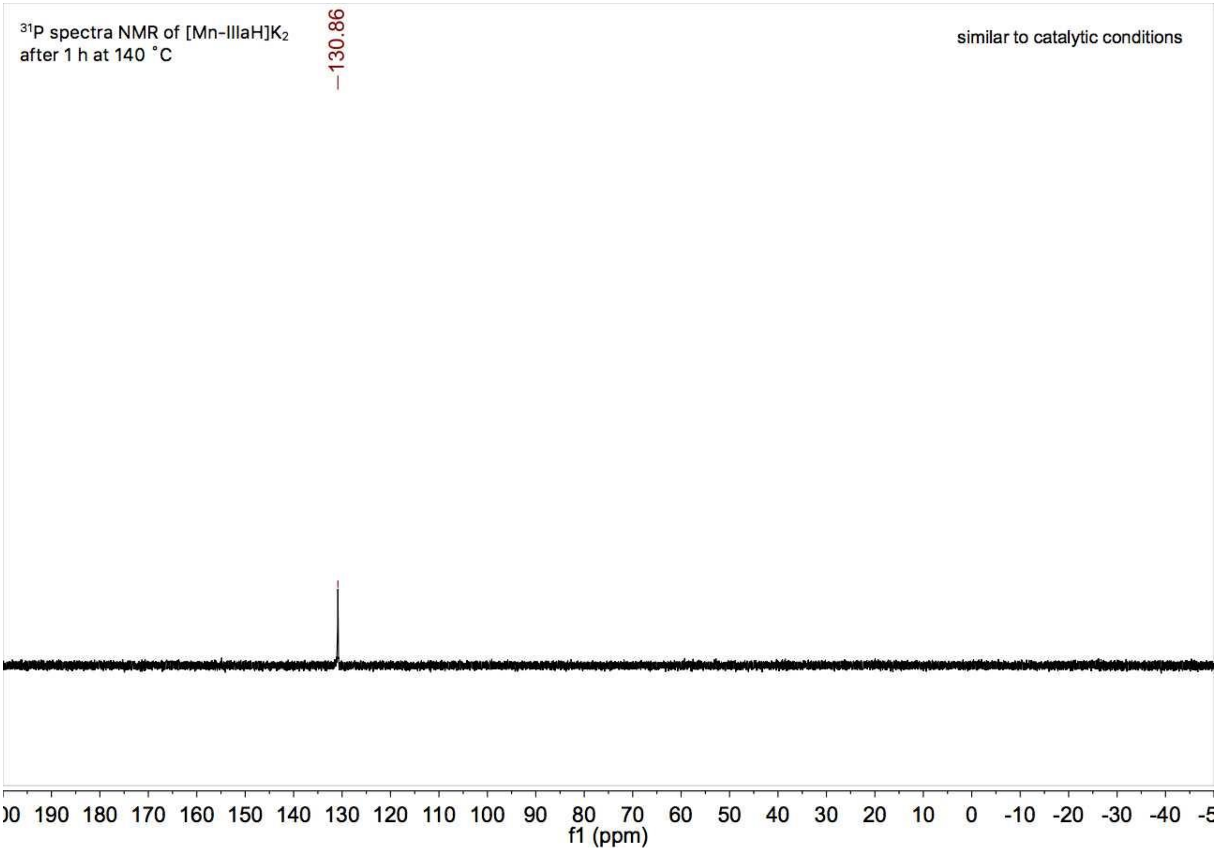

^{31}P spectra NMR of [Mn-IIIaH]K_2
after 1 h at 140 ˚C
130.86
similar to catalytic conditions
190 180 170 160 150 140 130 120 110 100 90 80 70 60 50 40 30 20 10 0 -10 -20 -30 -40
f1 (ppm)

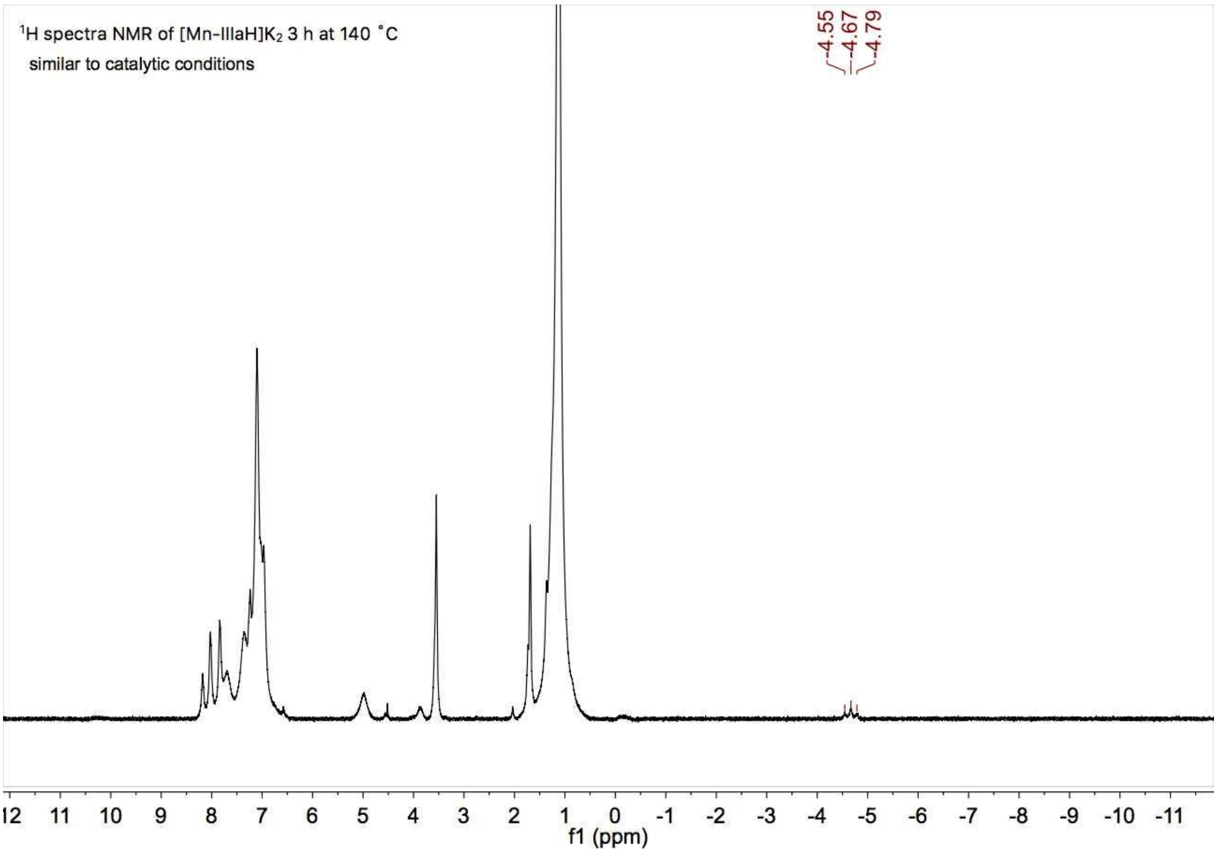

^{1}H spectra NMR of [Mn-IIIaH]K_2 3 h at 140 ˚C
similar to catalytic conditions
-4.55
-4.67
-4.79
12 11 10 9 8 7 6 5 4 3 2 1 0 -1 -2 -3 -4 -5 -6 -7 -8 -9 -10 -11
f1 (ppm)

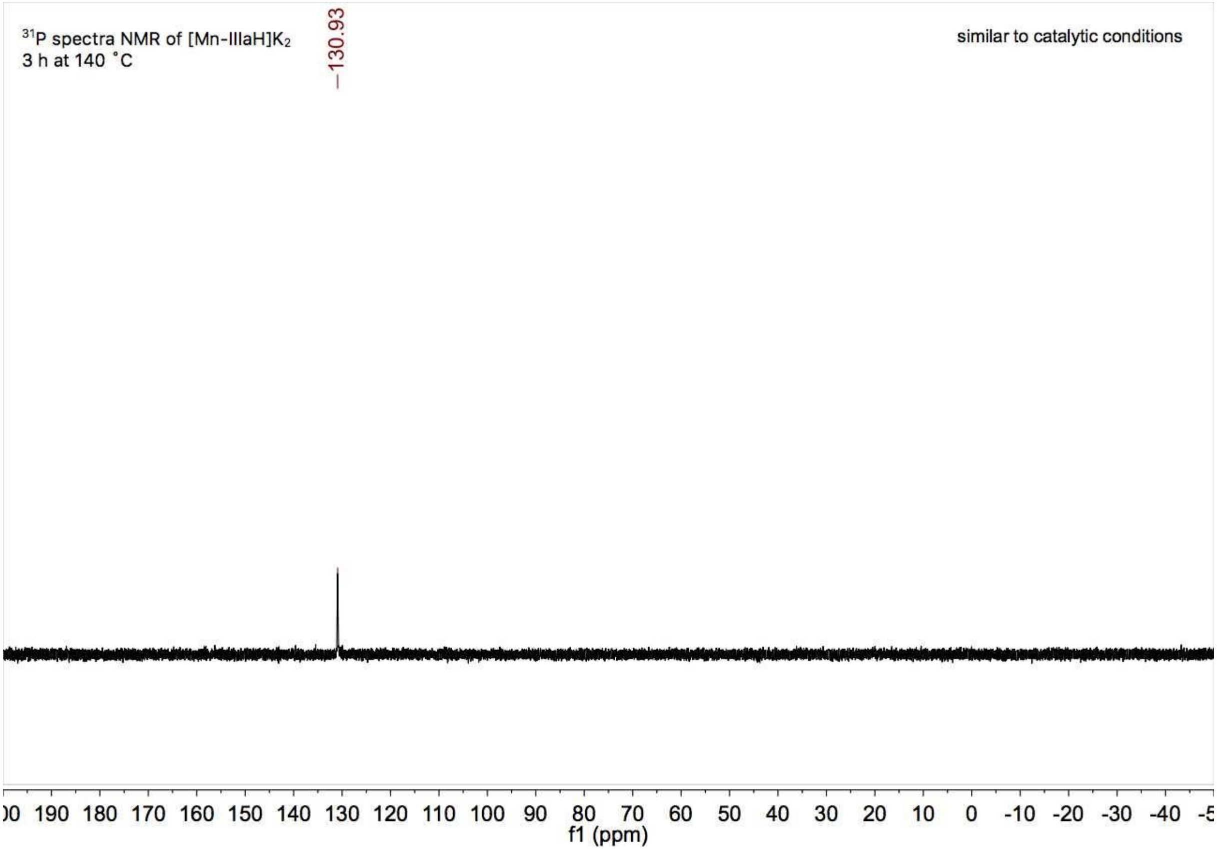

^{31}P spectra NMR of [Mn-IIIaH]K_2
3 h at 140 ˚C
130.93
similar to catalytic conditions
190 180 170 160 150 140 130 120 110 100 90 80 70 60 50 40 30 20 10 0 -10 -20 -30 -40
f1 (ppm)

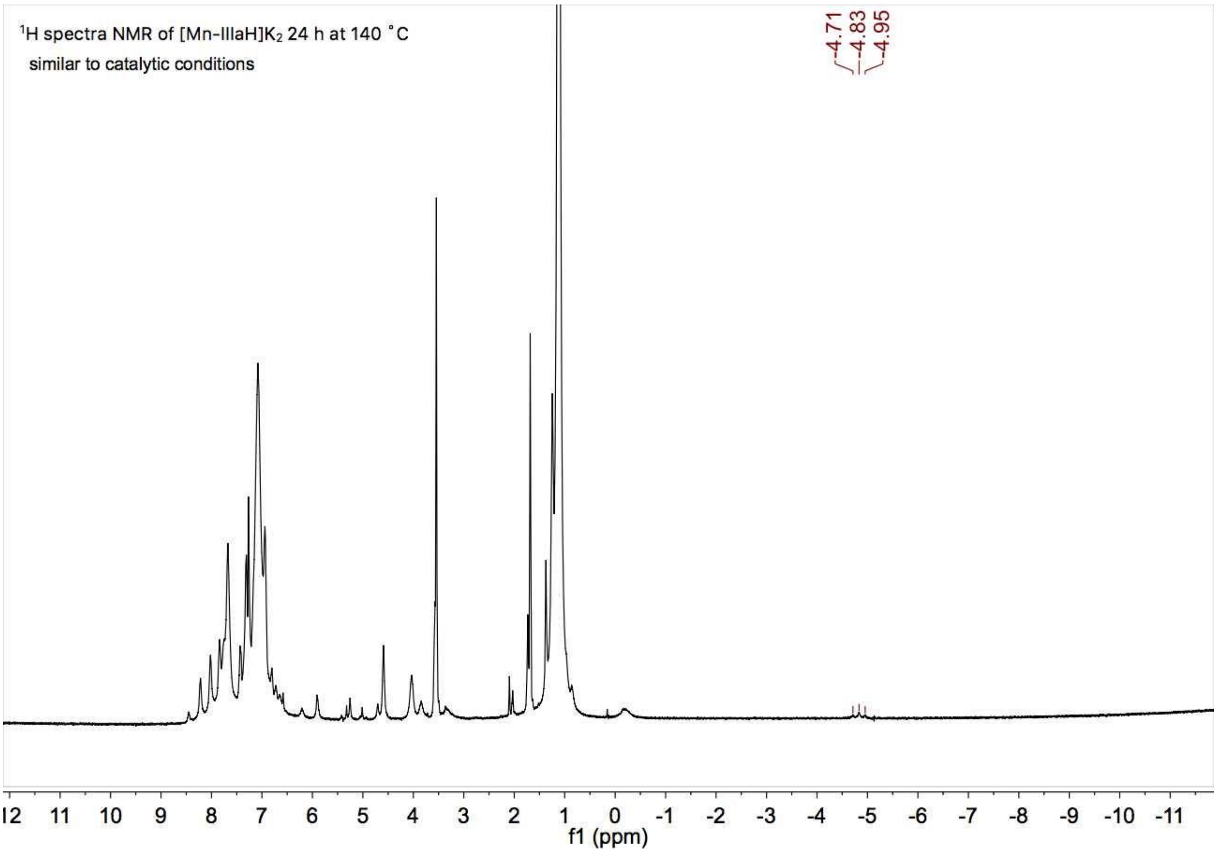

^{1}H spectra NMR of [Mn-IIIaH]K_2 24 h at 140 ˚C
similar to catalytic conditions
-4.71
-4.83
-4.95
12 11 10 9 8 7 6 5 4 3 2 1 0 -1 -2 -3 -4 -5 -6 -7 -8 -9 -10 -11
f1 (ppm)

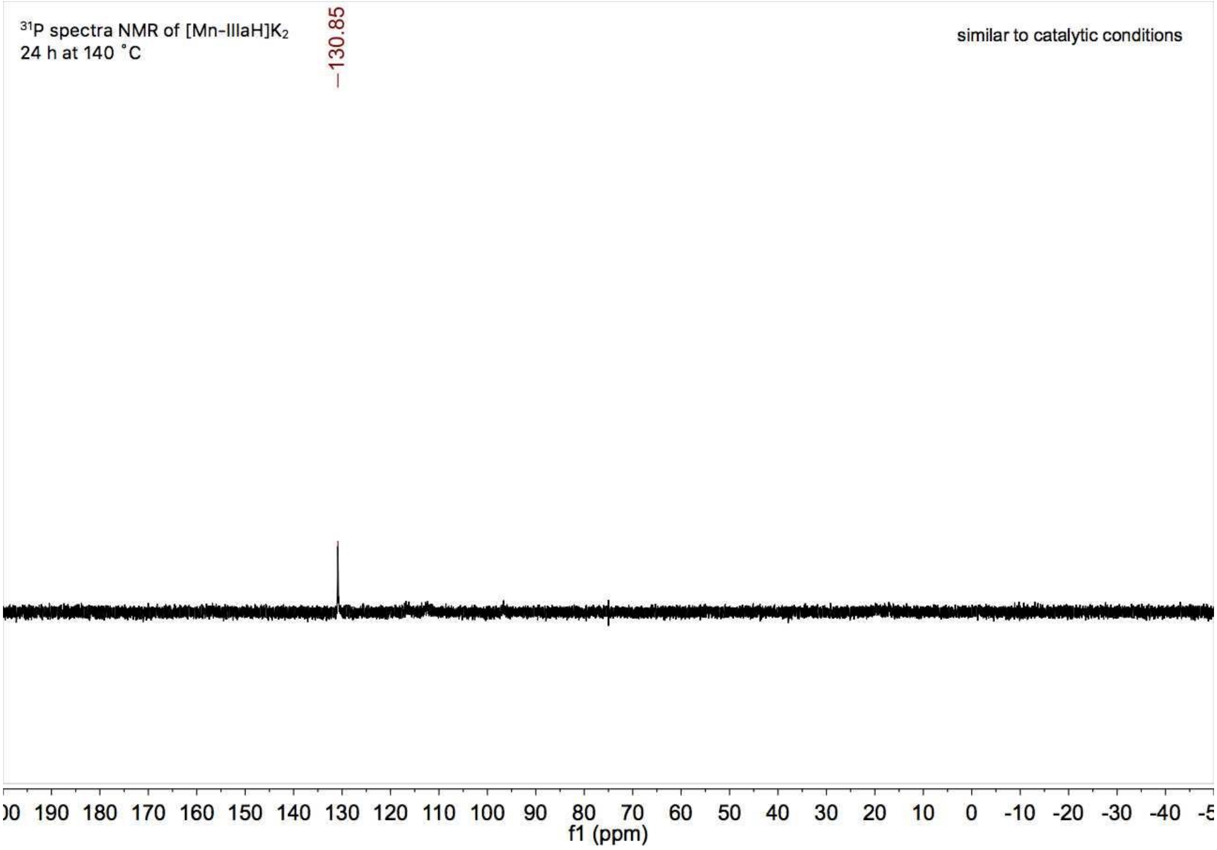

^{31}P spectra NMR of [Mn-IIIaH]K_2
24 h at 140 ˚C
130.85
similar to catalytic conditions
190 180 170 160 150 140 130 120 110 100 90 80 70 60 50 40 30 20 10 0 -10 -20 -30 -40
f1 (ppm)

NMR/MS-spectra

2-methyl-1-phenylpropan-1-ol (B1 / B19)

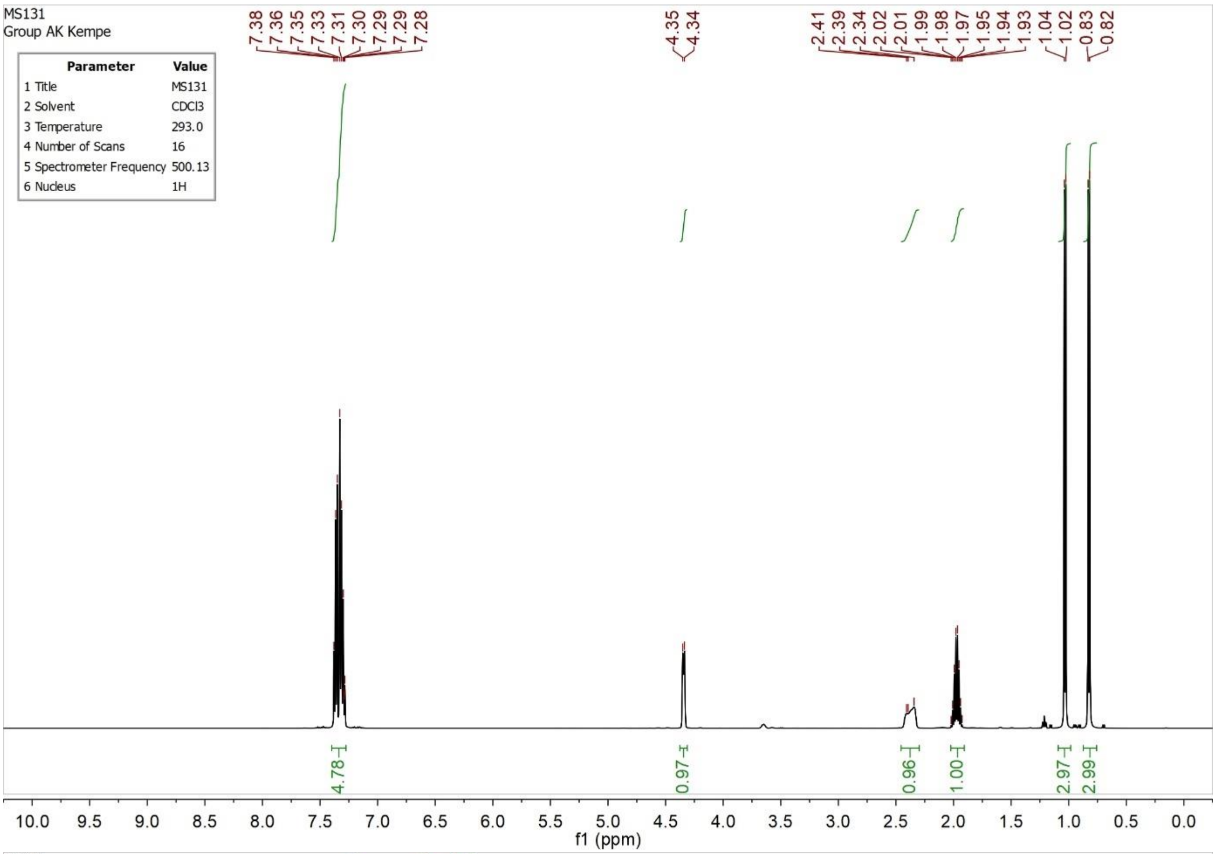

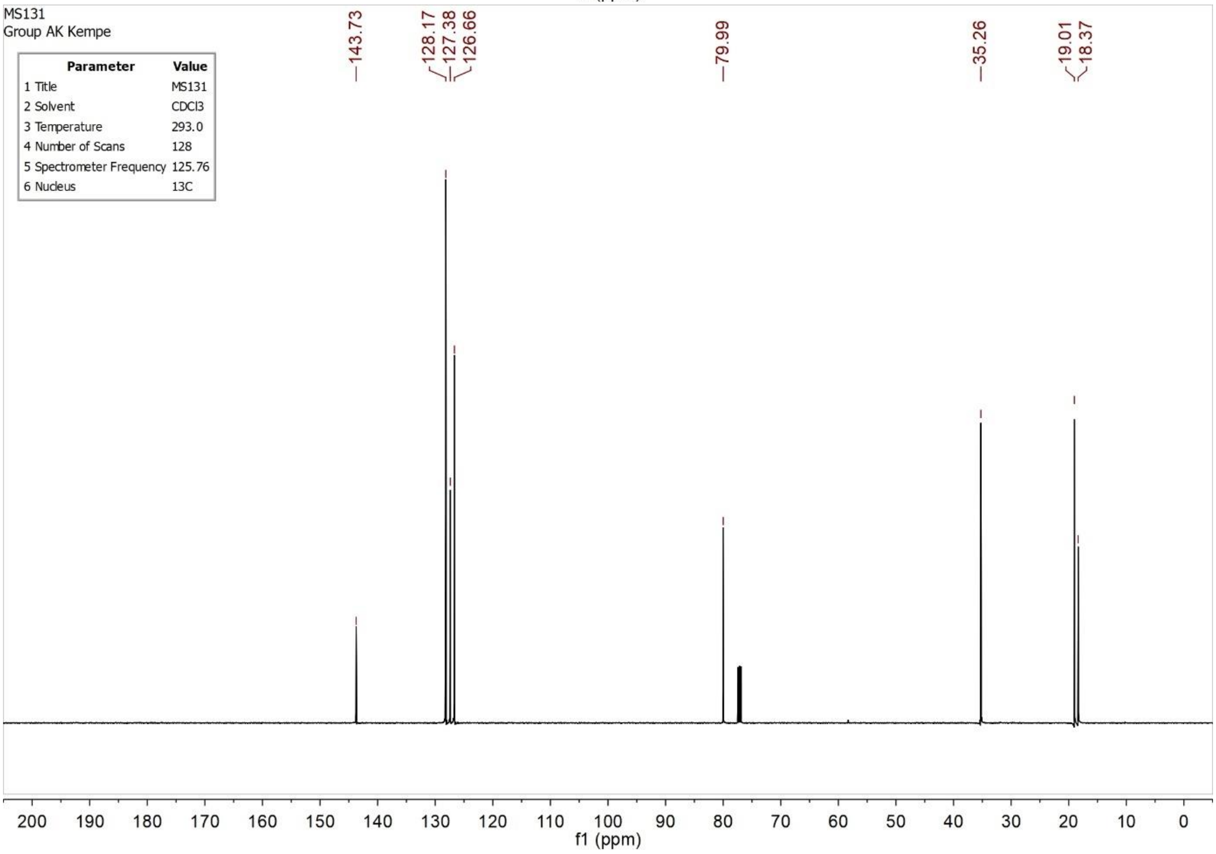

1-(2-methoxyphenyl)-2-methylpropan-1-ol (B2)

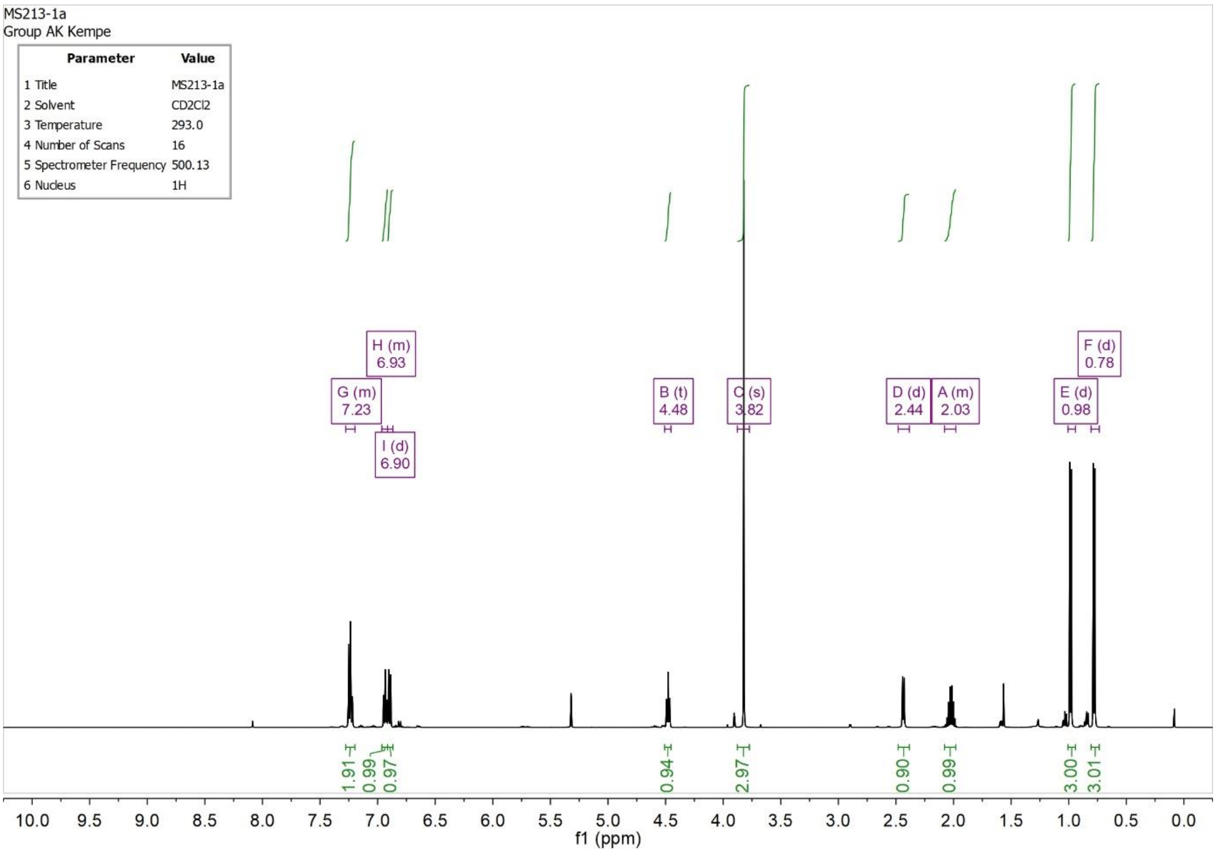

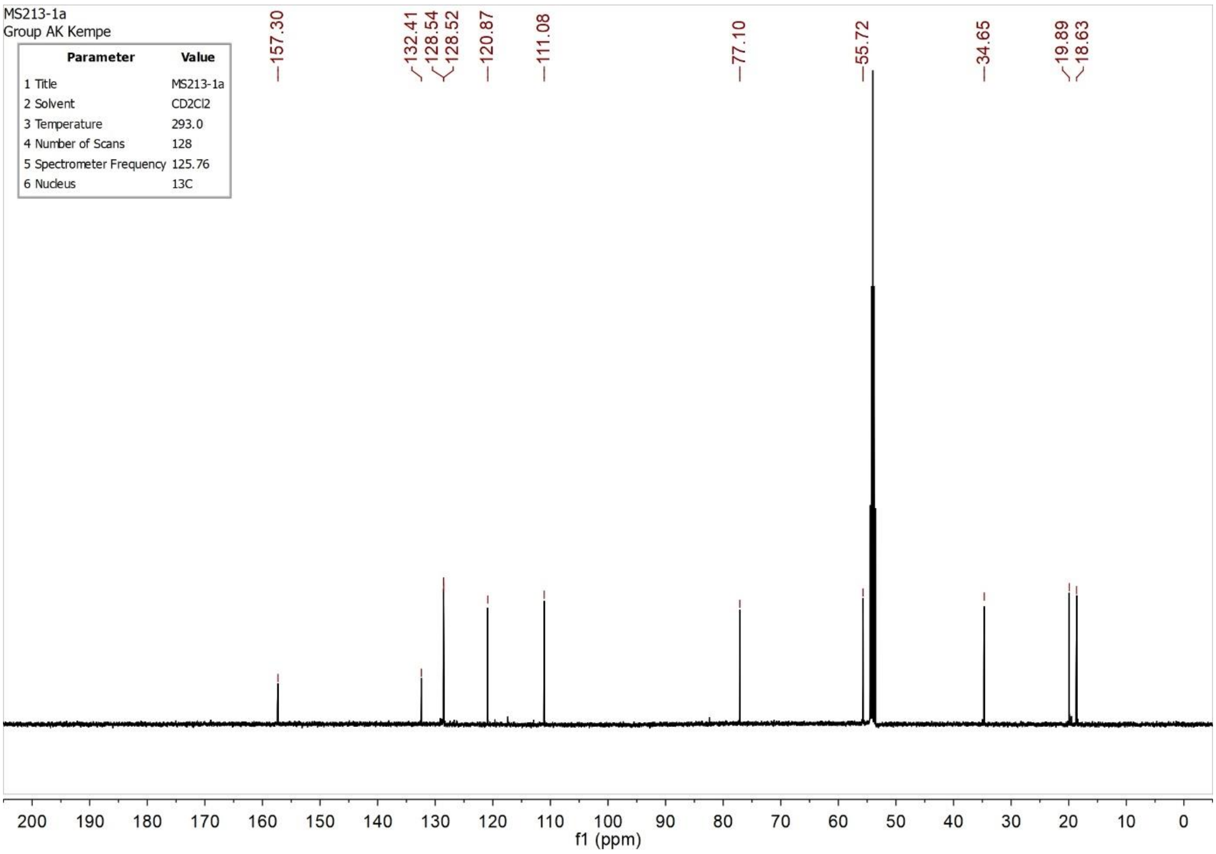

1-(3-methoxyphenyl)-2-methylpropan-1-ol (B3)

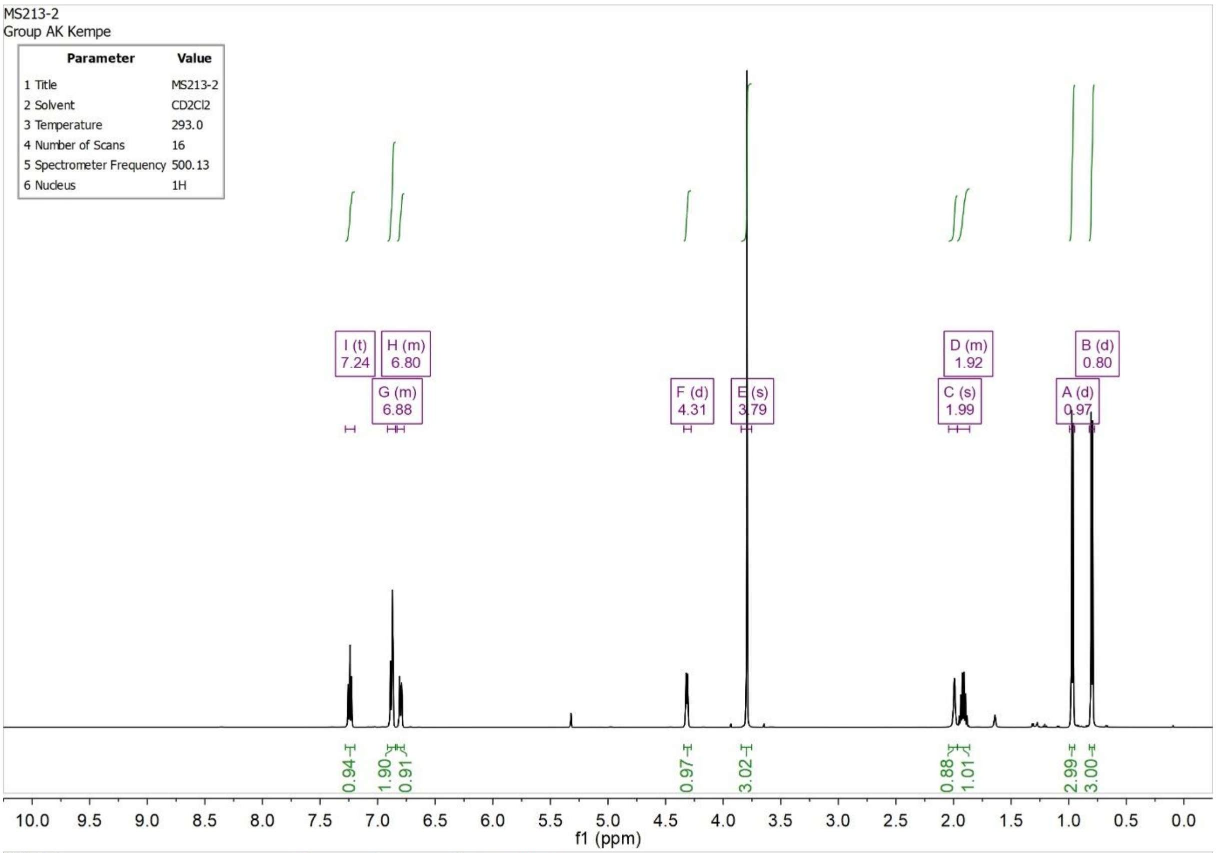

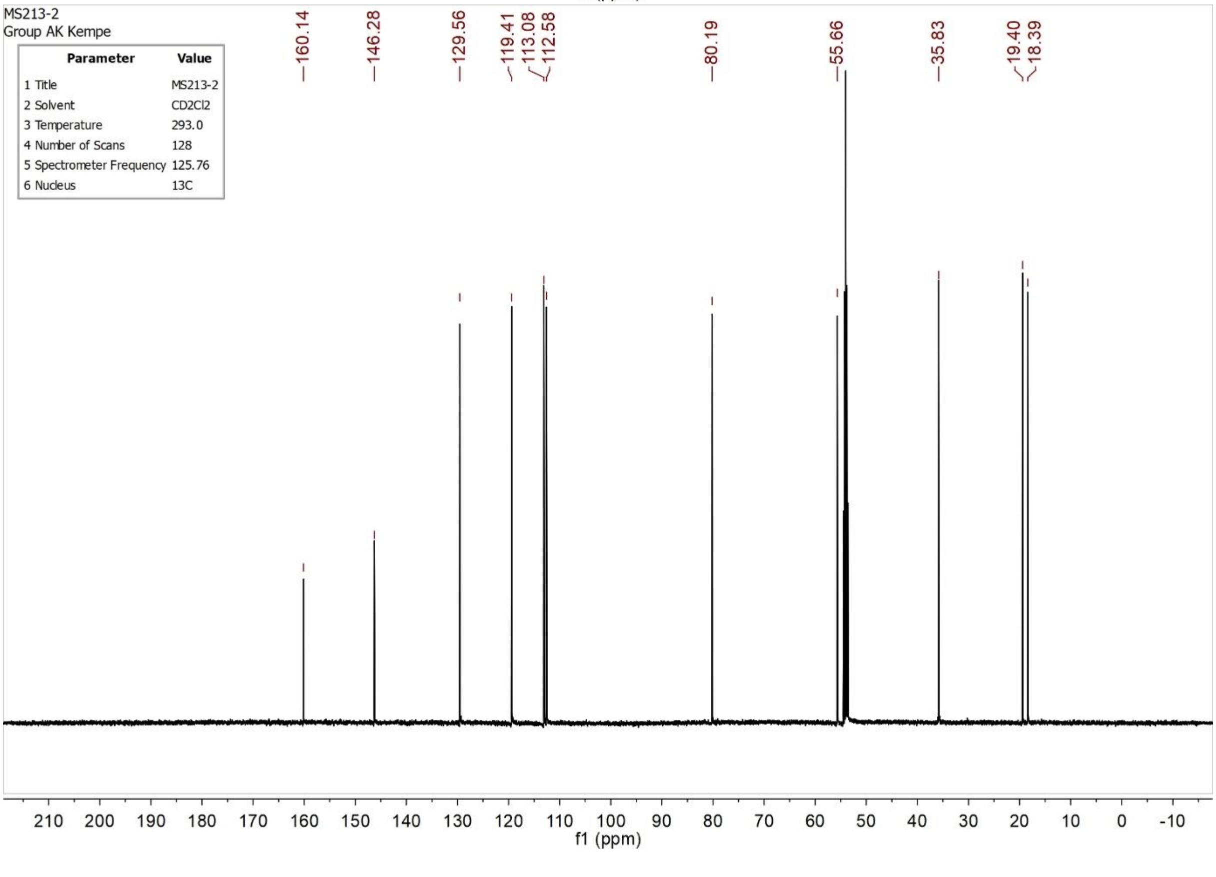

1-(4-methoxyphenyl)-2-methylpropan-1-ol (B4 / B20)

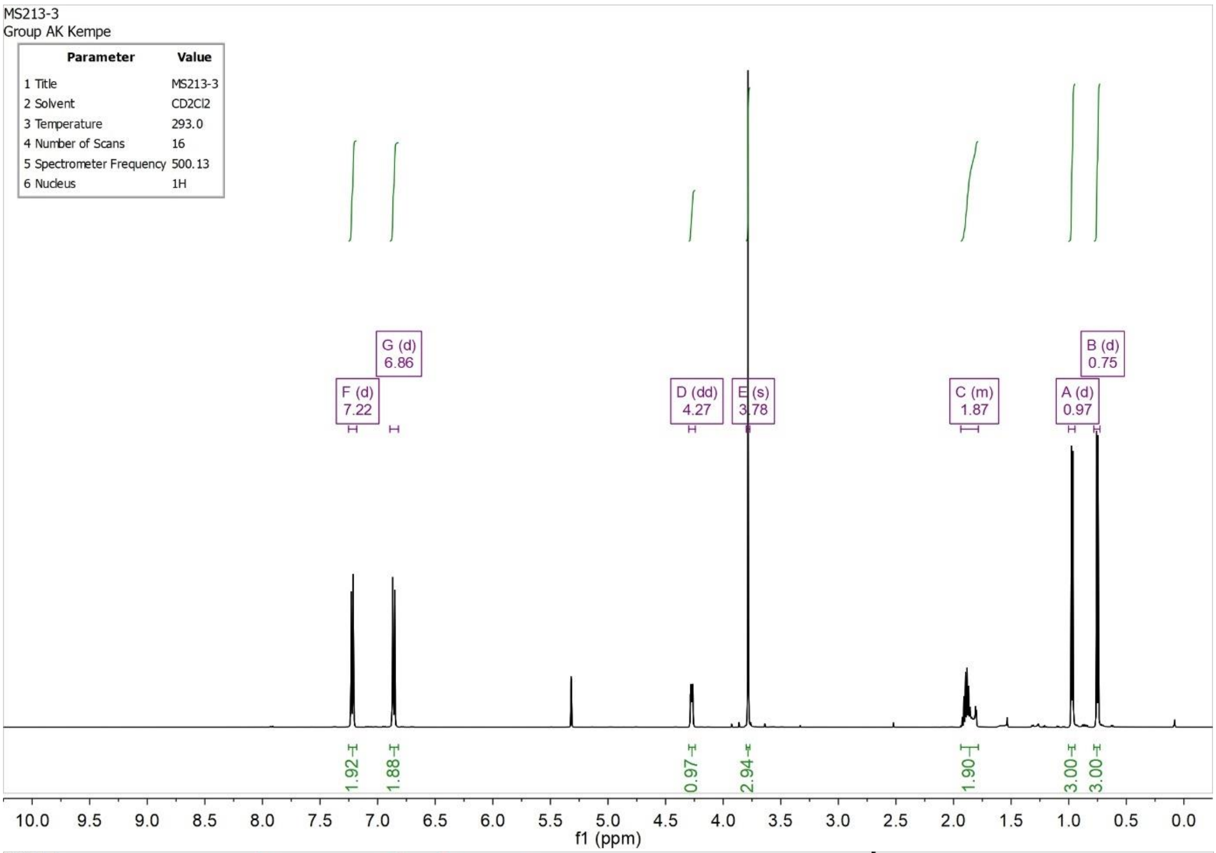

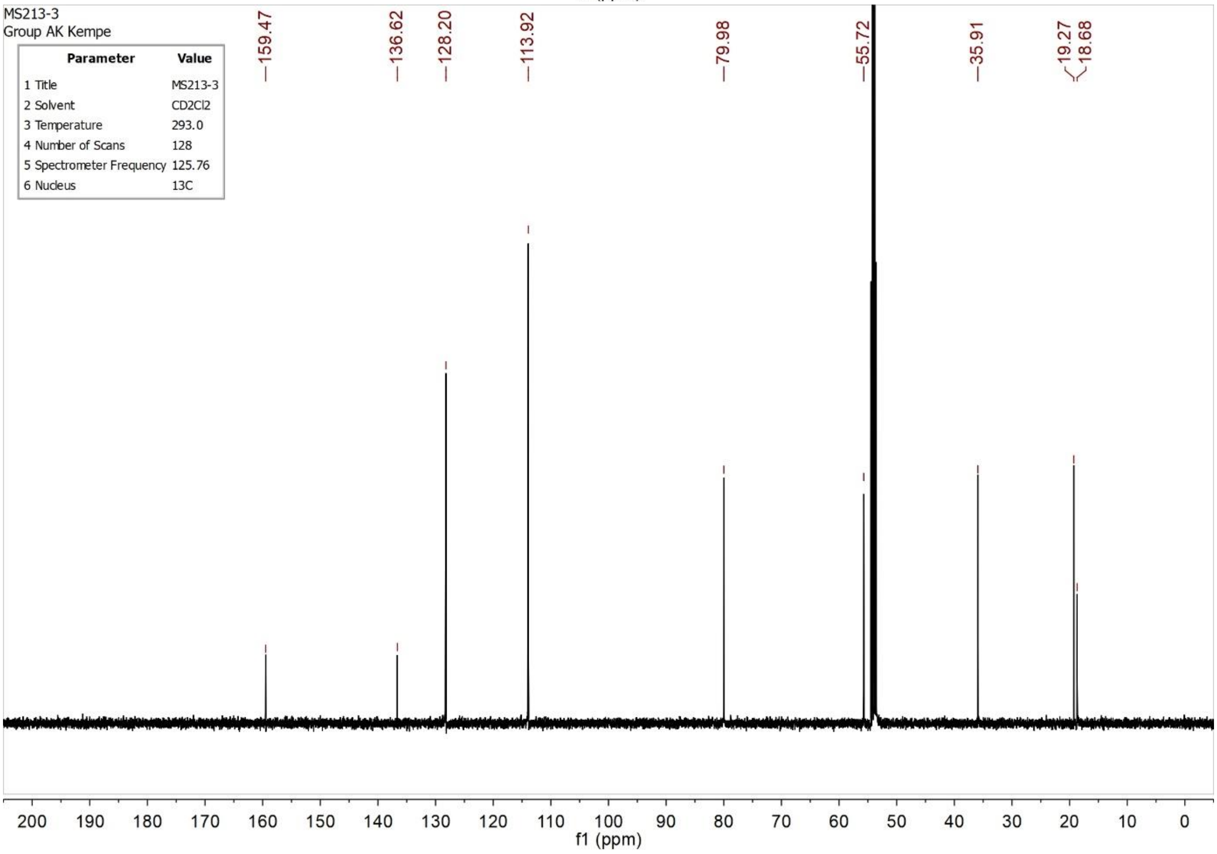

2-methyl-1-(*m*-tolyl)propan-1-ol (B5)

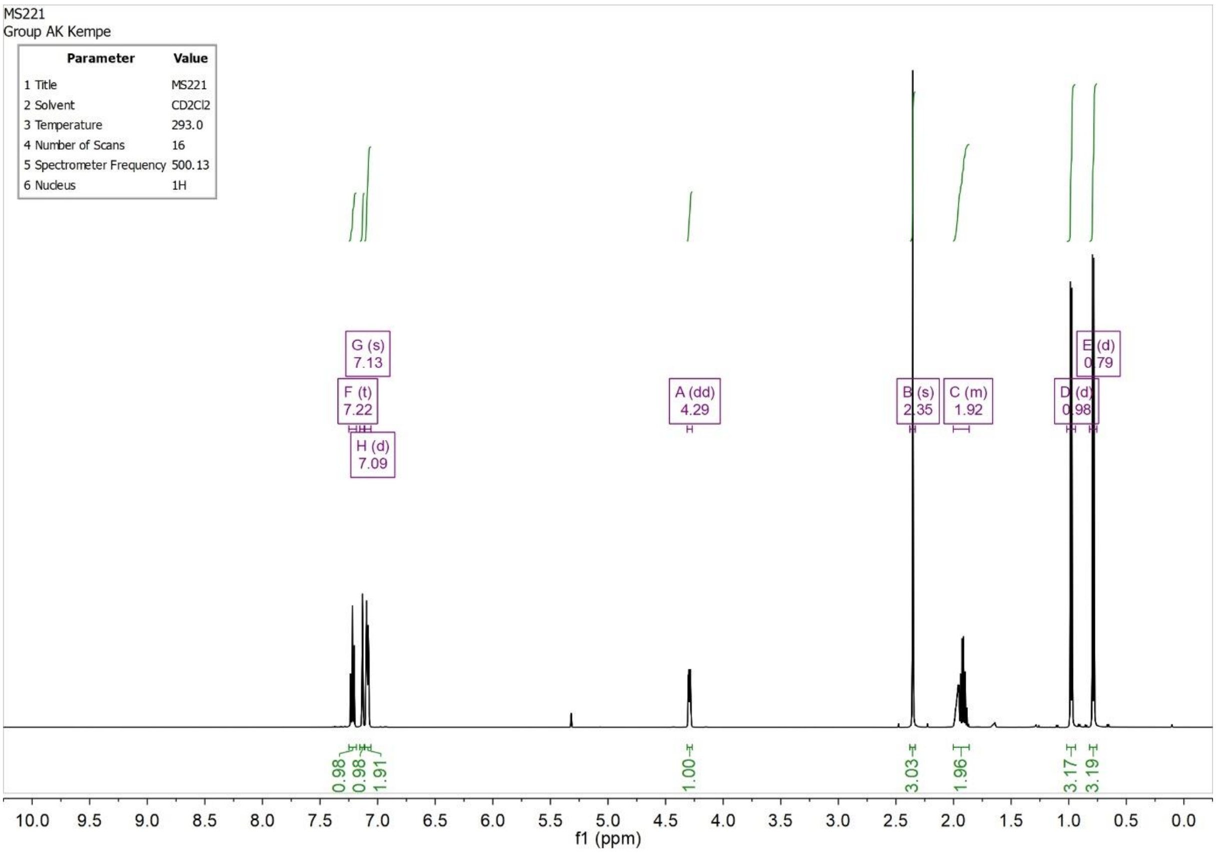

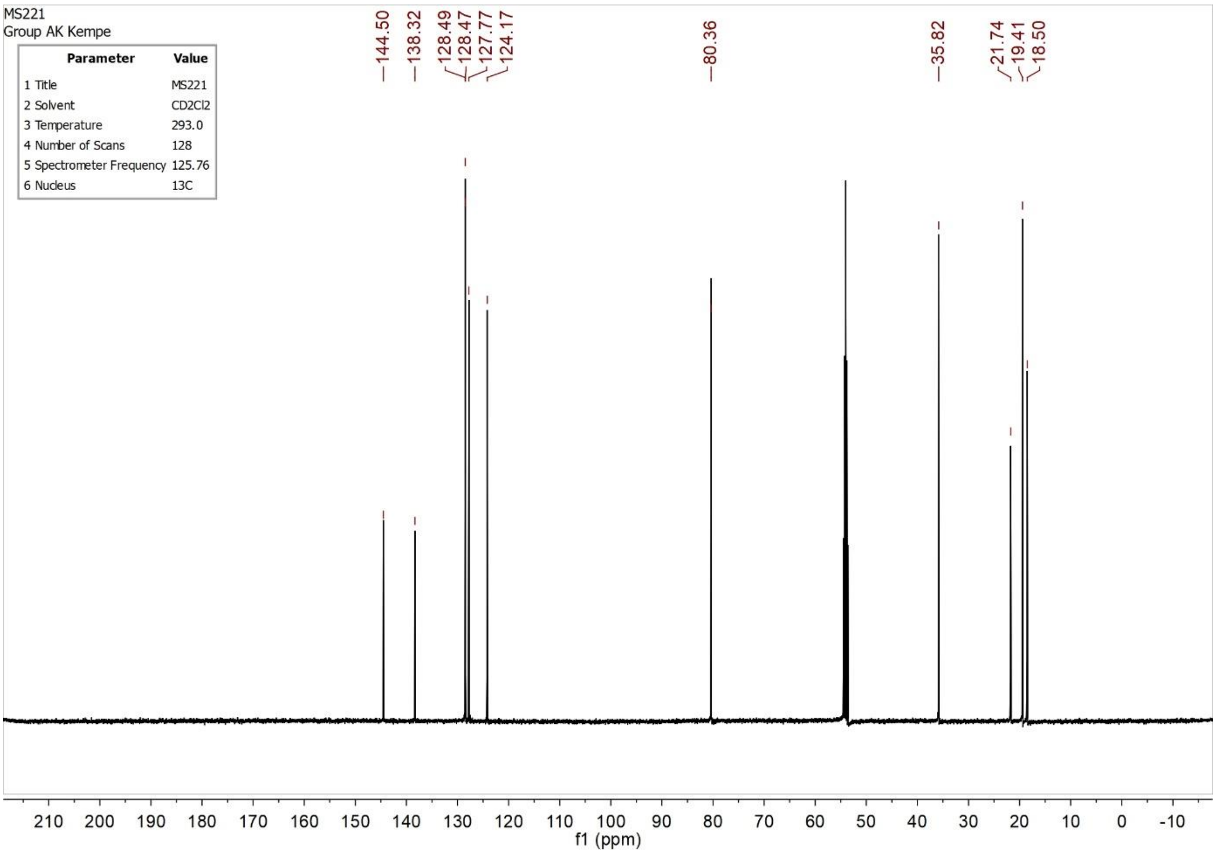

2-methyl-1-(*p*-tolyl)propan-1-ol (B6 / B21)

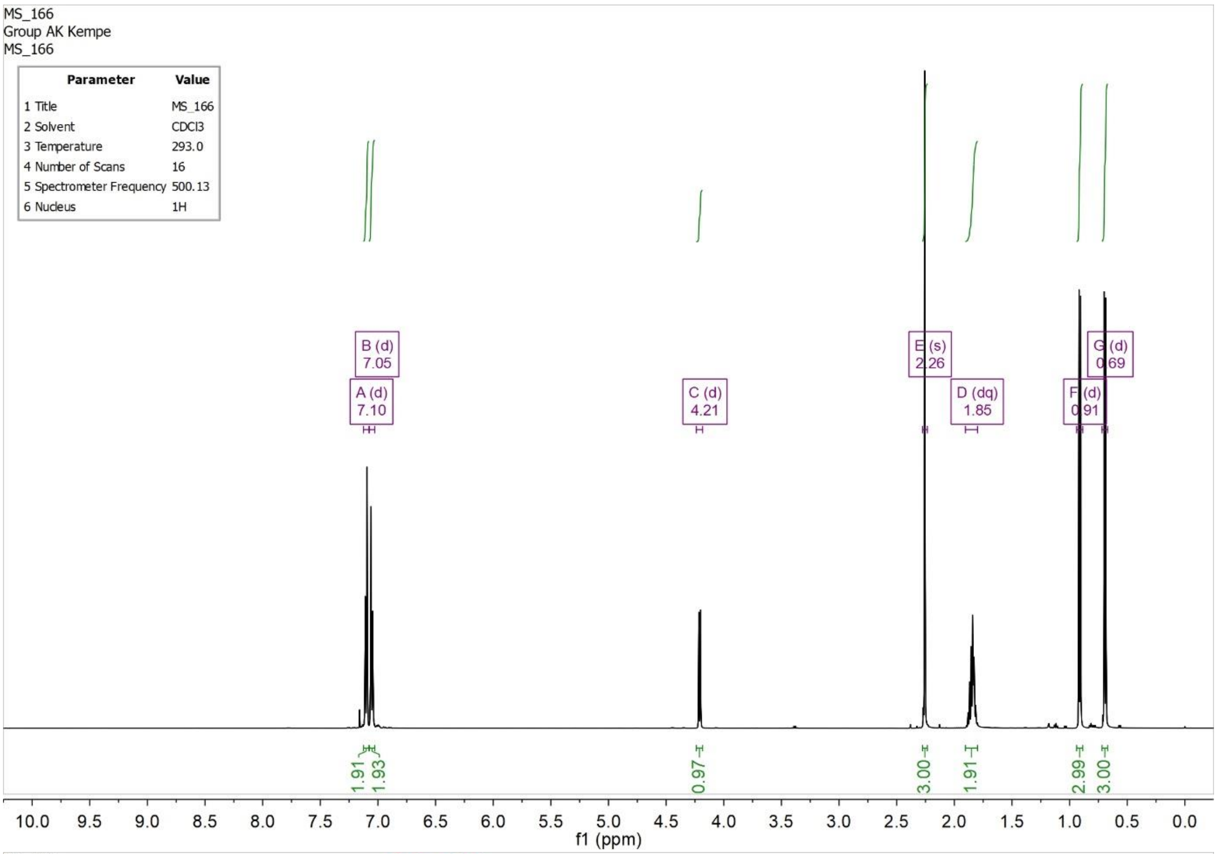

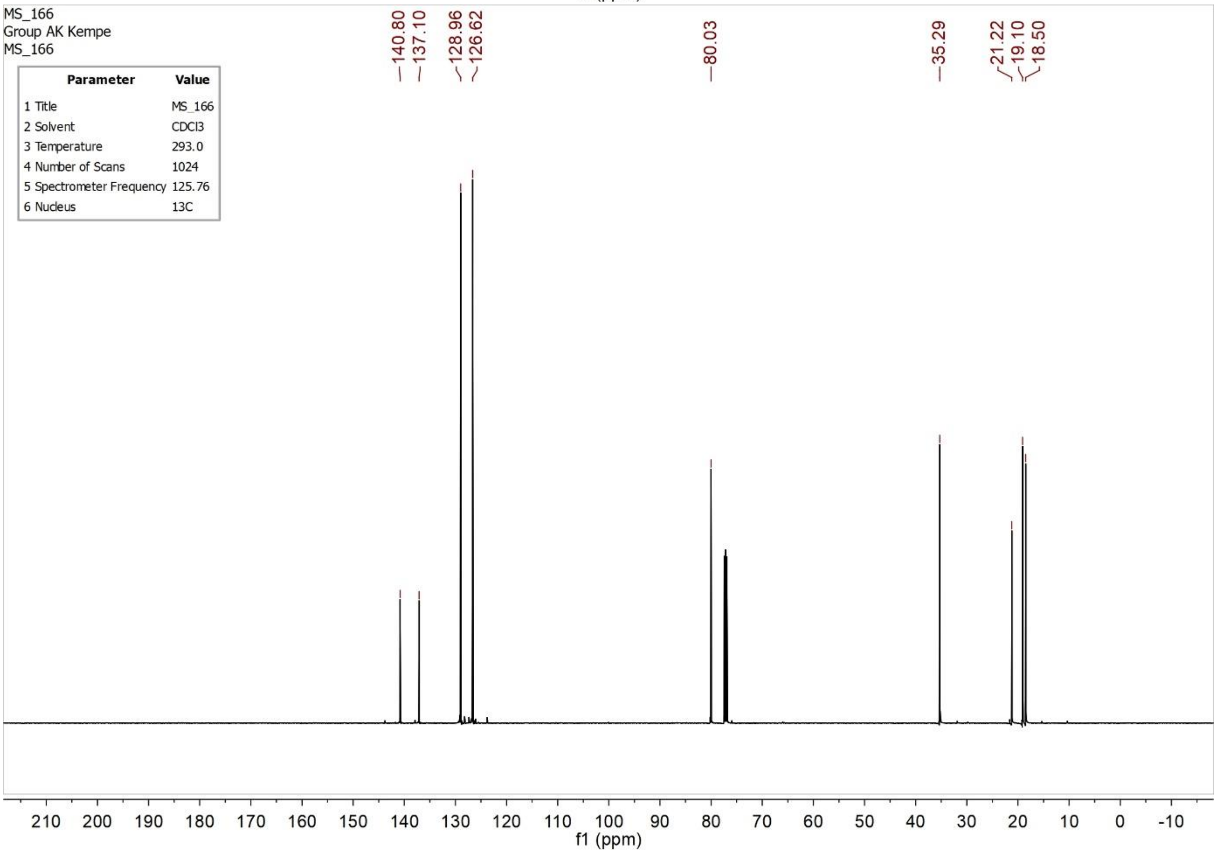

1-(3-fluorophenyl)-2-methylpropan-1-ol (B7)

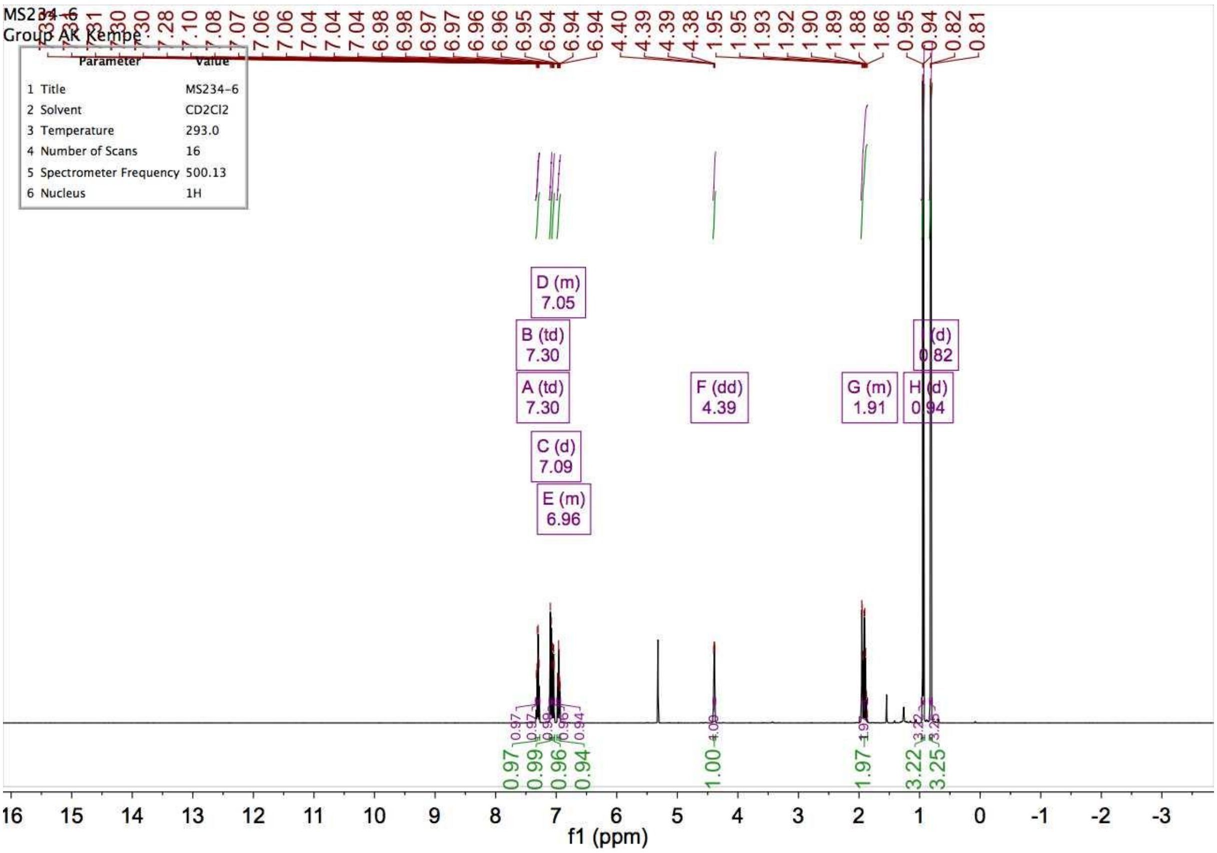

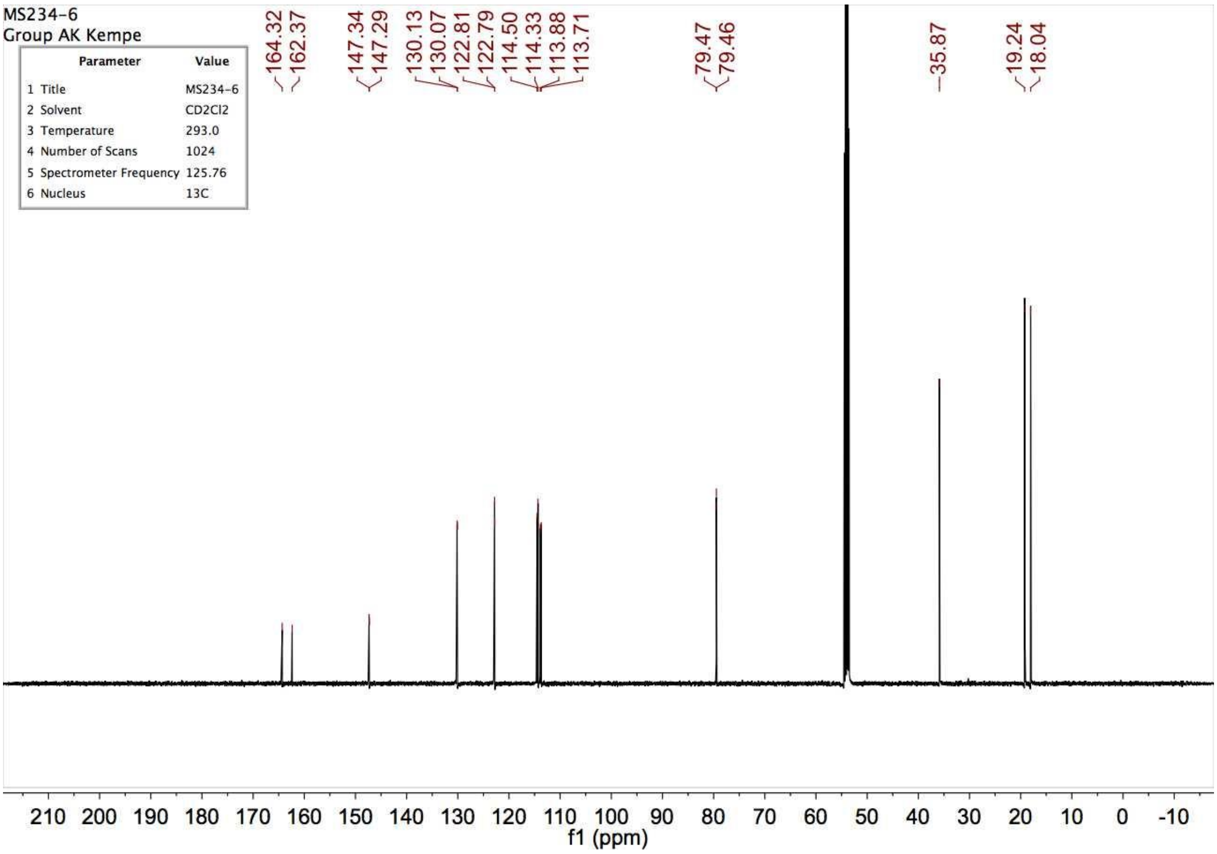

1-(3-chlorophenyl)-2-methylpropan-1-ol (B8)

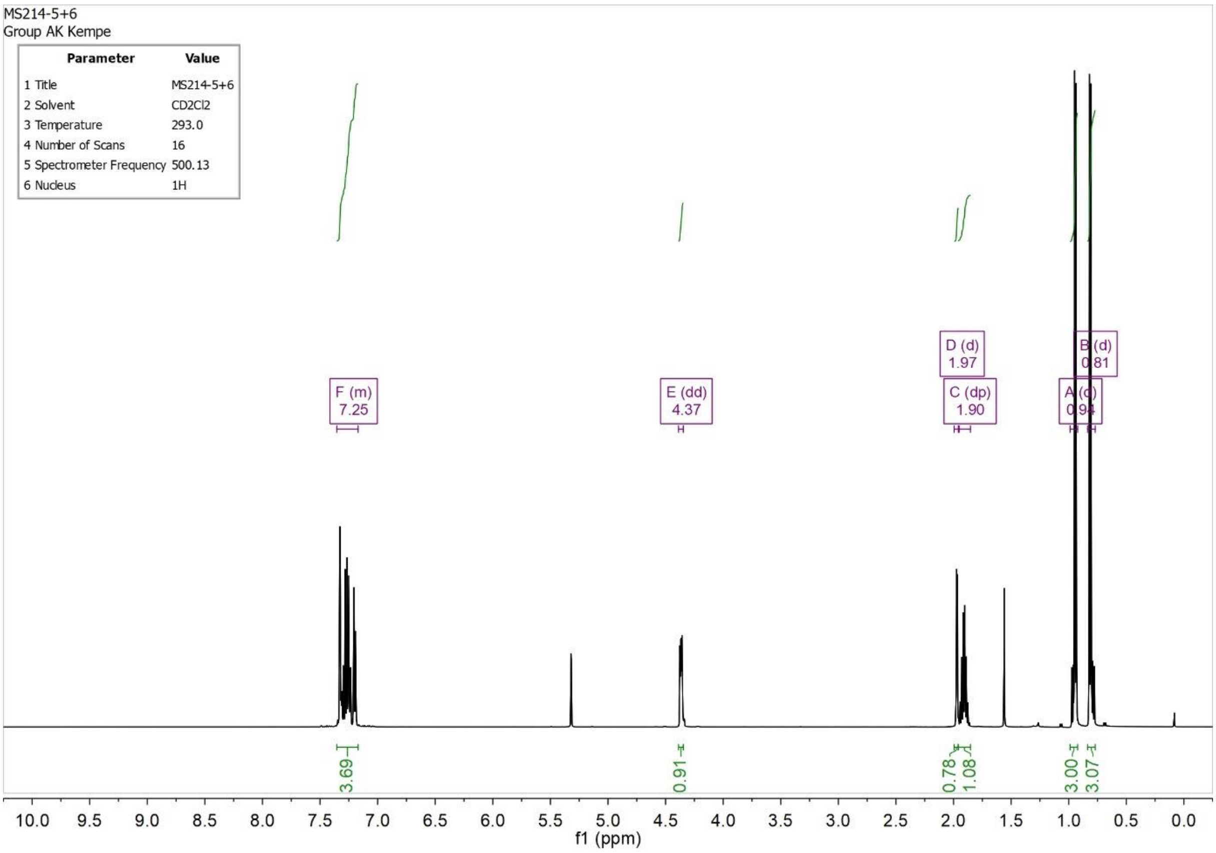

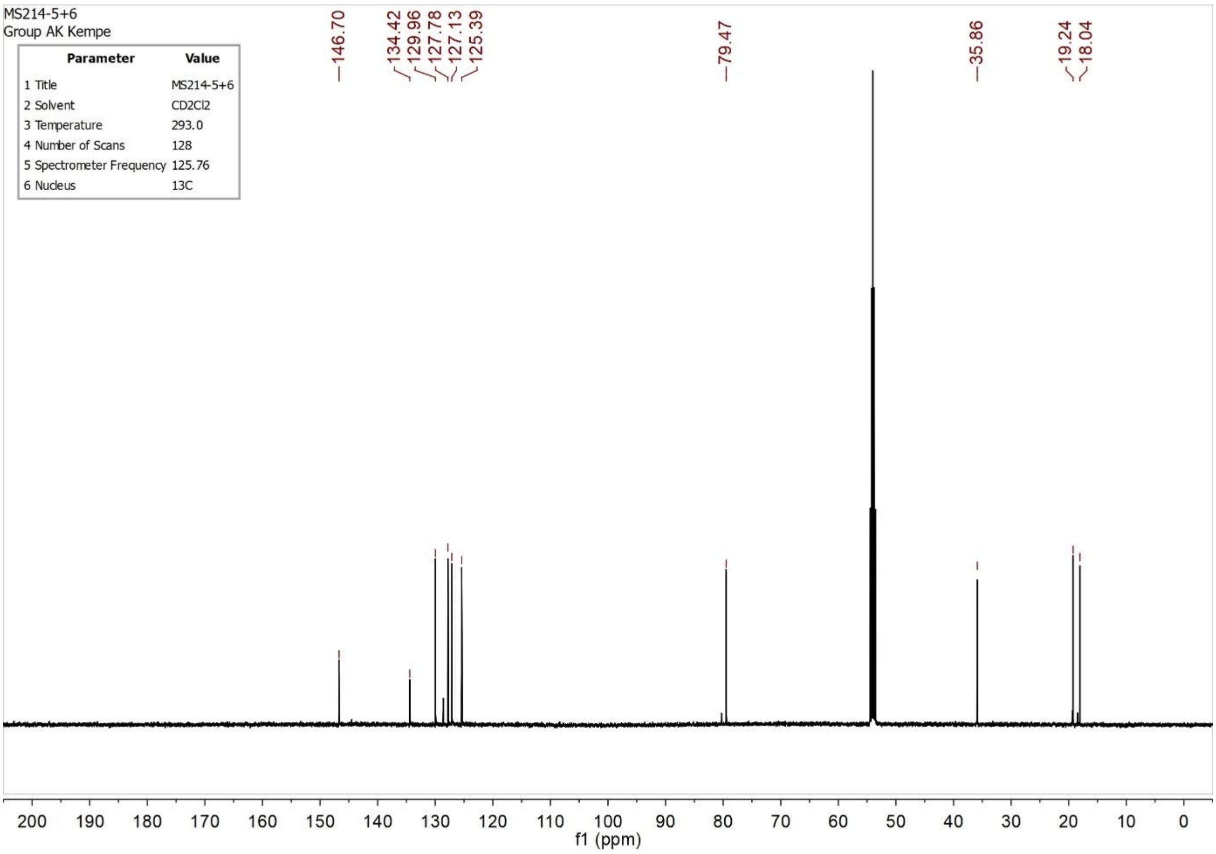

1-(3-bromophenyl)-2-methylpropan-1-ol (B9)

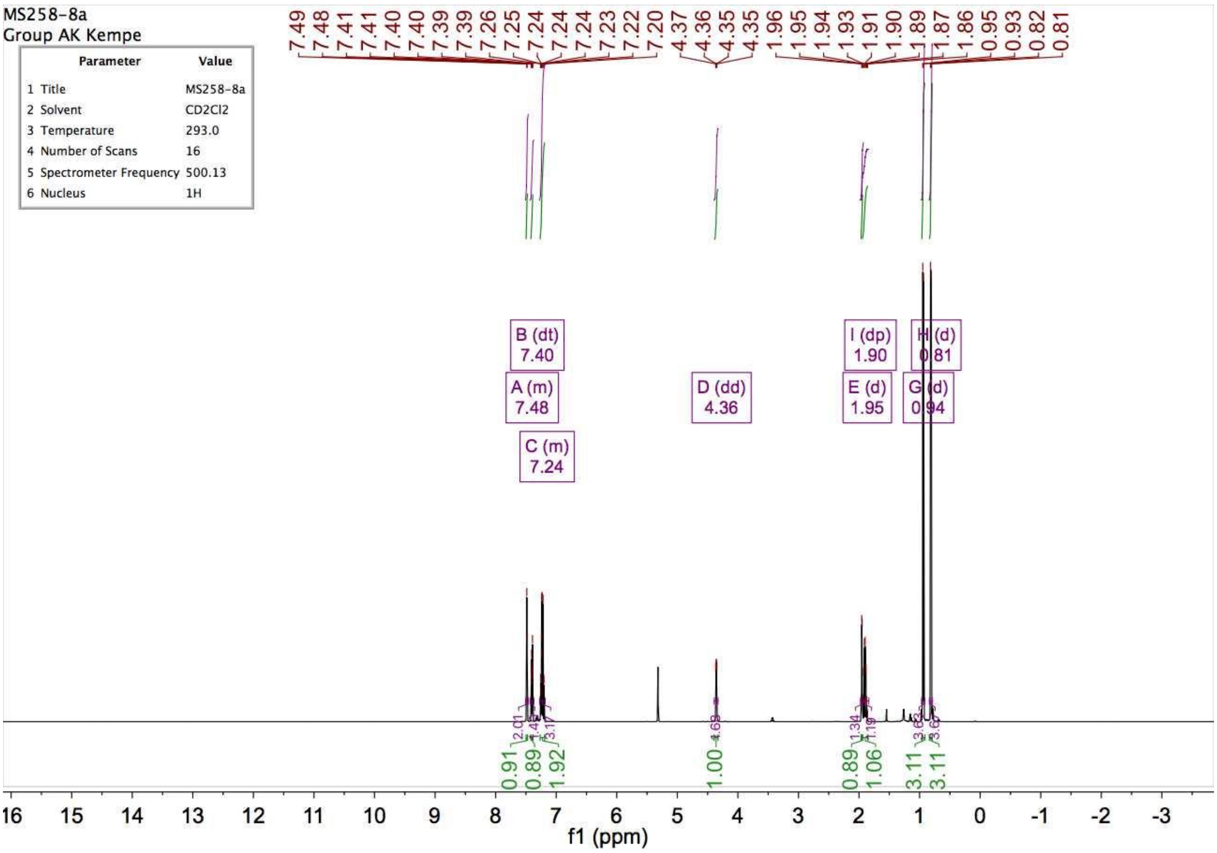

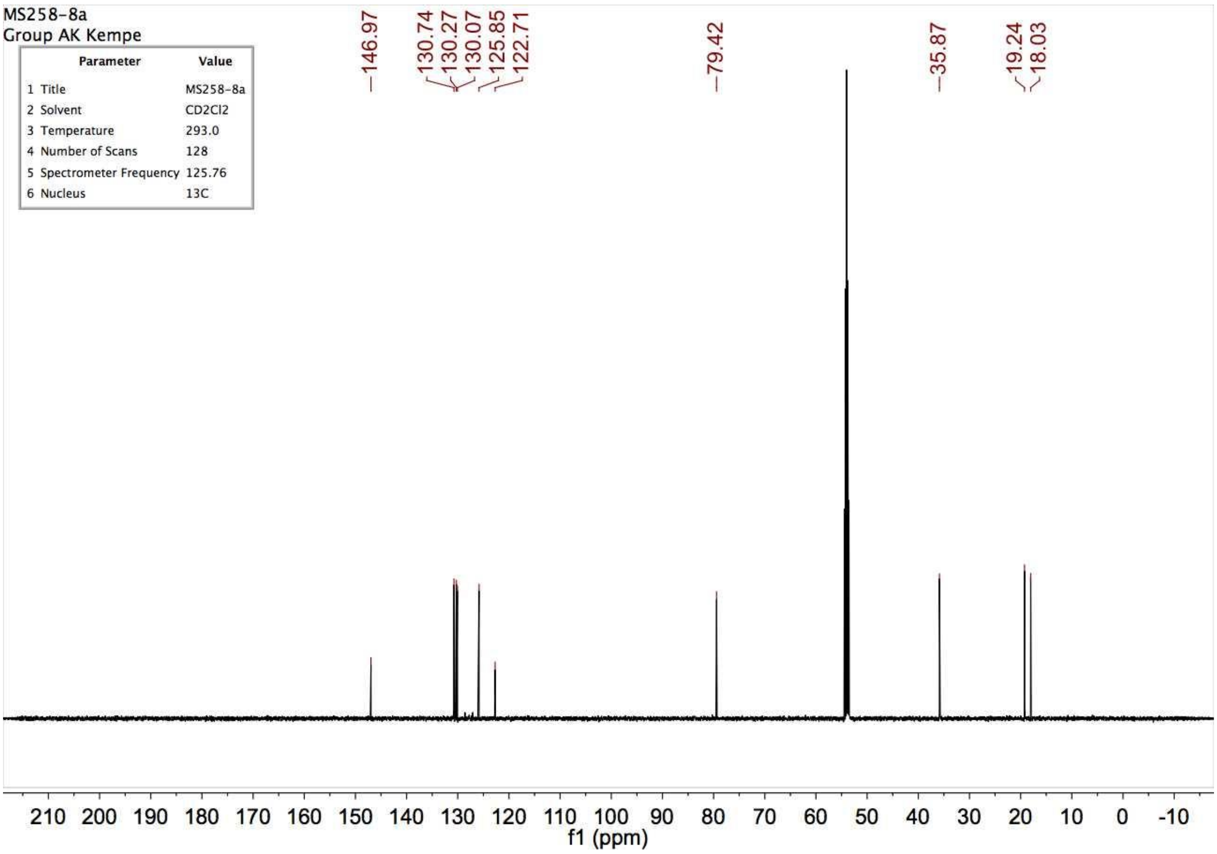

1-(3-iodophenyl)-2-methylpropan-1-ol (B10)

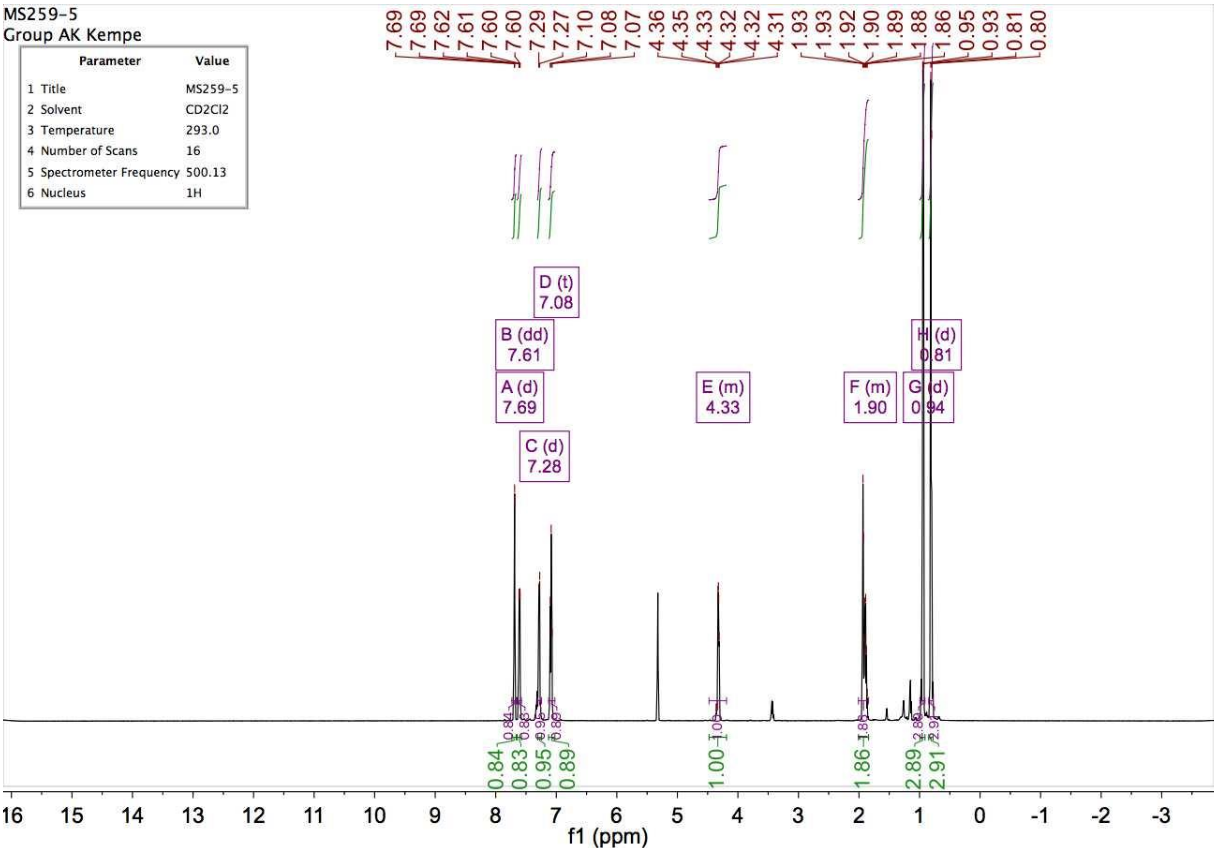

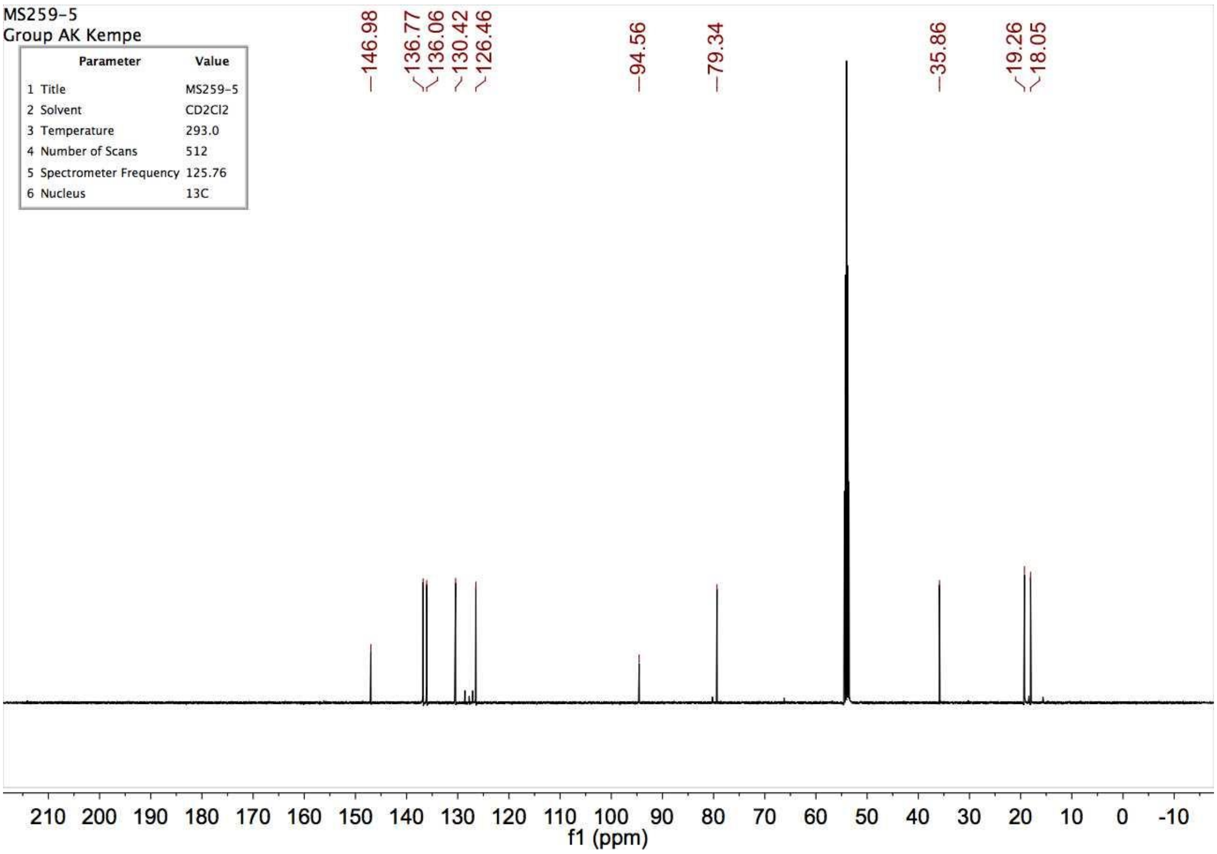

2-methyl-1-(4-(trifluoromethyl)phenyl)propan-1-ol (B11)

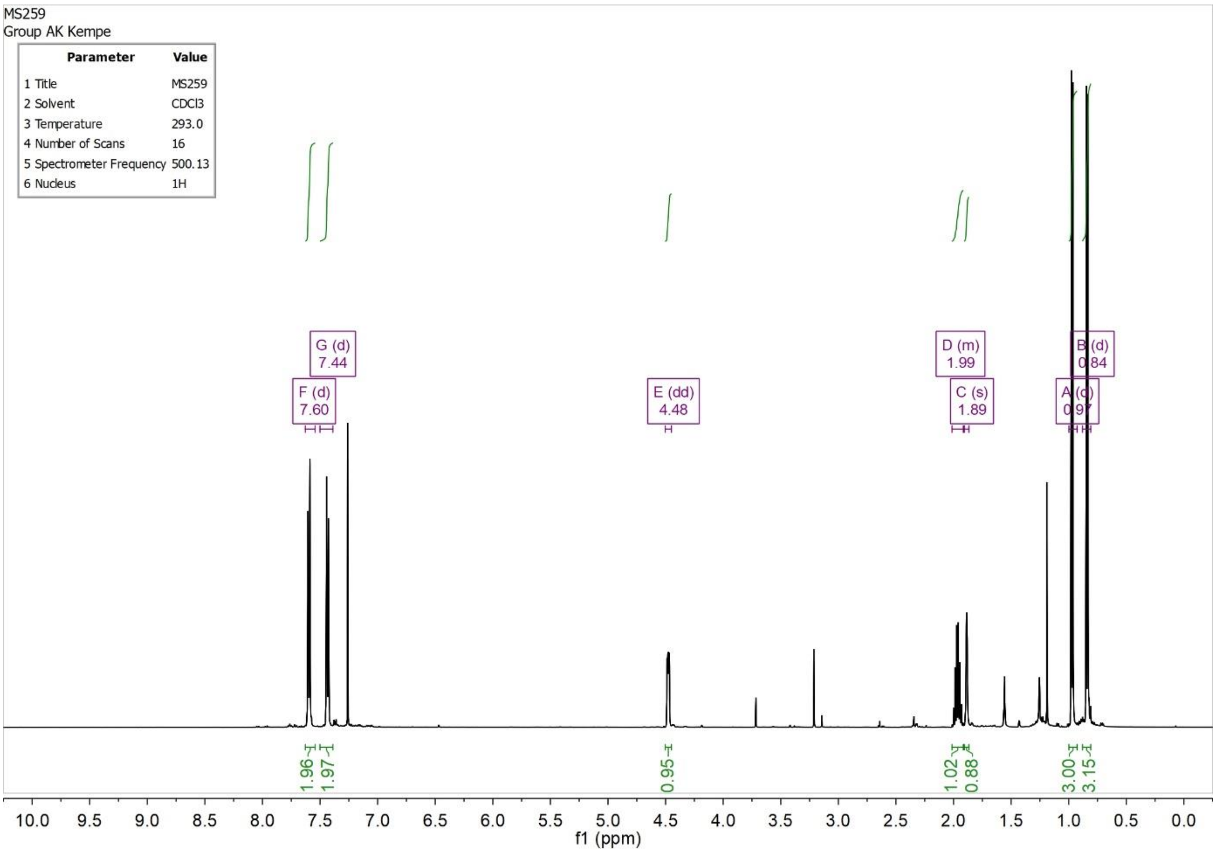

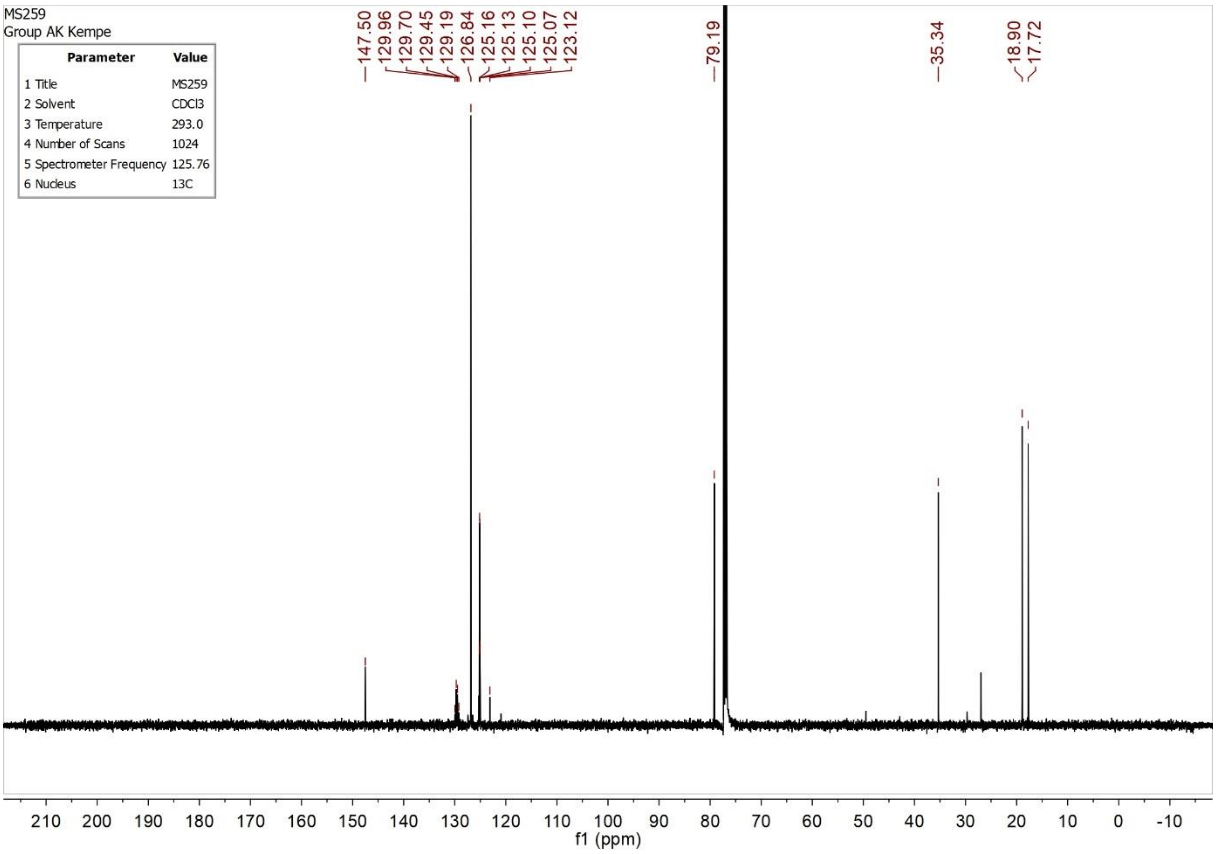

1-(4-*(tert*-butyl)phenyl)-2-methylpropan-1-ol (B12)

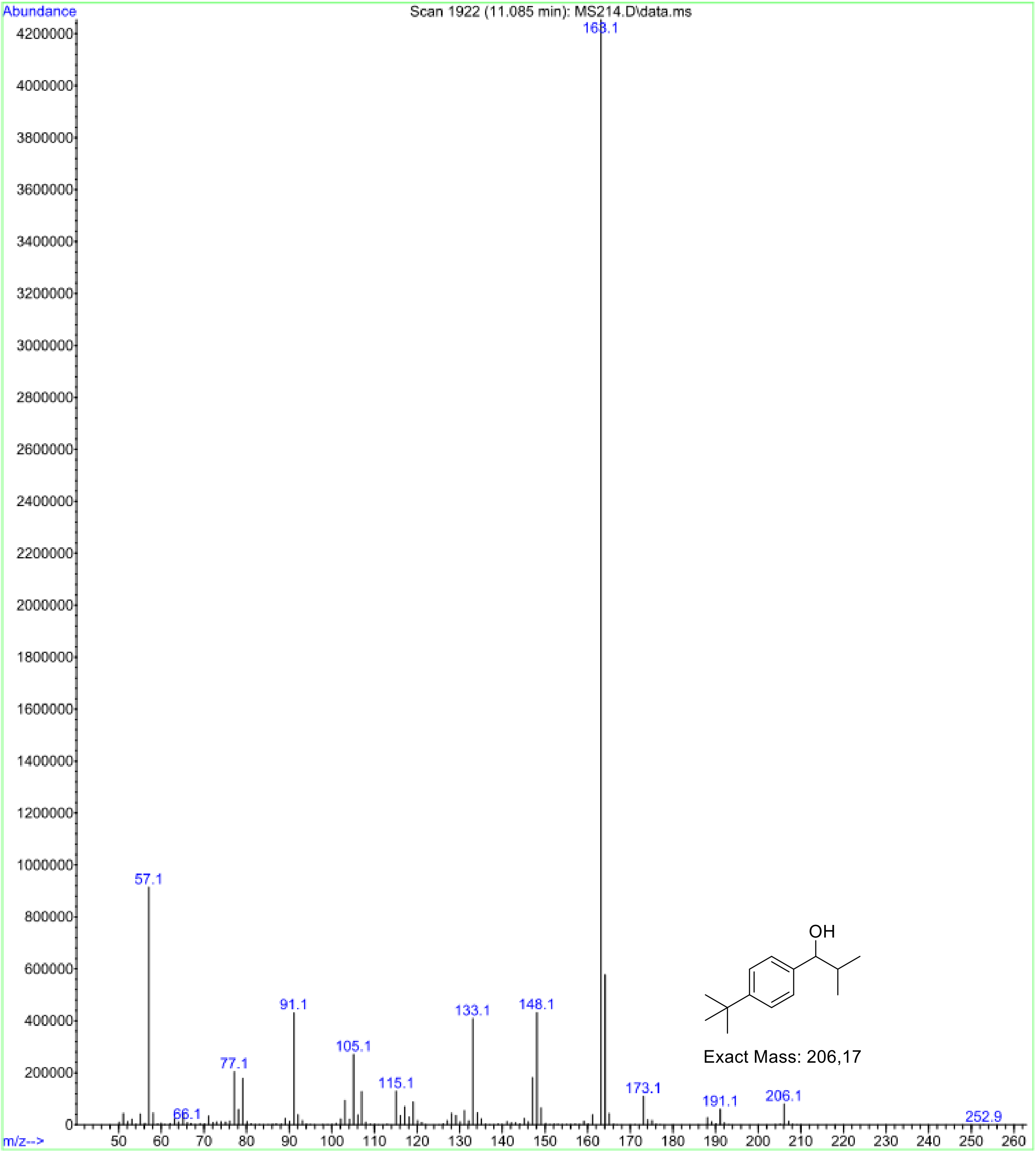

1-(3,4-dimethoxyphenyl)-2-methylpropan-1-ol (B13)

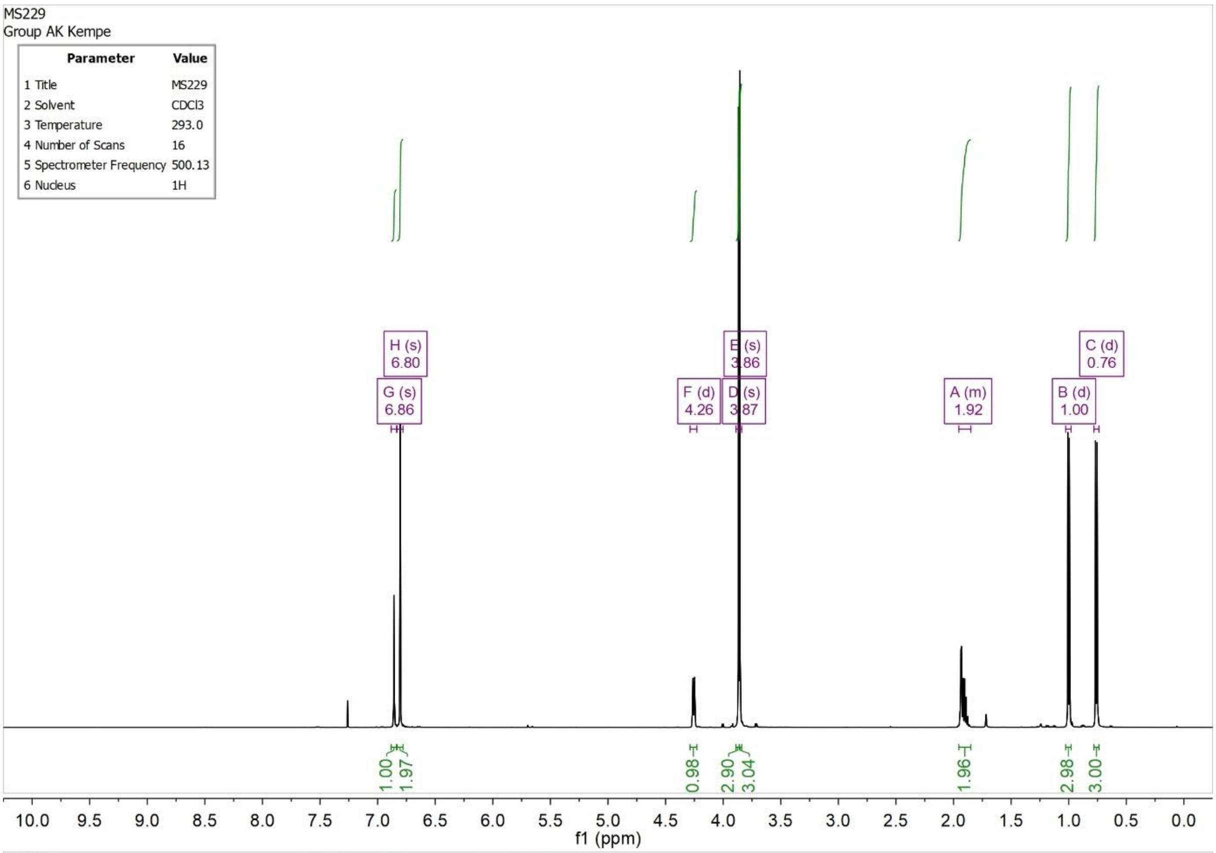

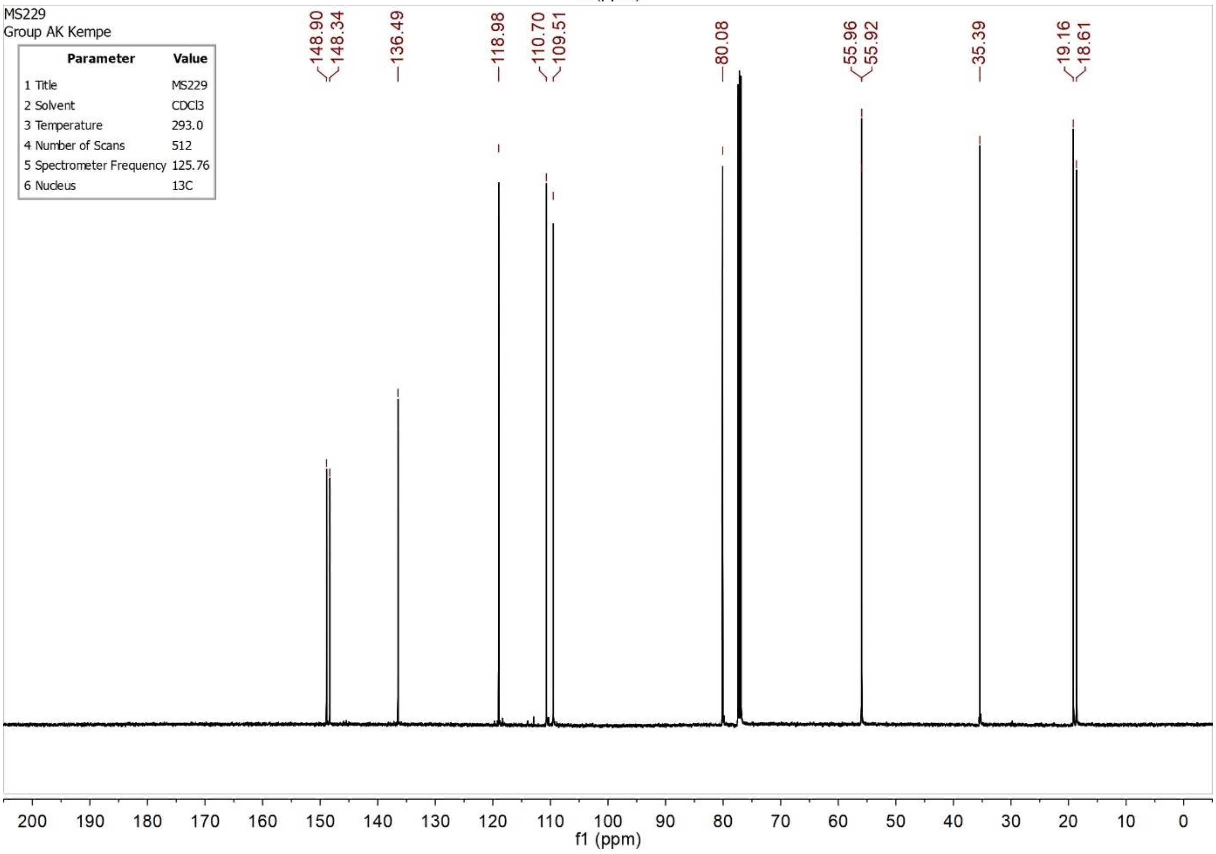

2-methyl-1-(4-(pyrrolidin-1-yl)phenyl)propan-1-ol (B14 / B23)

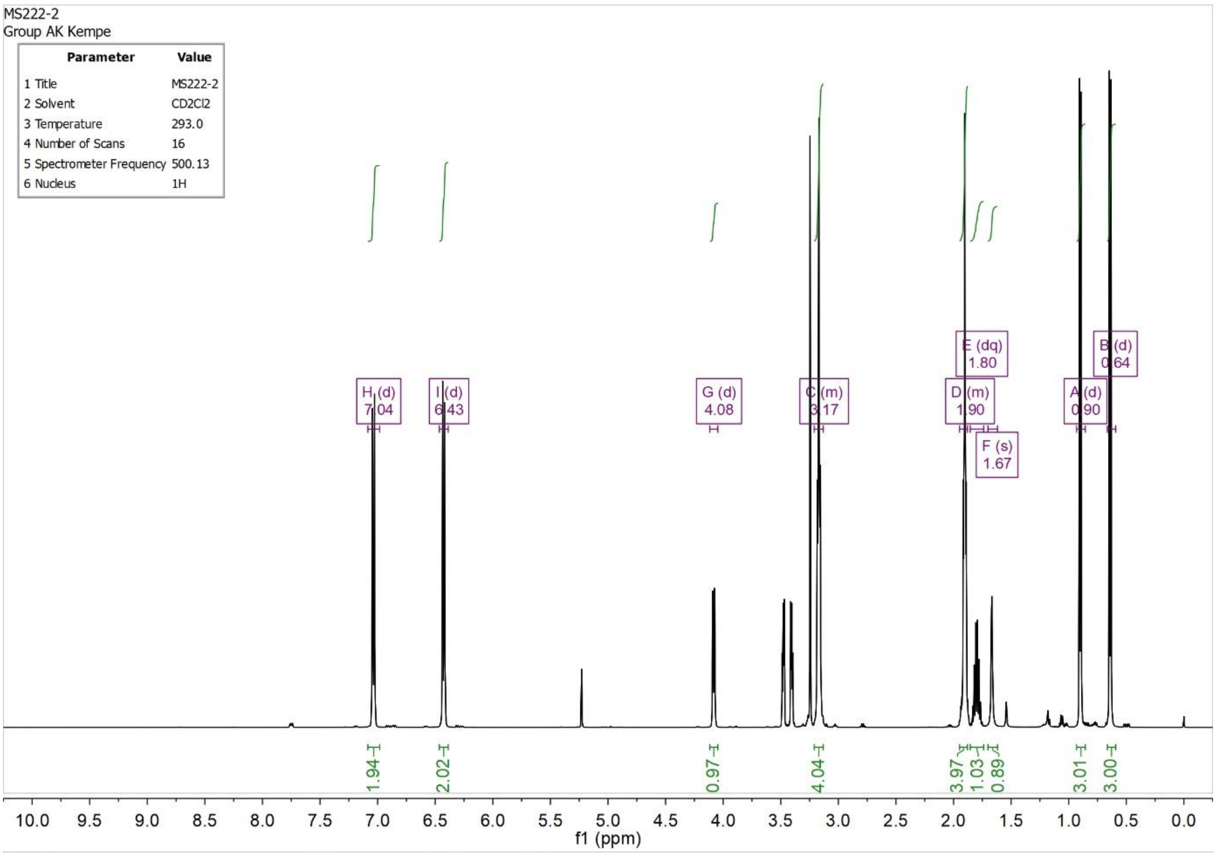

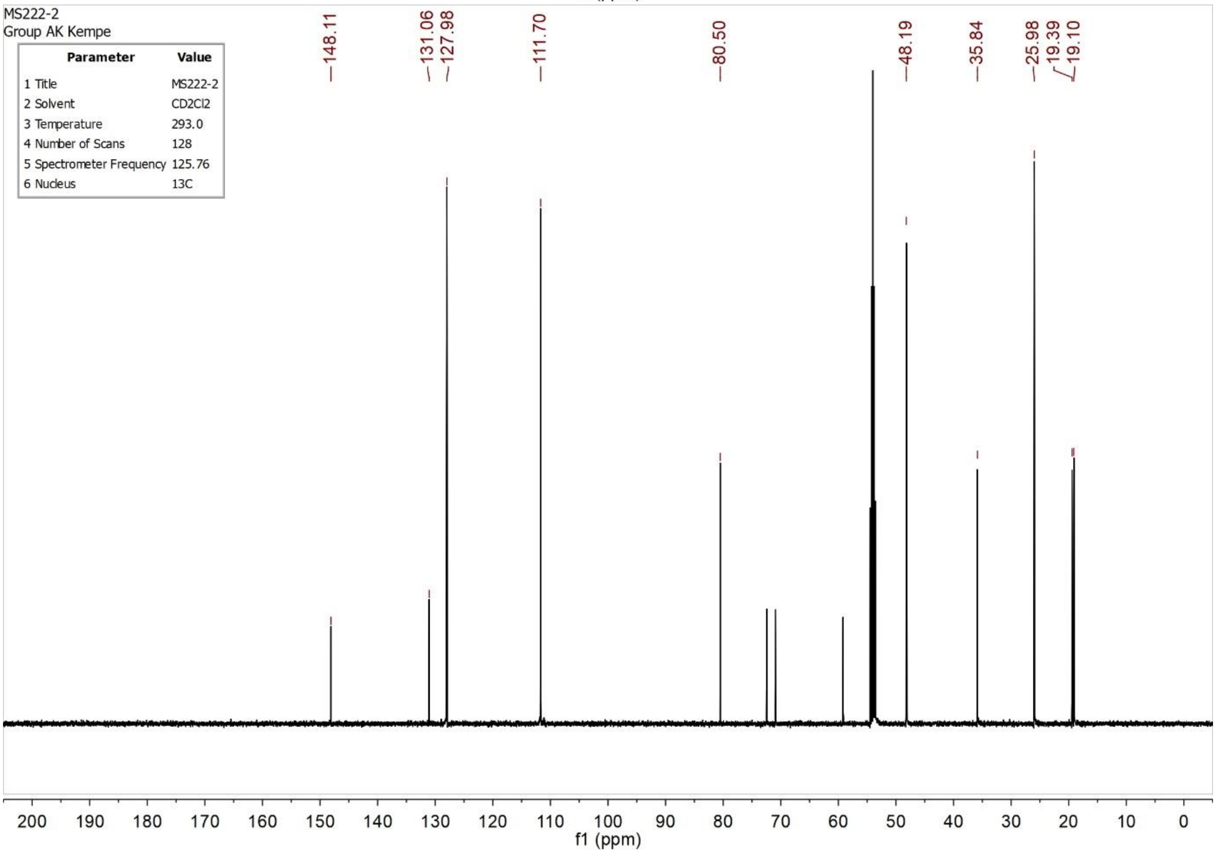

2-methyl-1-(naphthalen-2-yl)propan-1-ol (B15)

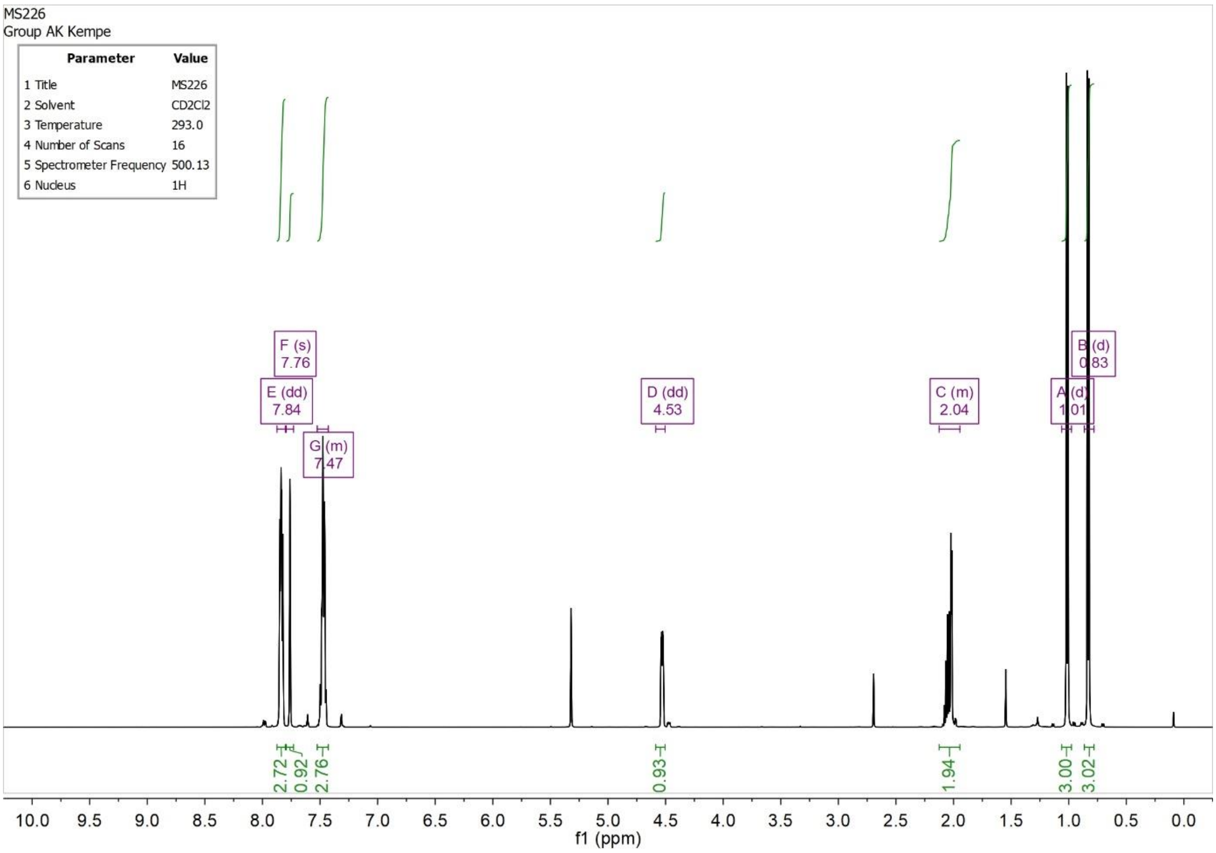

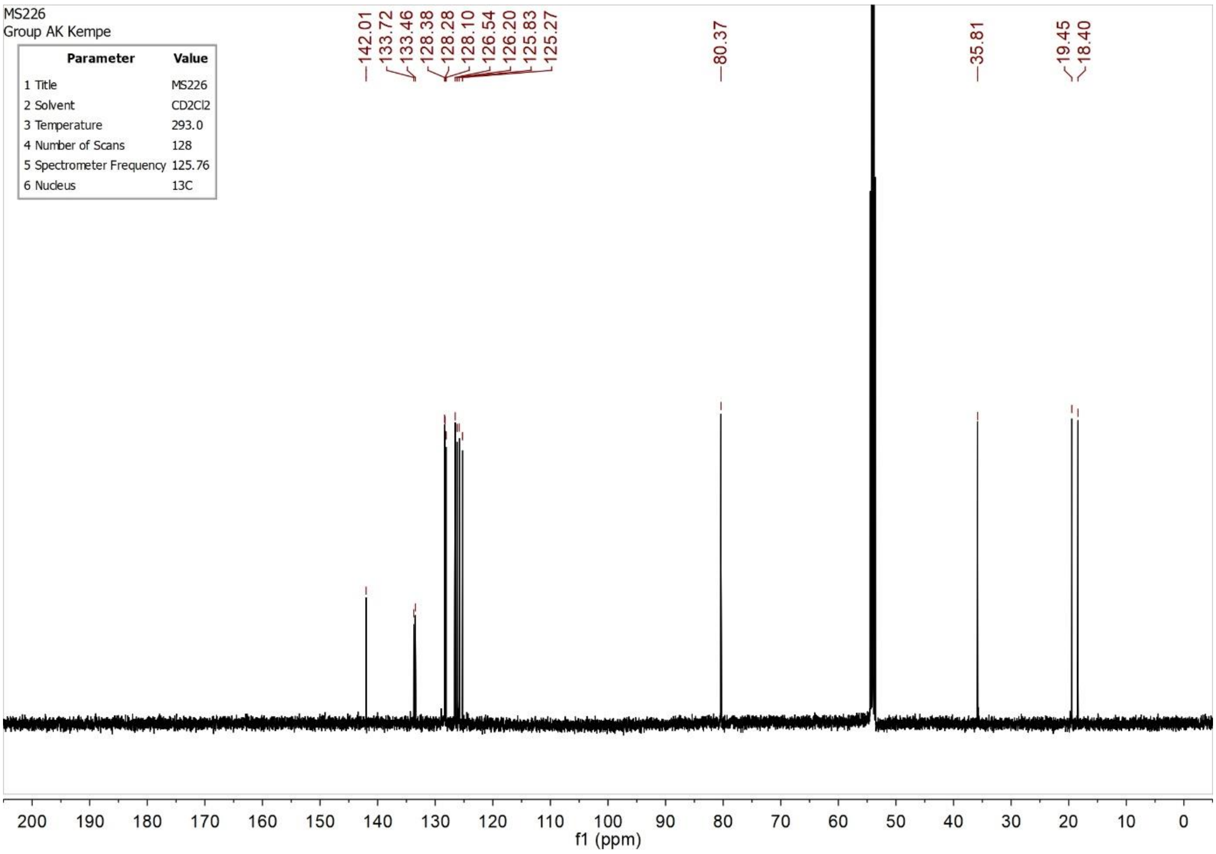

1-(6-methoxynaphthalen-2-yl)-2-methylpropan-1-ol (B16)

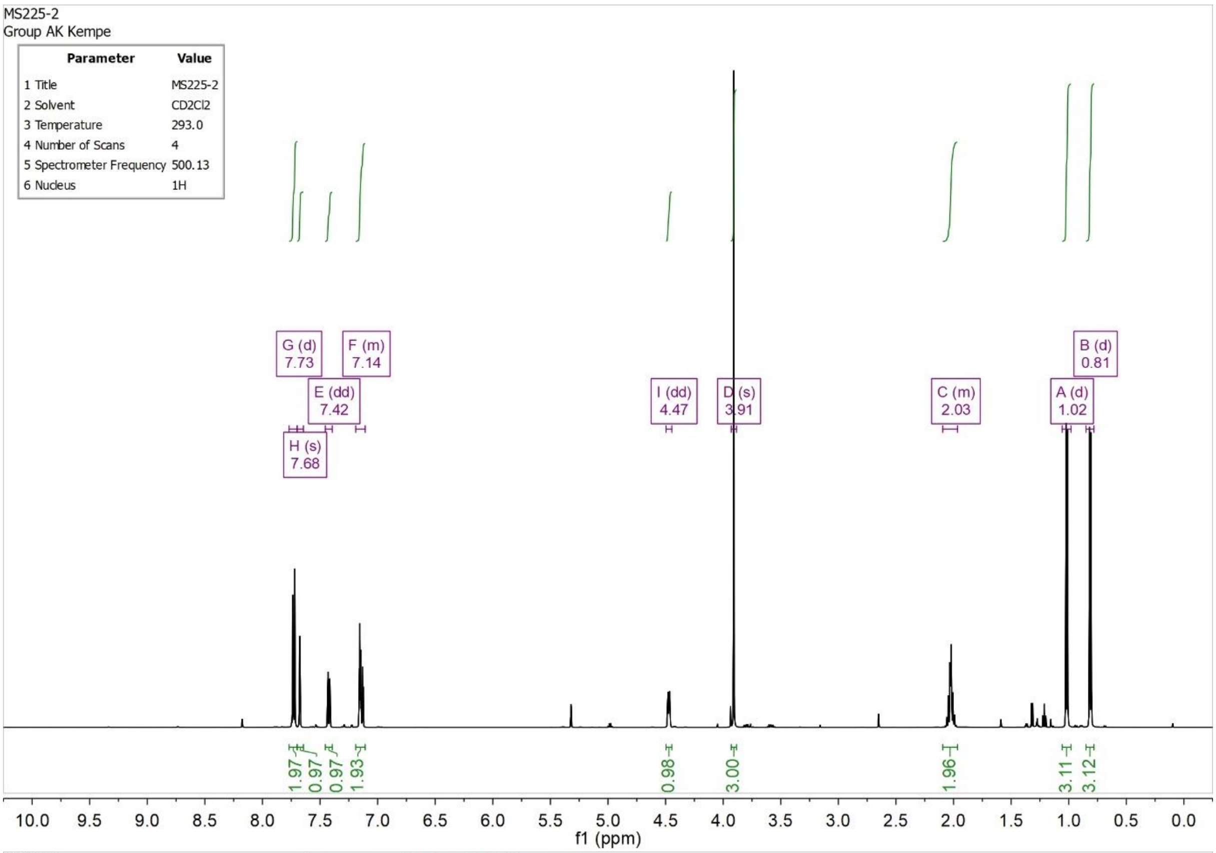

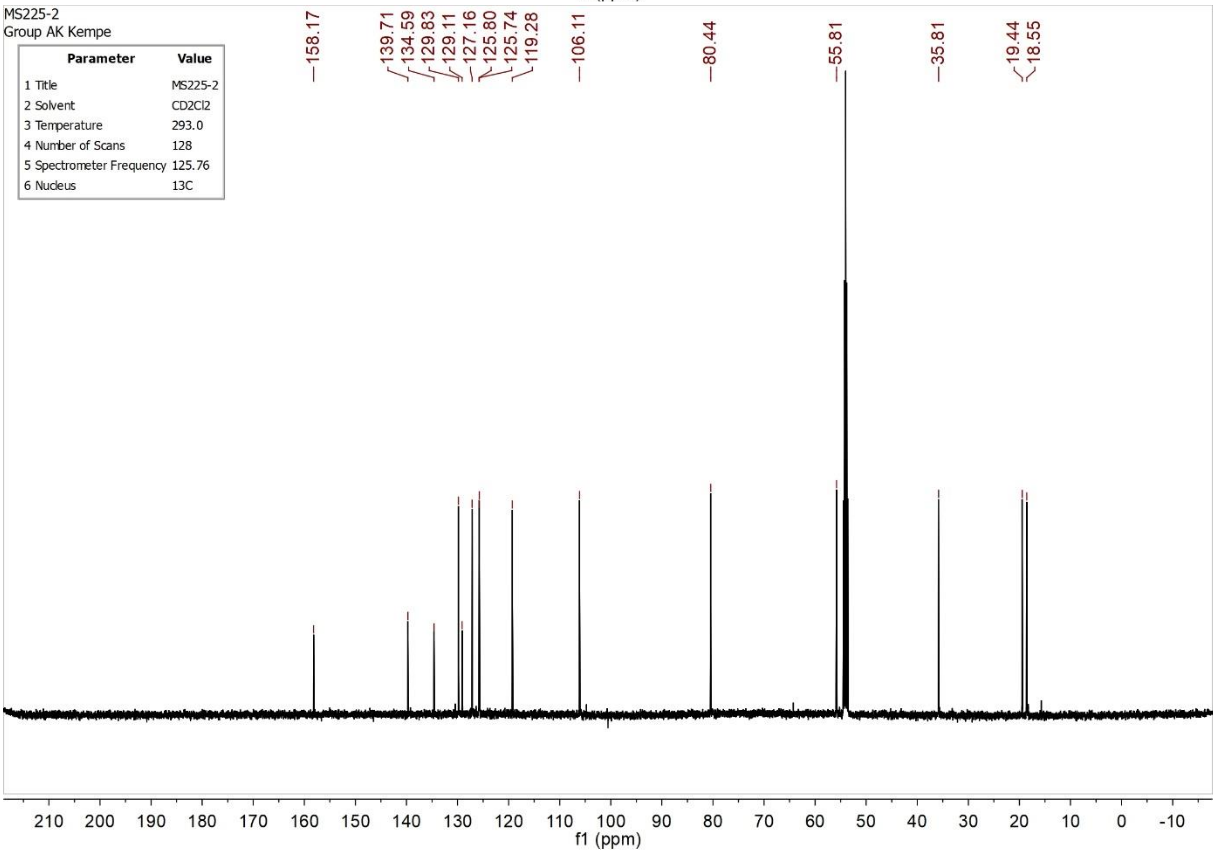

2-methyl-1-(pyridin-3-yl)propan-1-ol (B17)

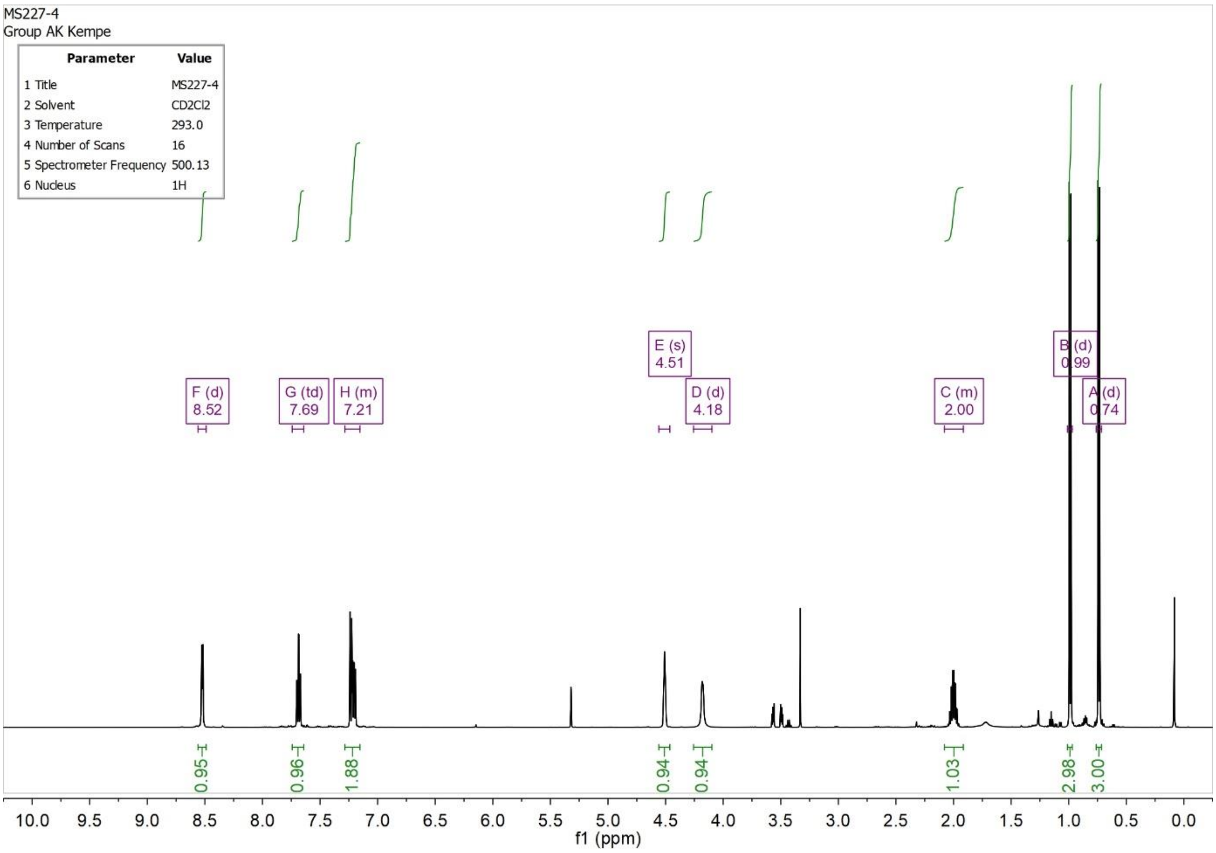

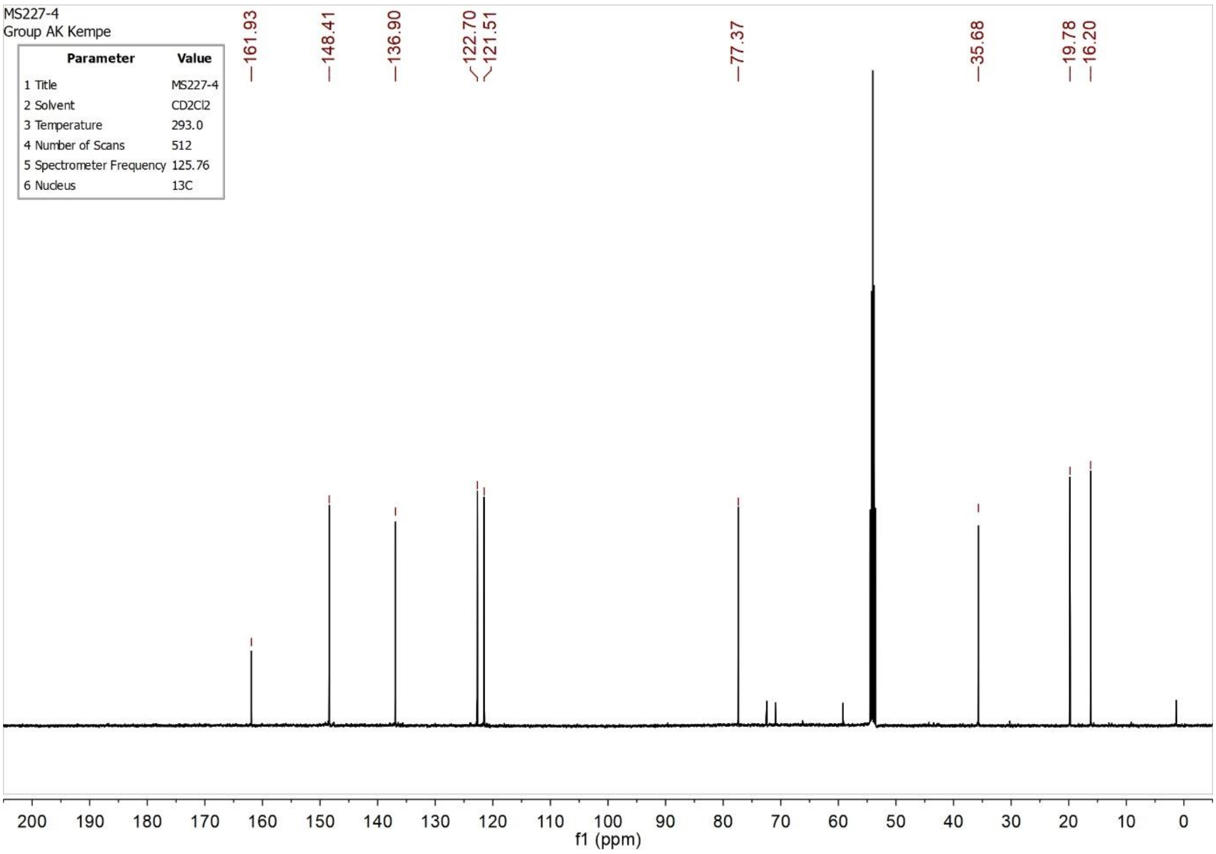

2-methyl-1-(ferrocenyl)propanol (B18)

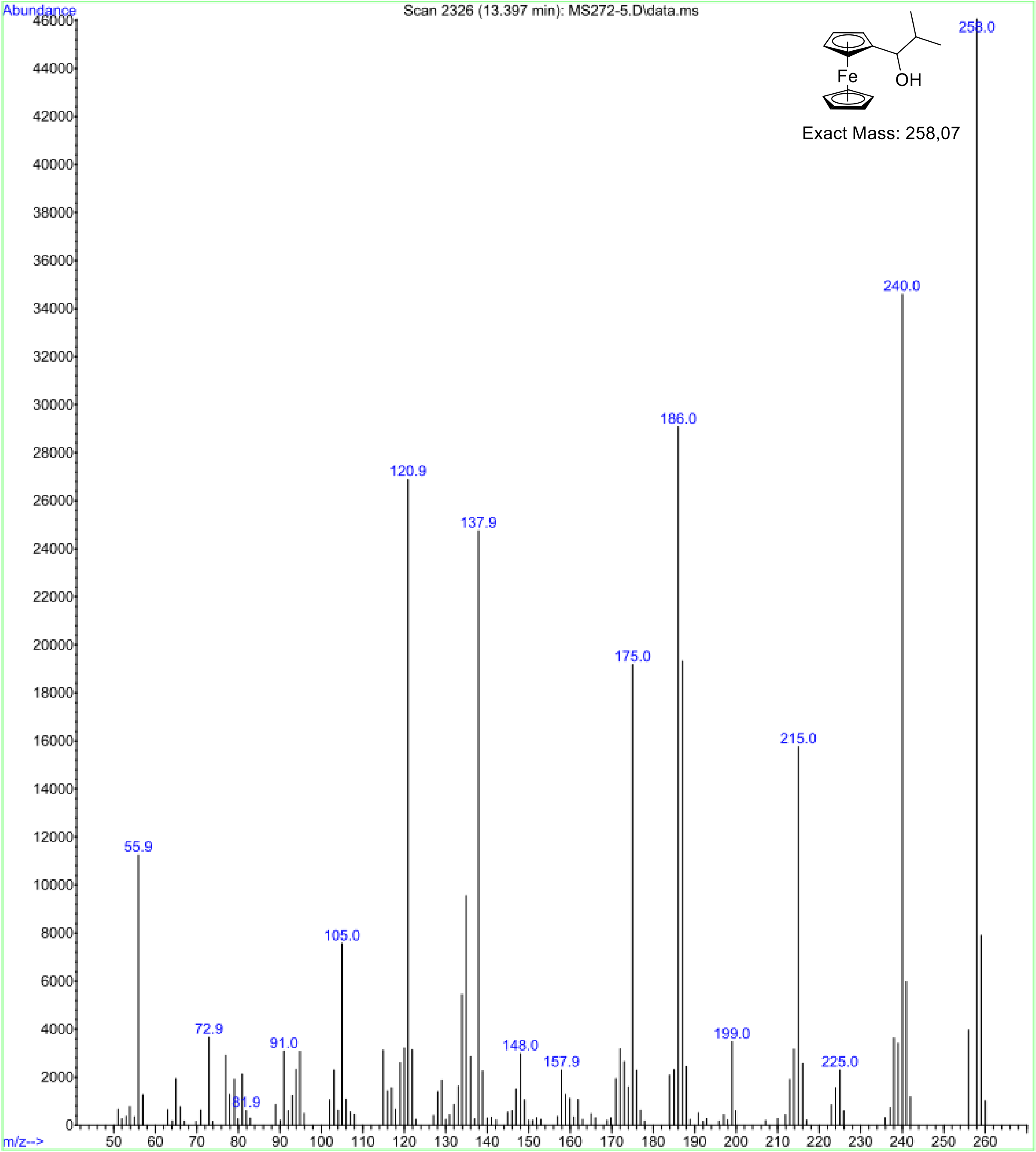

1-(2,3-dihydrobenzo[b][1,4]dioxin-6-yl)-2-methylpropan-1-ol (B22)

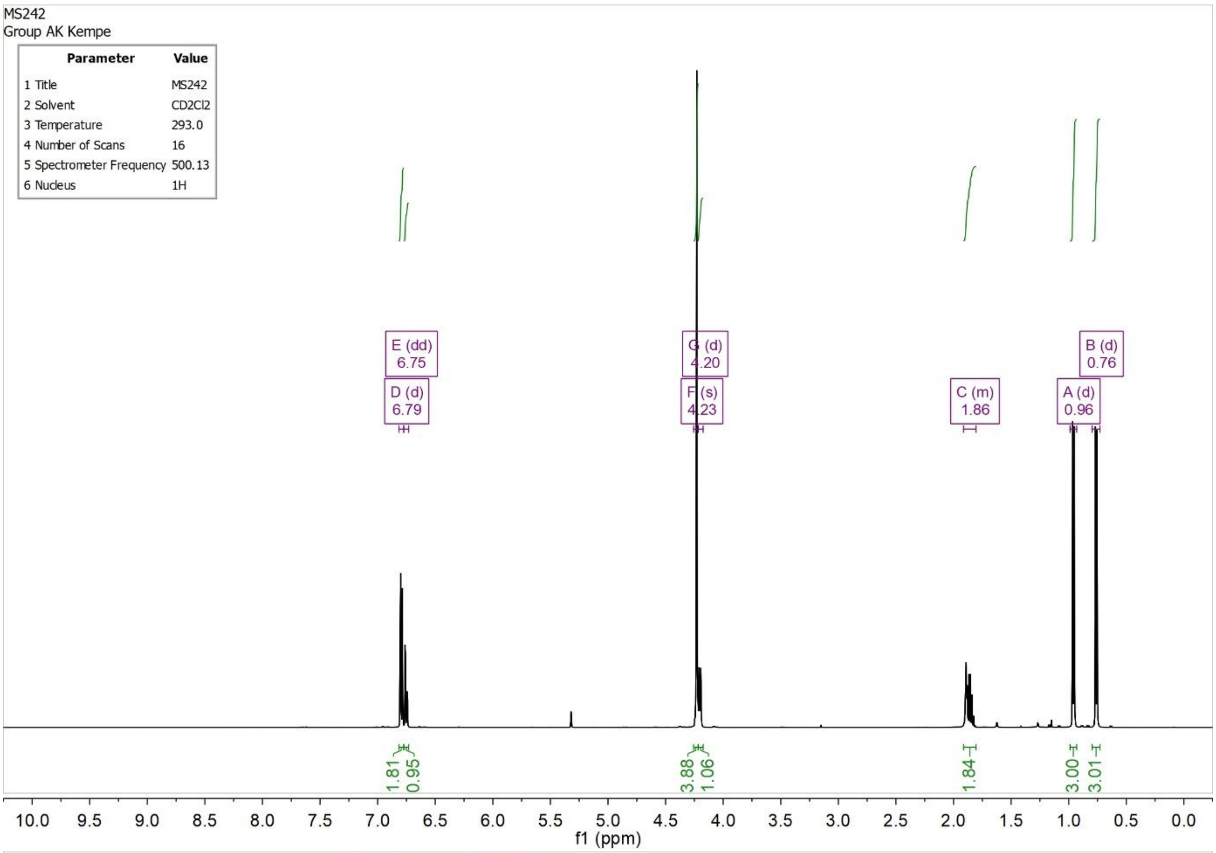

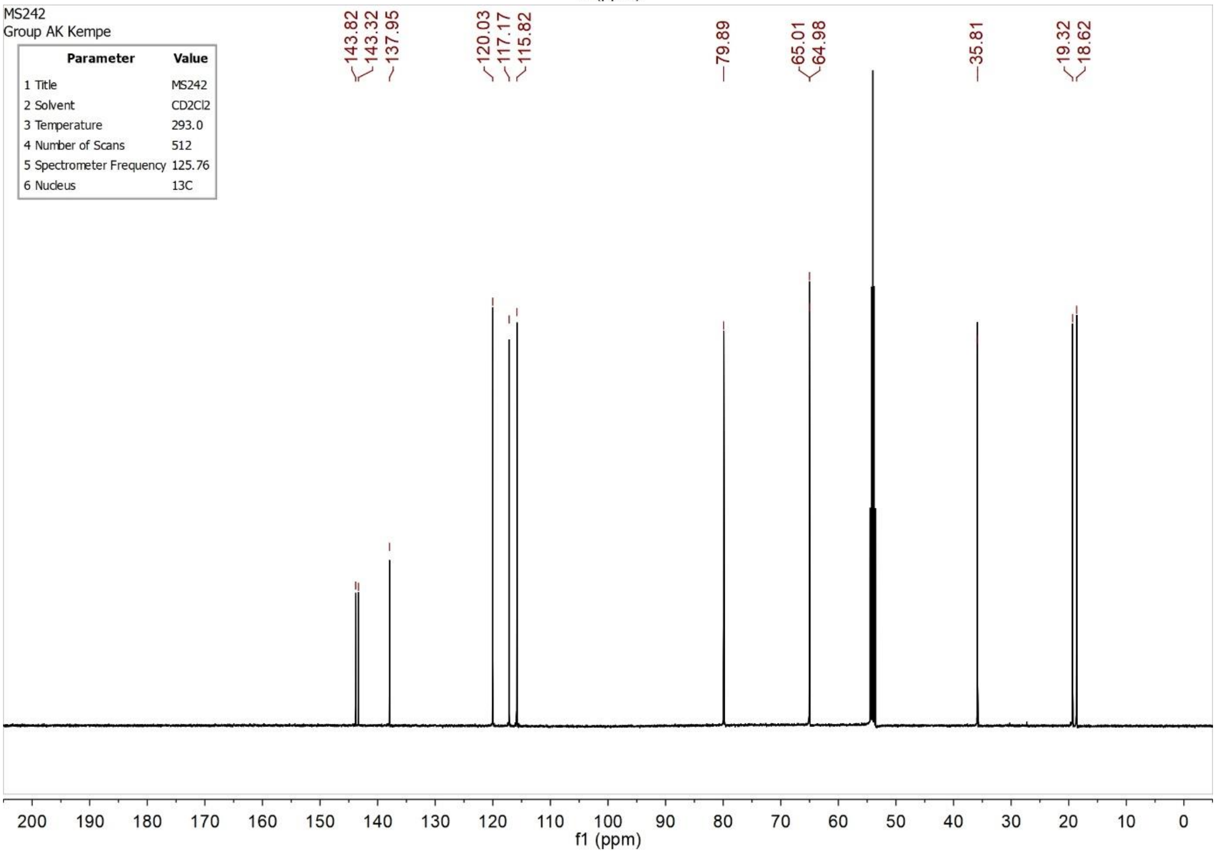

2-methyl-1,3-diphenylpropan-1-ol (B24)

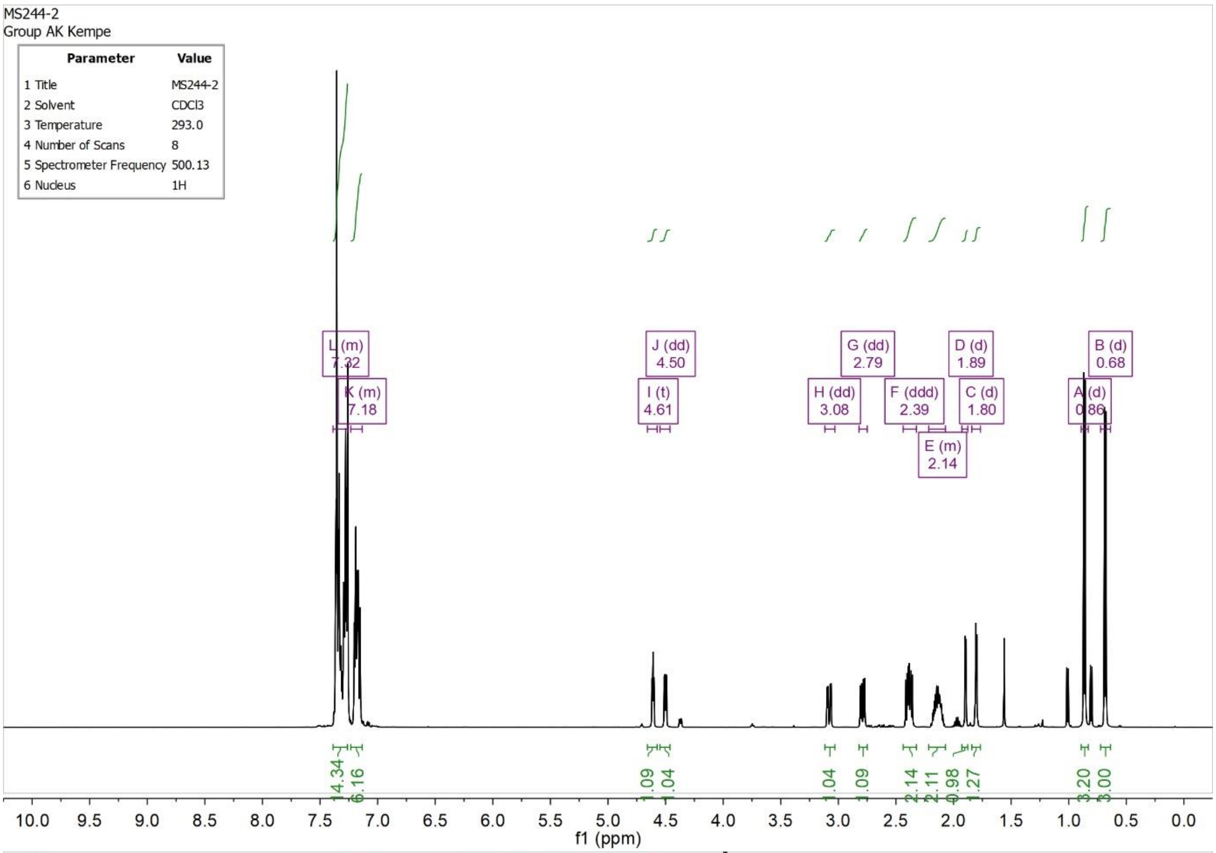

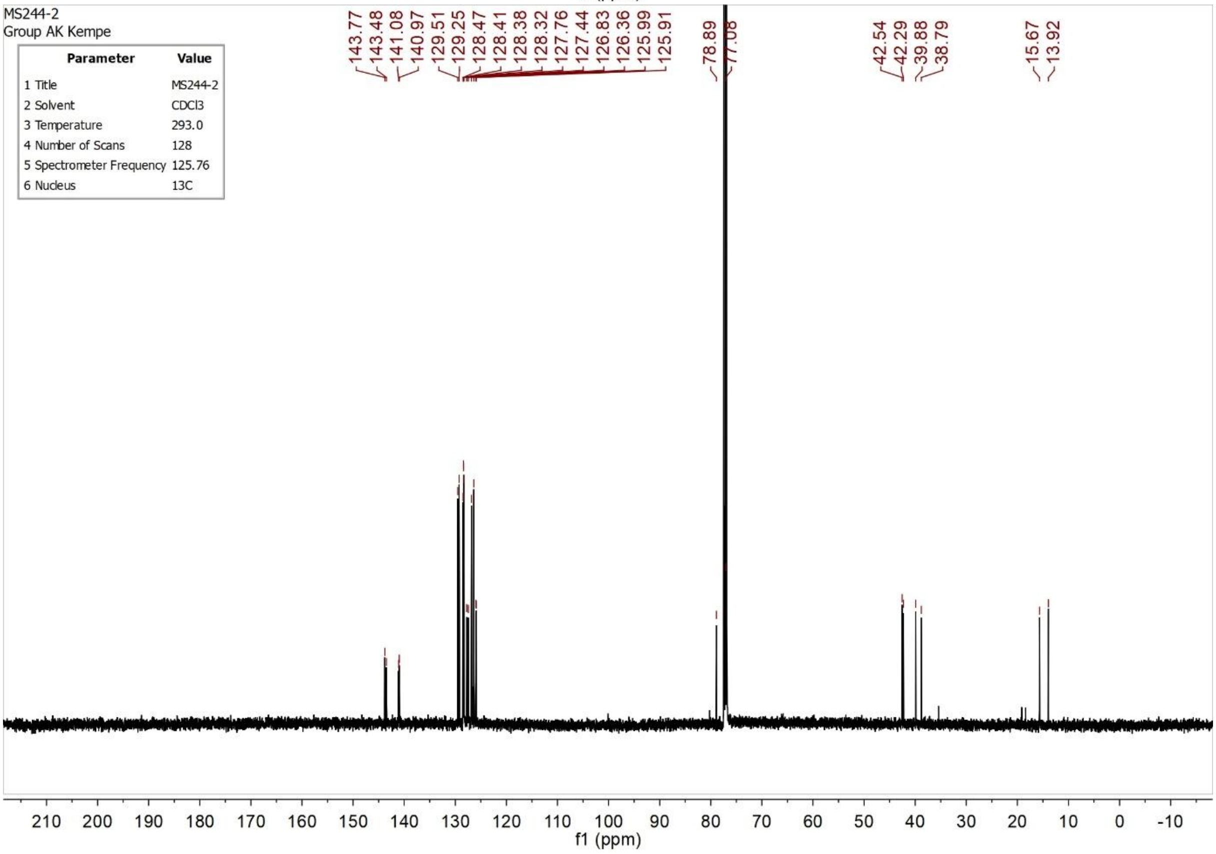

2-(methylamino)-1-phenylpropan-1-ol (B25)

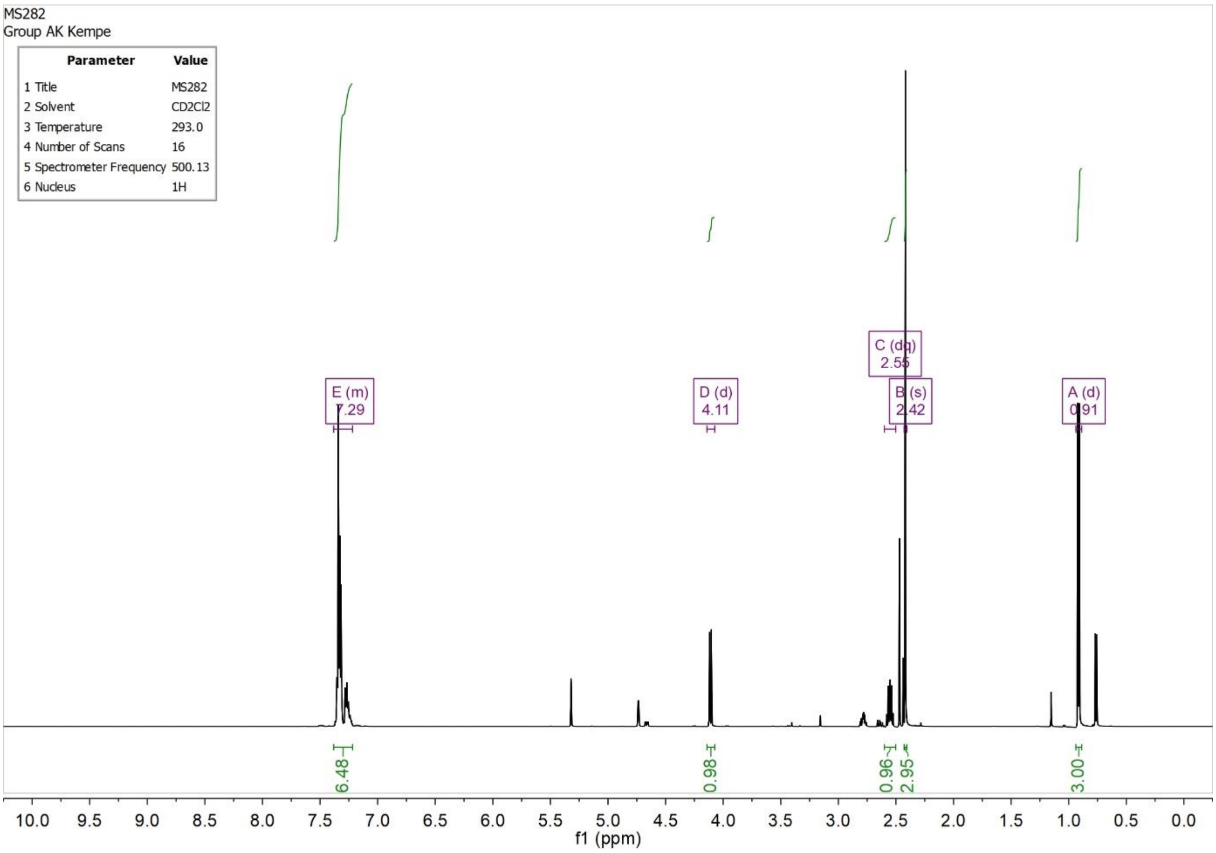

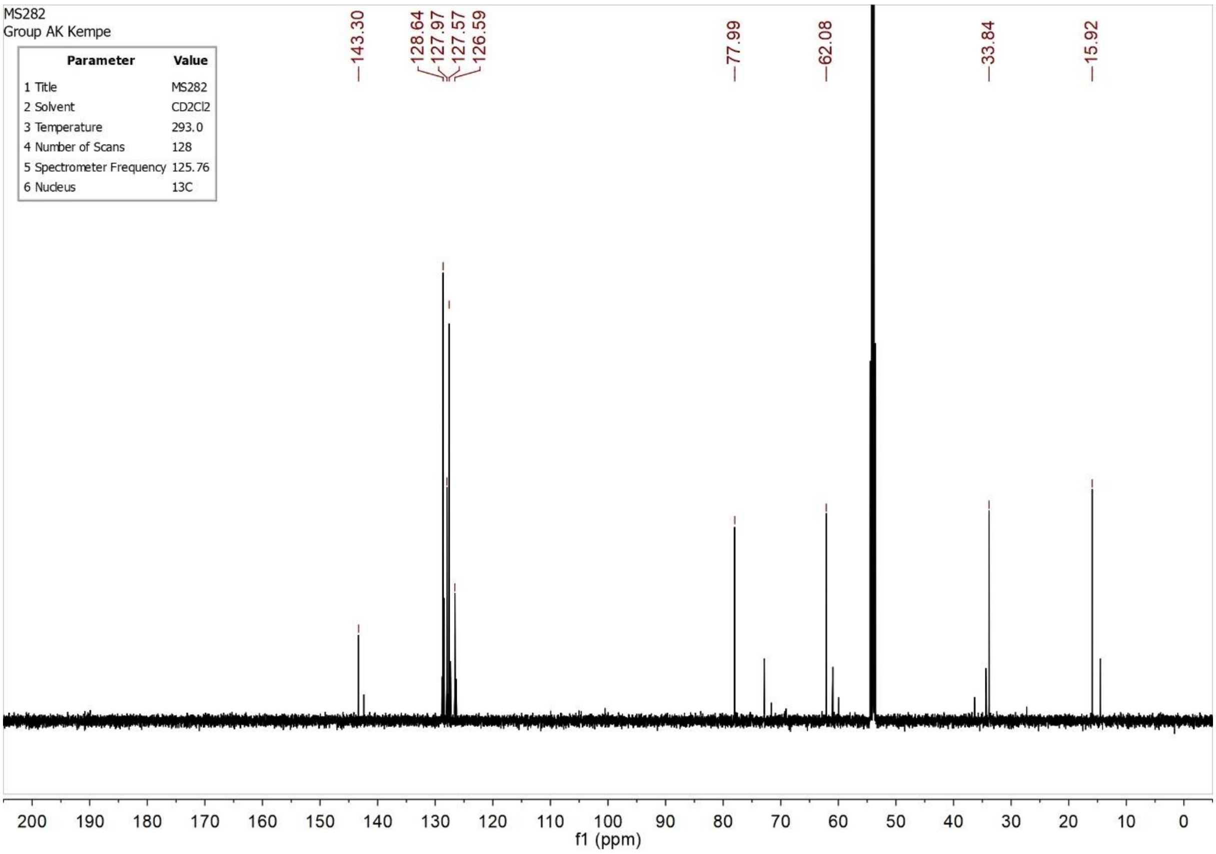

1-phenyl-2-(piperidin-1-yl)propan-1-ol (B26)

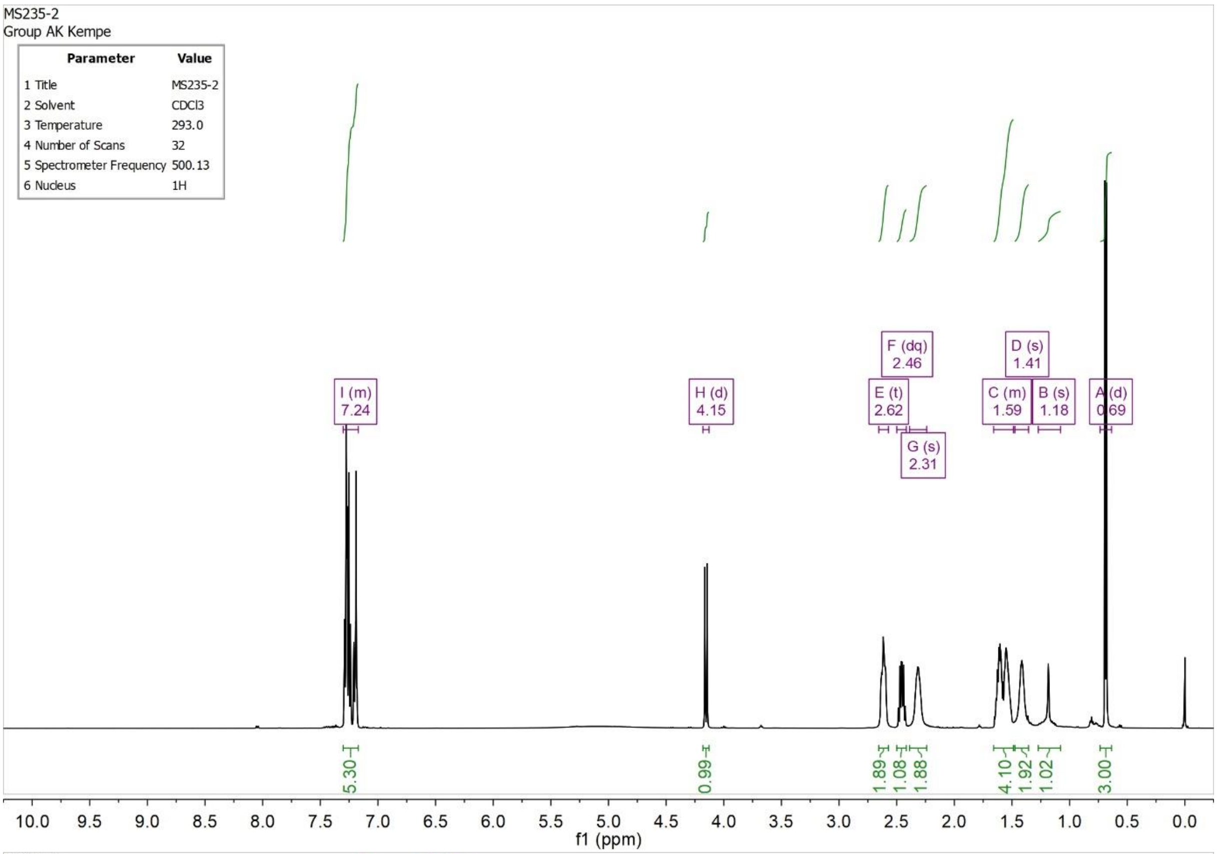

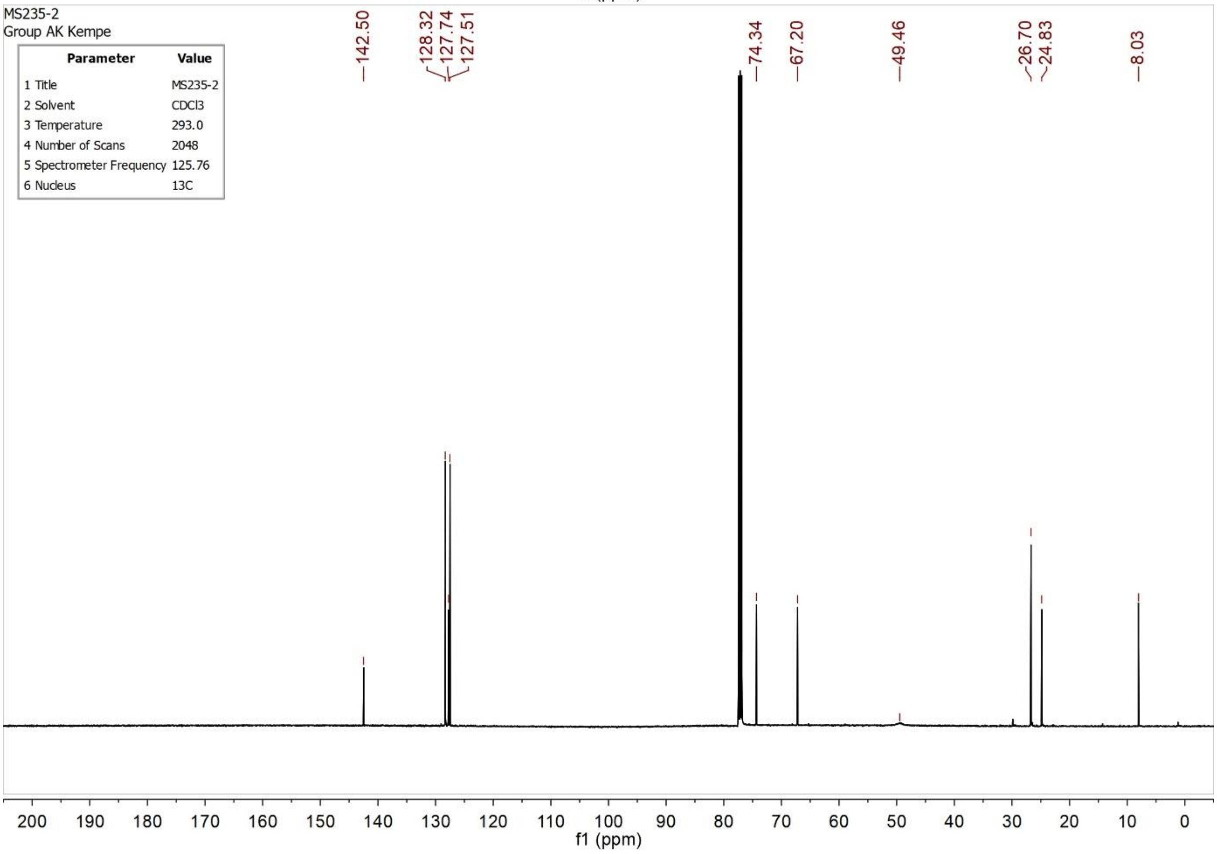

2-(cyclohexylamino)-6-methylcyclohexan-1-ol (B27)

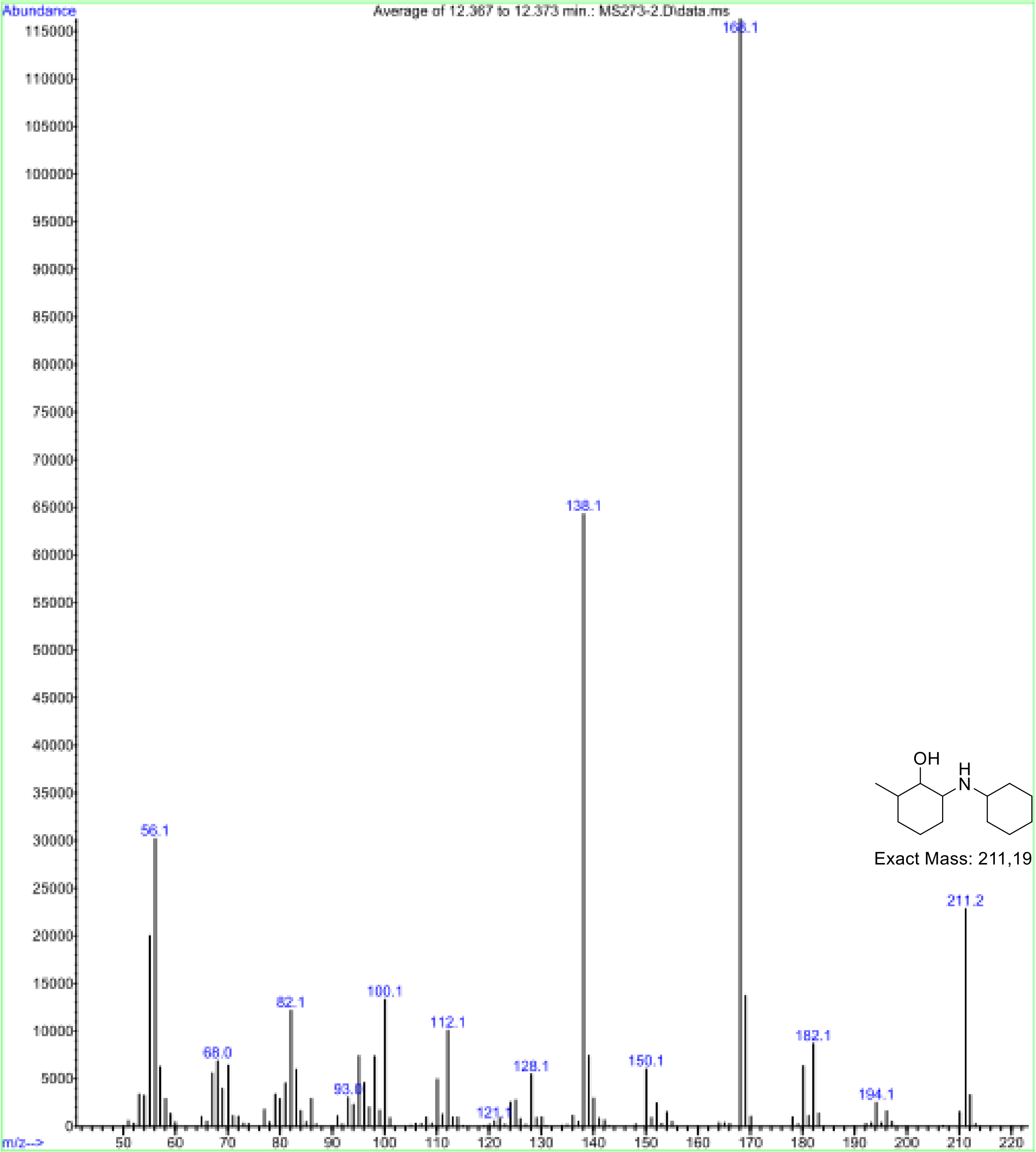

2-(4-methoxyphenyl)propan-1-ol (B28)

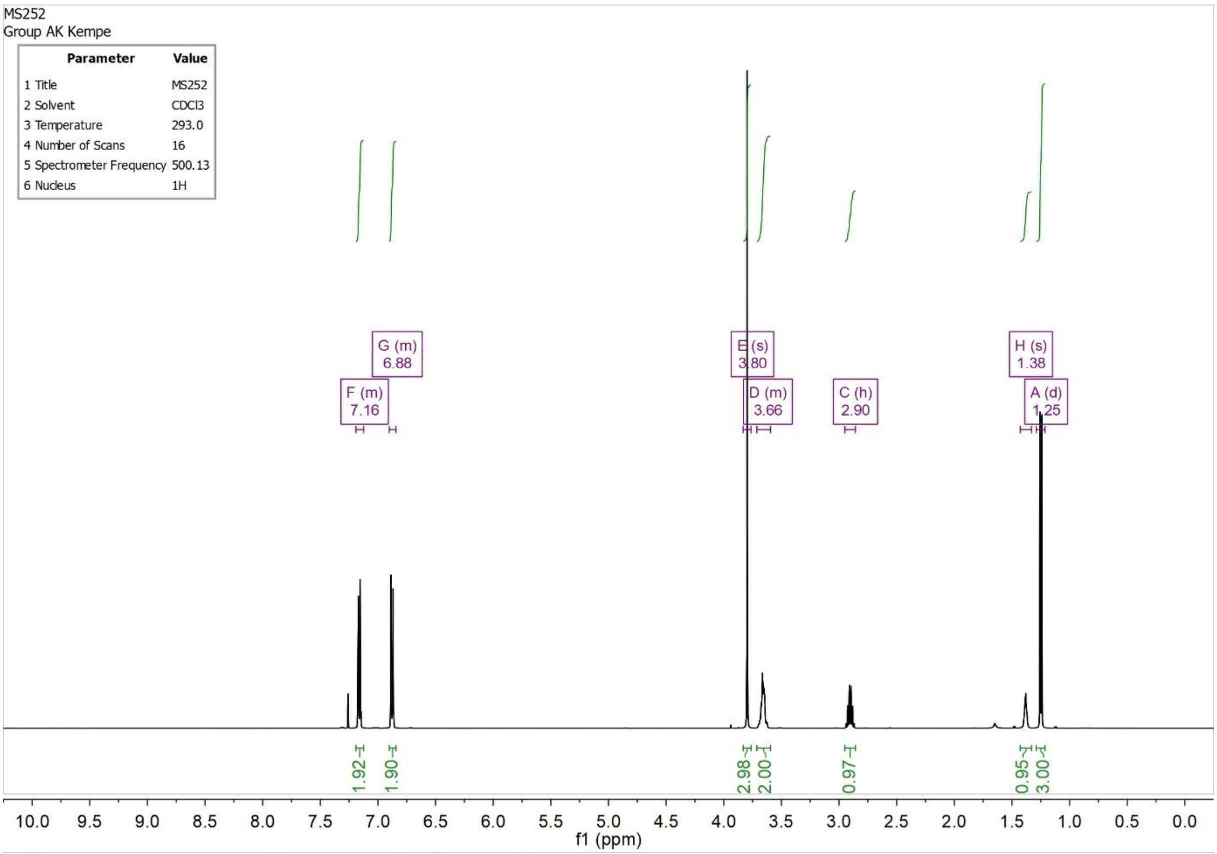

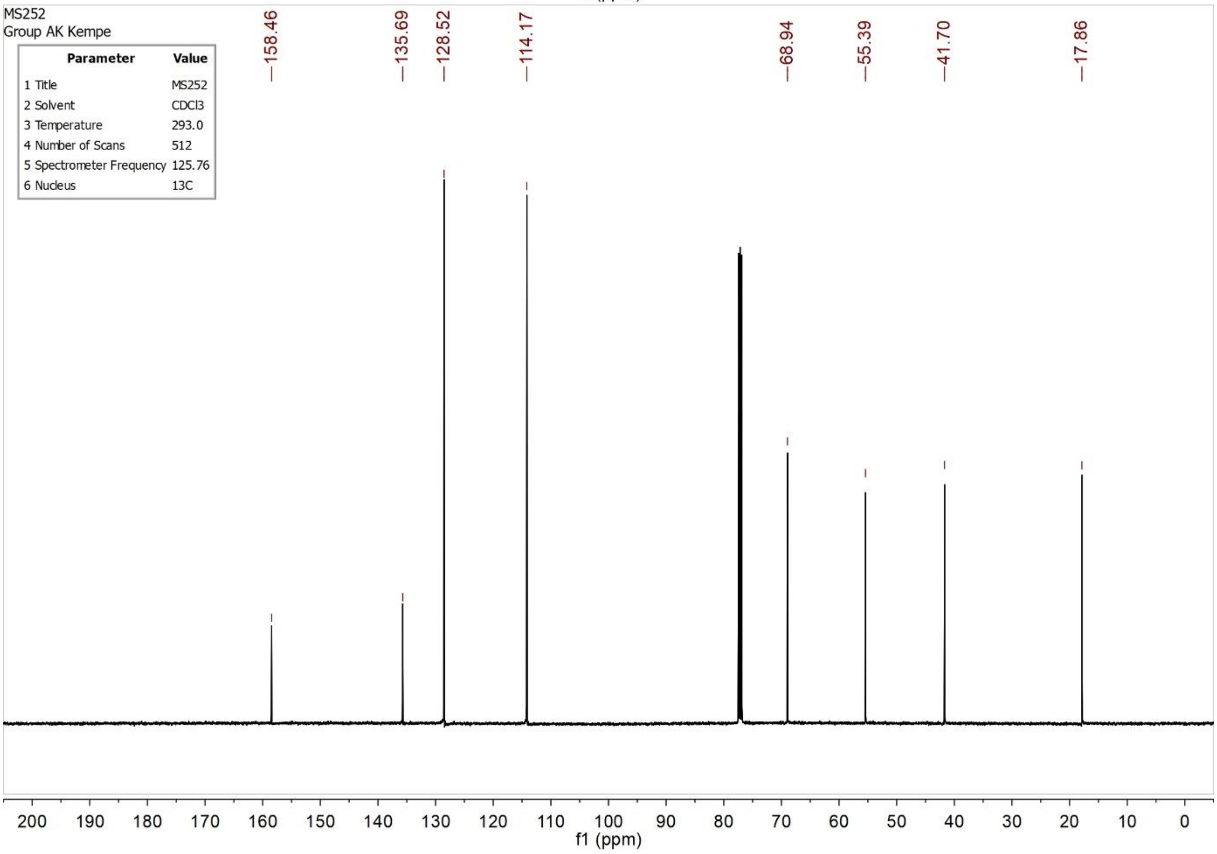

2-methyl-3-phenylpropan-1-ol (B29)

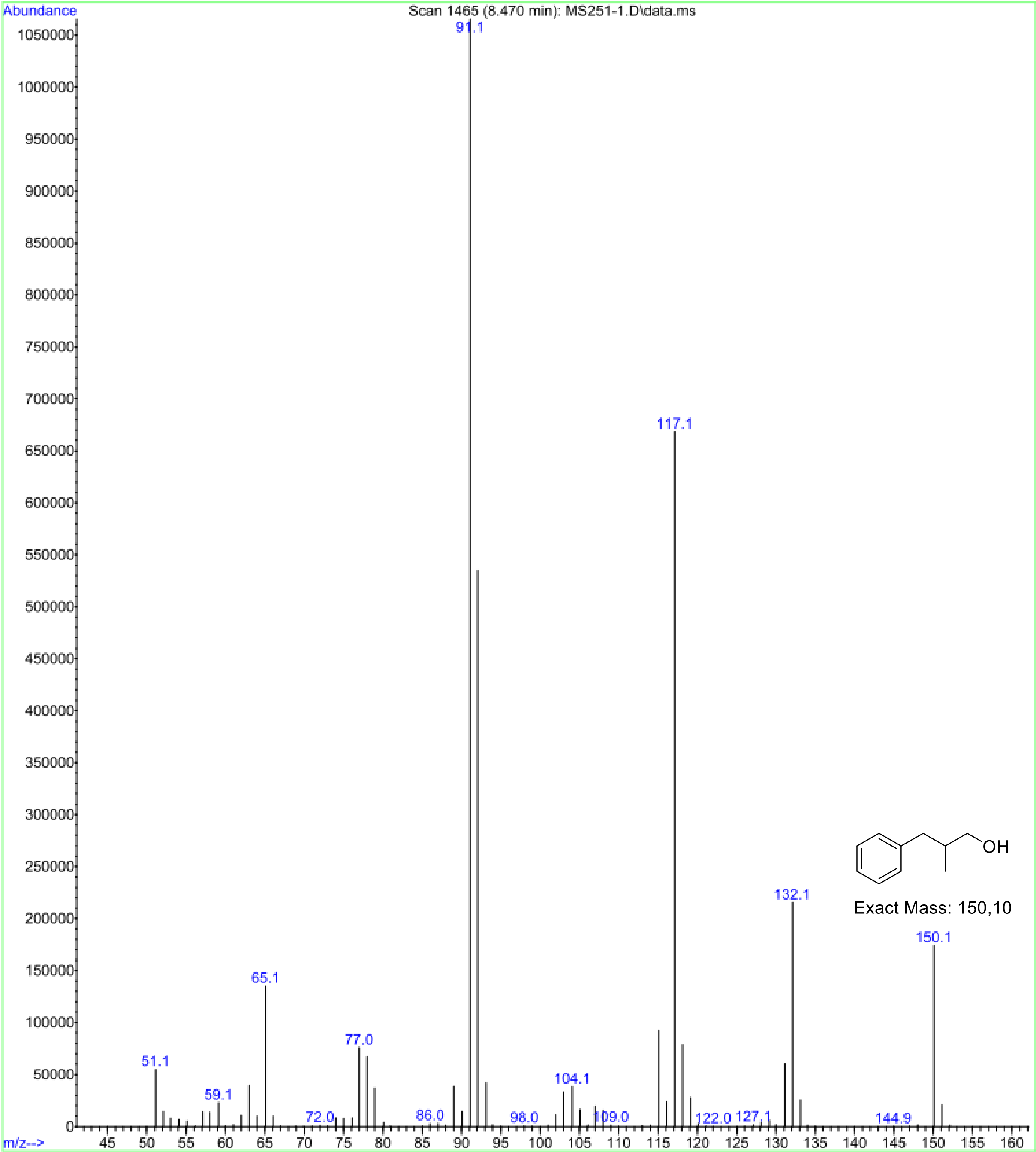

2,3,7-trimethyloct-6-en-1-ol (B30)

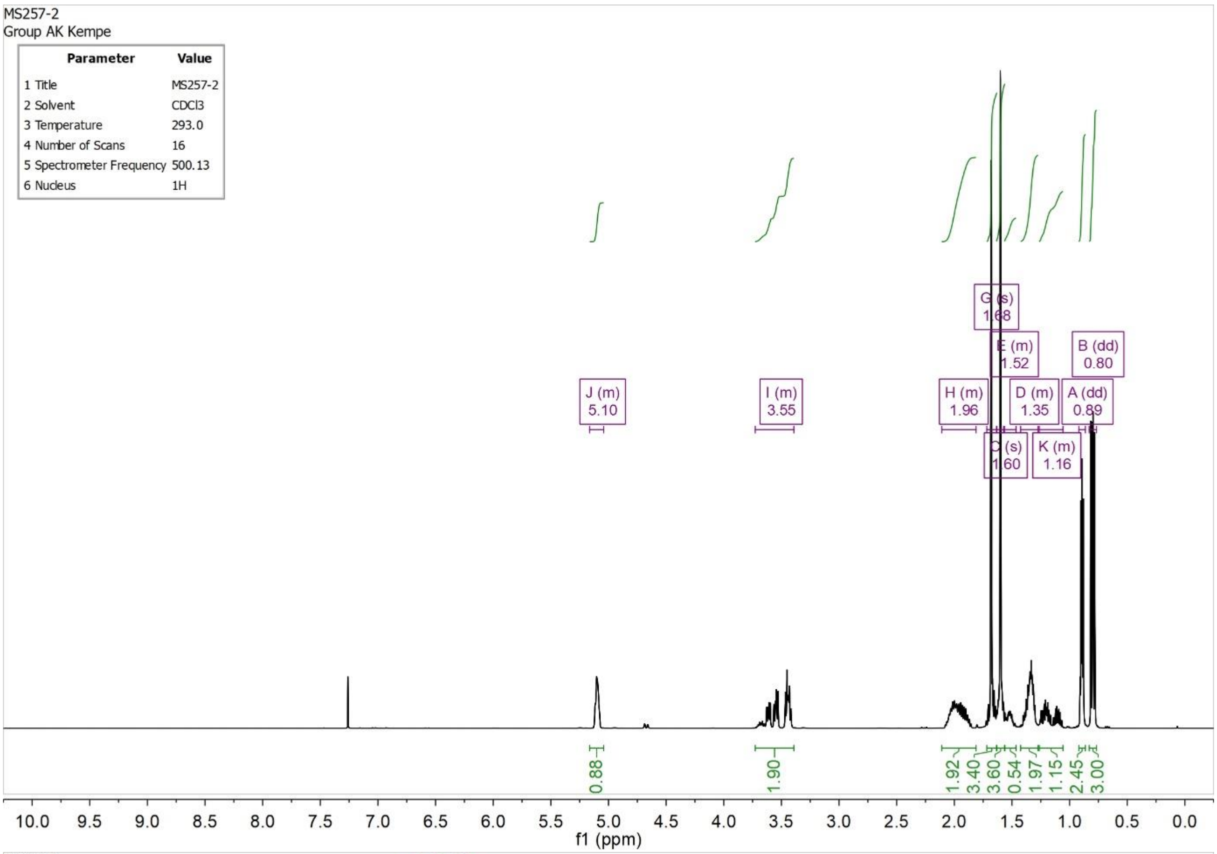

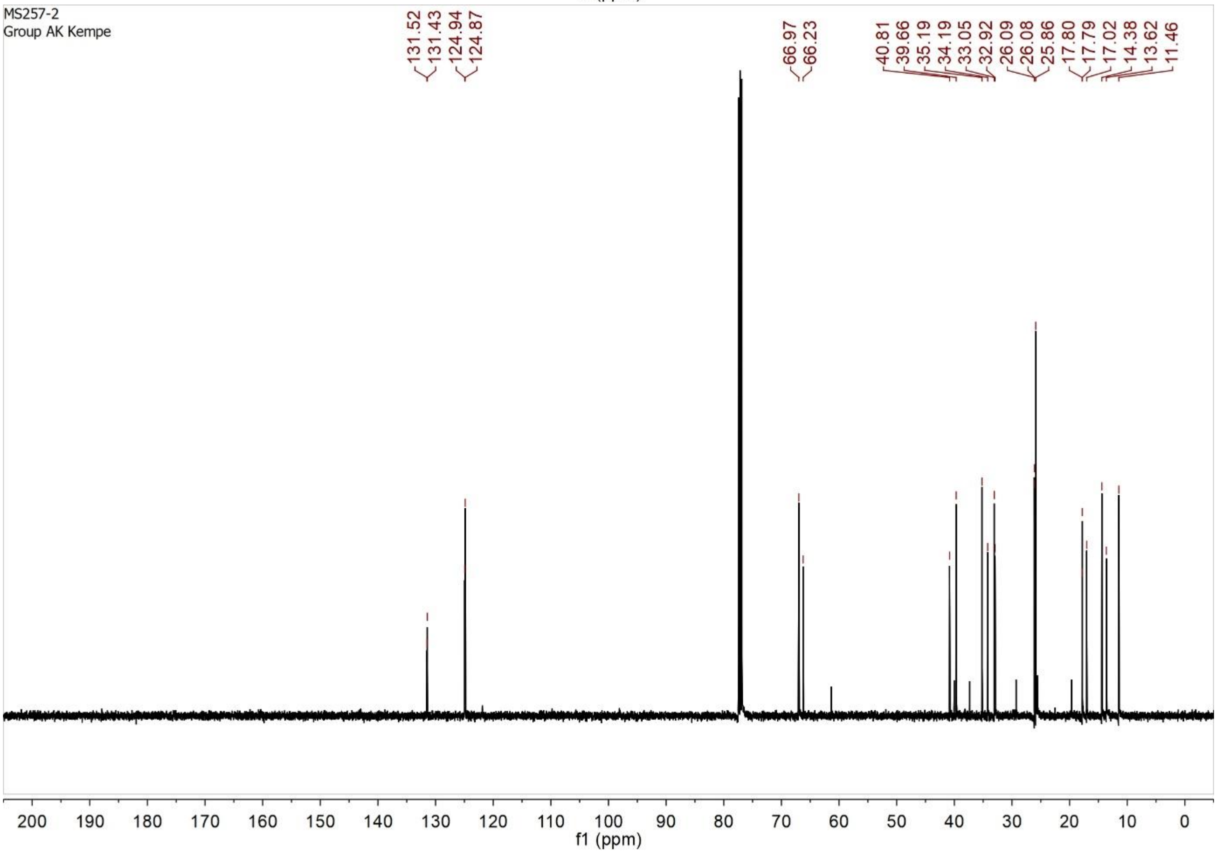

2,4-dimethylnonan-3-ol (B31)

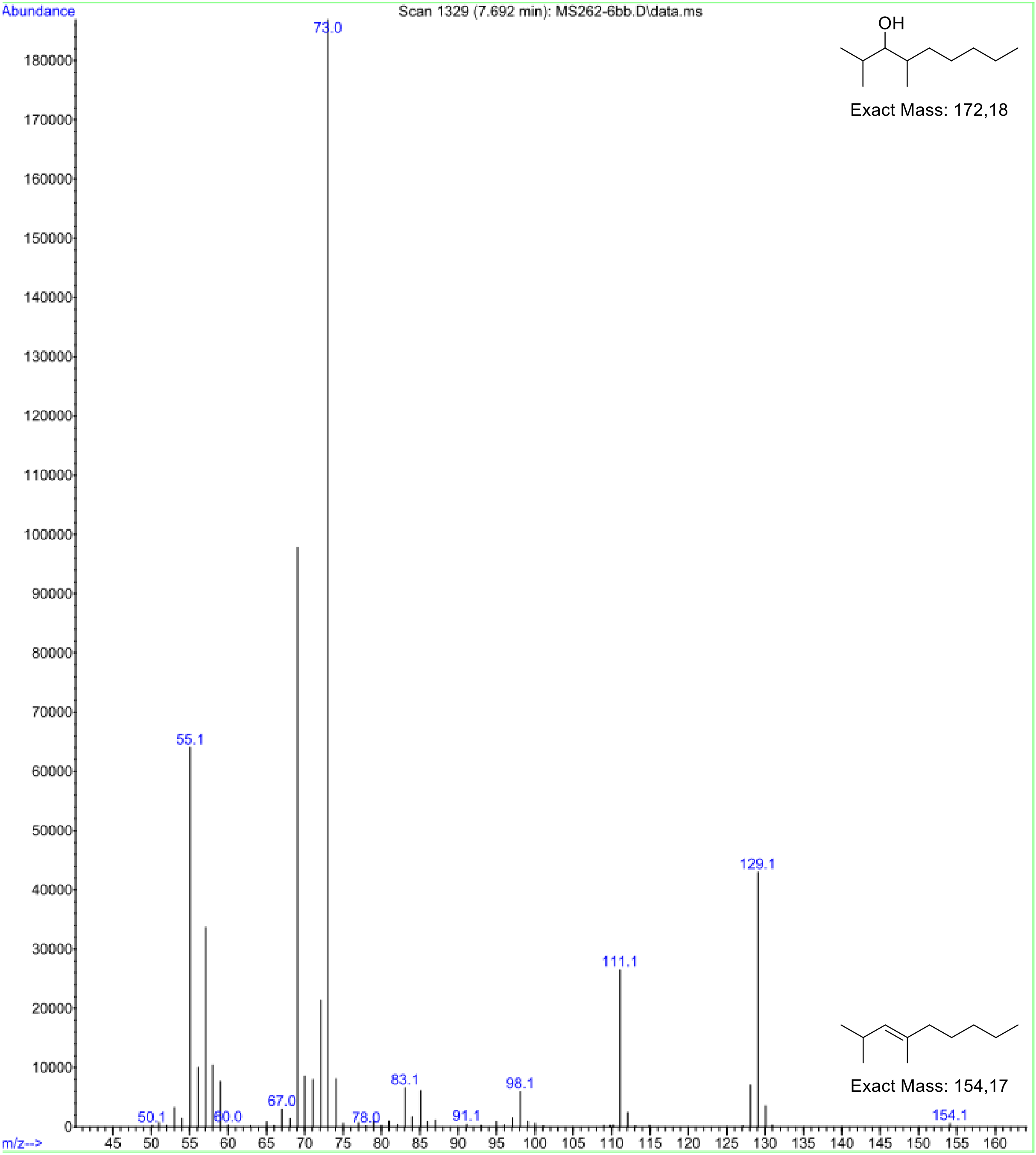

2,12-dimethylcyclododecan-1-ol (B32)

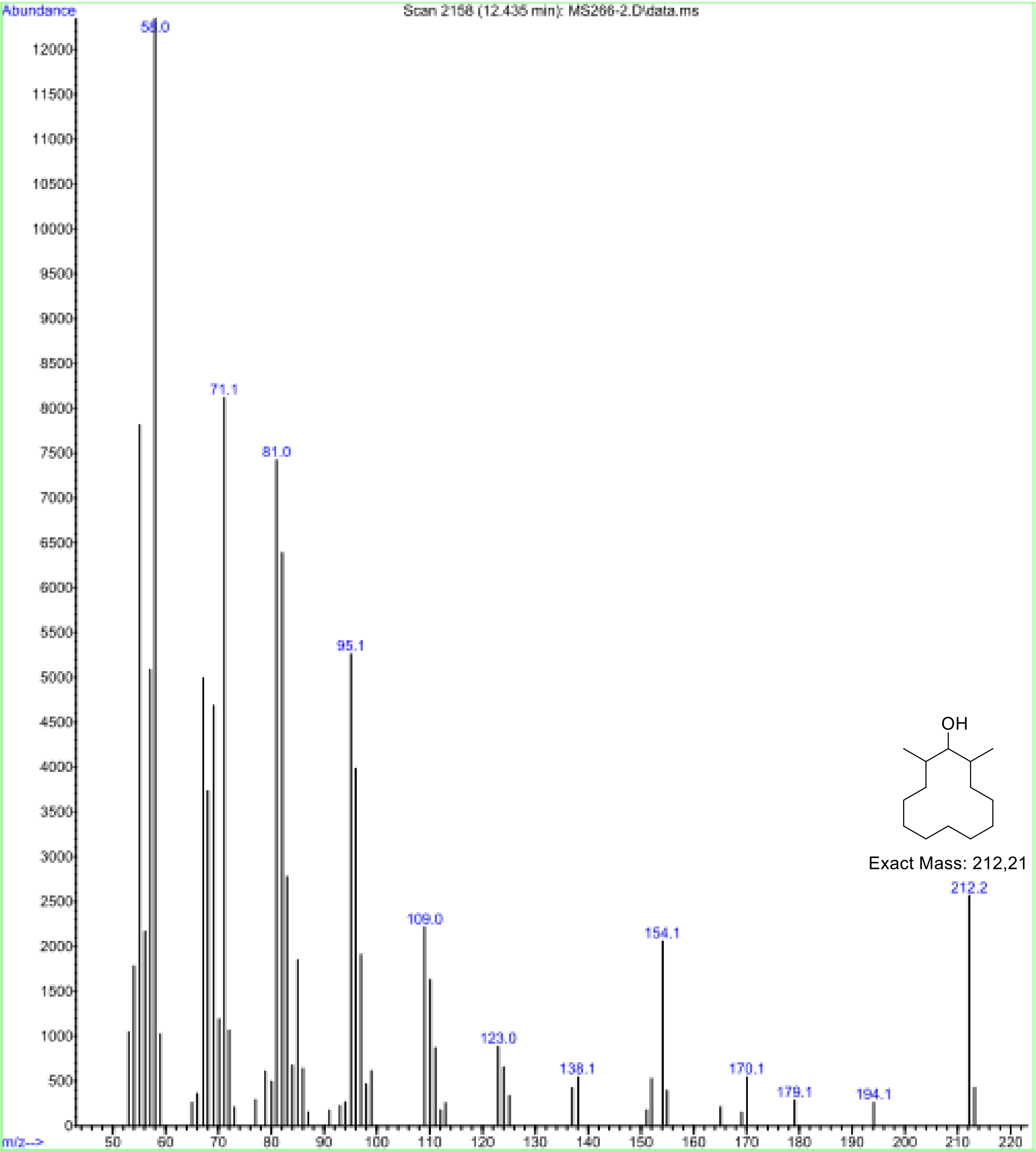

Crystallographic data

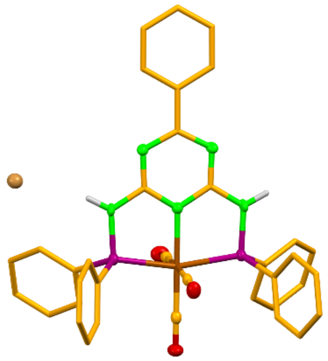

Compound	[Mn-IIIa]
Formula	$C_{36}H_{27}MnN_5O_3P_2Br$
Formula weight	774.41
Crystal system	orthorhombic
Space group	P b c m (57)
a [Å]	11.220(2)
b [Å]	12.830(3)
c [Å]	22.930(5)
α [°]	90
β [°]	90
γ [°]	90
Cell volume [$Å^3$]	3300.8(11)
Z	4
Crystal size [mm^3]	0.134*0.049*0.002
Habit	plate
Colour	colourless
Density [gcm^{-1}]	1.558
T [K]	133
Theta range	2.996 - 28.472
Unique reflections	3988
Observed reflections [I > 2s(I)]	2448
Parameters	234
*wR*2 all data	0.1015
R [I > 2s(I)]	0.0477

Checkcif report

[Mn-IIIa]

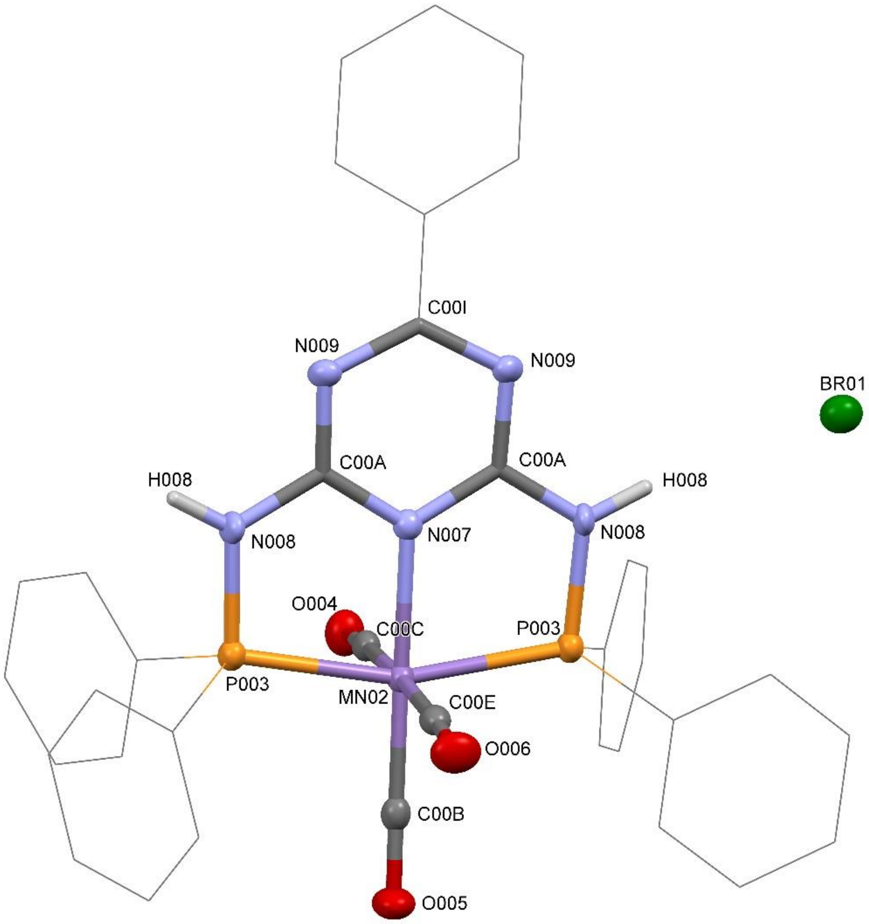

checkCIF/PLATON report

Structure factors have been supplied for datablock(s) sv541_1_o_pbcm_a2

THIS REPORT IS FOR GUIDANCE ONLY. IF USED AS PART OF A REVIEW PROCEDURE FOR PUBLICATION, IT SHOULD NOT REPLACE THE EXPERTISE OF AN EXPERIENCED CRYSTALLOGRAPHIC REFEREE.

No syntax errors found. CIF dictionary Interpreting this report

Datablock: sv541_1_o_pbcm_a2

```
Bond precision:  C-C = 0.0052 A          Wavelength=0.71073

Cell:             a=11.220(2)      b=12.830(3)      c=22.930(5)
                  alpha=90         beta=90          gamma=90
Temperature:      133 K

                Calculated                 Reported
Volume          3300.8(12)                 3300.8(11)
Space group     P b c m                    P b c m
Hall group      -P 2c 2b                   -P 2c 2b
Moiety formula  C36 H27 Mn N5 O3 P2, Br    Br, C36 H27 Mn N5 O3 P2
Sum formula     C36 H27 Br Mn N5 O3 P2     C36 H27 Br Mn N5 O3 P2
Mr              774.41                     774.41
Dx,g cm-3       1.558                      1.558
Z               4                          4
Mu (mm-1)       1.753                      1.753
F000            1568.0                     1568.0
F000'           1569.49
h,k,lmax        15,17,30                   14,17,30
Nref            4274                       3988
Tmin,Tmax       0.902,0.997                0.926,0.965
Tmin'           0.791

Correction method= # Reported T Limits: Tmin=0.926 Tmax=0.965
AbsCorr = NUMERICAL

Data completeness= 0.933          Theta(max)= 28.472

R(reflections)= 0.0477( 2448)     wR2(reflections)= 0.1015( 3988)

S = 0.975                 Npar= 234
```

The following ALERTS were generated. Each ALERT has the format
test-name_ALERT_alert-type_alert-level.
Click on the hyperlinks for more details of the test.

It is advisable to attempt to resolve as many as possible of the alerts in all categories. Often the minor alerts point to easily fixed oversights, errors and omissions in your CIF or refinement strategy, so attention to these fine details can be worthwhile. In order to resolve some of the more serious problems it may be necessary to carry out additional measurements or structure refinements. However, the purpose of your study may justify the reported deviations and the more serious of these should normally be commented upon in the discussion or experimental section of a paper or in the "special_details" fields of the CIF. checkCIF was carefully designed to identify outliers and unusual parameters, but every test has its limitations and alerts that are not important in a particular case may appear. Conversely, the absence of alerts does not guarantee there are no aspects of the results needing attention. It is up to the individual to critically assess their own results and, if necessary, seek expert advice.

Publication of your CIF in IUCr journals

A basic structural check has been run on your CIF. These basic checks will be run on all CIFs submitted for publication in IUCr journals (*Acta Crystallographica*, *Journal of Applied Crystallography*, *Journal of Synchrotron Radiation*); however, if you intend to submit to *Acta Crystallographica Section C* or *E* or *IUCrData*, you should make sure that full publication checks are run on the final version of your CIF prior to submission.

Publication of your CIF in other journals

Please refer to the *Notes for Authors* of the relevant journal for any special instructions relating to CIF submission.

PLATON version of 07/08/2019; check.def file version of 30/07/2019

Alert level C

```
ABSTY02_ALERT_1_C  An _exptl_absorpt_correction_type has been given without
            a literature citation. This should be contained in the
            _exptl_absorpt_process_details field.
            Absorption correction given as numerical
PLAT906_ALERT_3_C Large K Value in the Analysis of Variance ......      3.207 Check
PLAT910_ALERT_3_C Missing # of FCF Reflection(s) Below Theta(Min).          5 Note
PLAT911_ALERT_3_C Missing FCF Refl Between Thmin & STh/L=    0.600         17 Report
```

Alert level G

```
PLAT007_ALERT_5_G Number of Unrefined Donor-H Atoms .............           1 Report
PLAT042_ALERT_1_G Calc. and Reported MoietyFormula Strings  Differ     Please Check
PLAT180_ALERT_4_G Check Cell Rounding: # of Values Ending with 0 =          3 Note
PLAT304_ALERT_4_G Non-Integer Number of Atoms in ...... Resd  2          0.50 Check
PLAT720_ALERT_4_G Number of Unusual/Non-Standard Labels .........          44 Note
PLAT793_ALERT_4_G Model has Chirality at P003        (Centro SPGR)          R Verify
PLAT794_ALERT_5_G Tentative Bond Valency for Mn02       (I)        .     1.11 Info
PLAT912_ALERT_4_G Missing # of FCF Reflections Above STh/L=  0.600        260 Note
PLAT933_ALERT_2_G Number of OMIT Records in Embedded .res File ...          5 Note
PLAT978_ALERT_2_G Number C-C Bonds with Positive Residual Density.          1 Info
```

0 **ALERT level A** = Most likely a serious problem - resolve or explain
0 **ALERT level B** = A potentially serious problem, consider carefully
4 **ALERT level C** = Check. Ensure it is not caused by an omission or oversight
10 **ALERT level G** = General information/check it is not something unexpected

2 ALERT type 1 CIF construction/syntax error, inconsistent or missing data
2 ALERT type 2 Indicator that the structure model may be wrong or deficient
3 ALERT type 3 Indicator that the structure quality may be low
5 ALERT type 4 Improvement, methodology, query or suggestion
2 ALERT type 5 Informative message, check

Datablock sv541_1_o_pbcm_a2 - ellipsoid plot

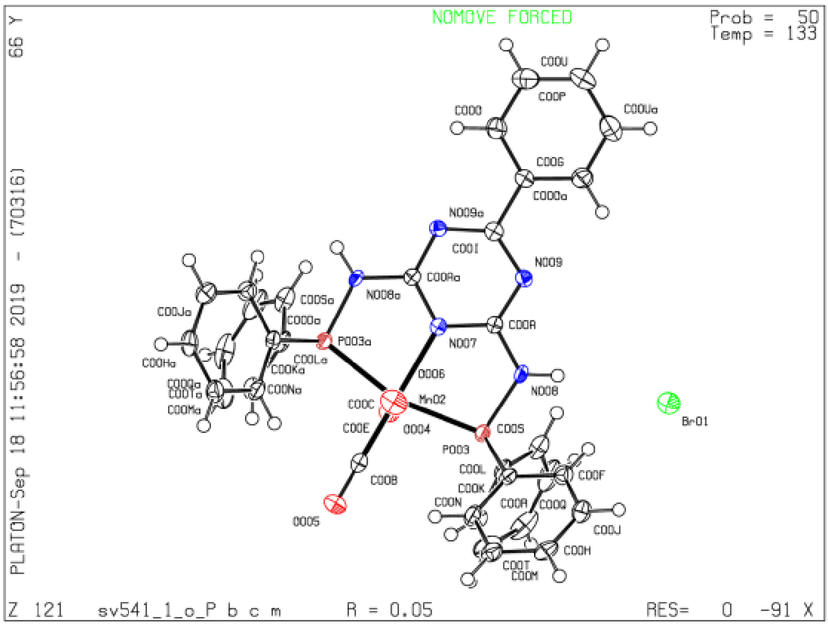

Supplement: Supplementary file 1 — Supplementary [file ANIE-59-1485-s001.pdf]
